# Detection of cryptogenic malignancies from metagenomic whole genome sequencing of body fluids

**DOI:** 10.1186/s13073-021-00912-z

**Published:** 2021-06-01

**Authors:** Wei Gu, Eric Talevich, Elaine Hsu, Zhongxia Qi, Anatoly Urisman, Scot Federman, Allan Gopez, Shaun Arevalo, Marc Gottschall, Linda Liao, Jack Tung, Lei Chen, Harumi Lim, Chandler Ho, Maya Kasowski, Jean Oak, Brittany J. Holmes, Iwei Yeh, Jingwei Yu, Linlin Wang, Steve Miller, Joseph L. DeRisi, Sonam Prakash, Jeff Simko, Charles Y. Chiu

**Affiliations:** 1 Department of Laboratory Medicine, University of California San Francisco, San Francisco, CA 94107 USA; 2 UCSF-Abbott Viral Diagnostics and Discovery Center, San Francisco, CA 91407 USA; 3 Department of Pathology, Stanford University, Stanford, CA 94305 USA; 4 Stanford Health Care, Stanford University, Stanford, CA 94305 USA; 5 DNANexus, Mountain View, CA 94040 USA; 6 Department of Pathology, University of California San Francisco, San Francisco, CA 94107 USA; 7 Department of Biochemistry and Biophysics, University of California San Francisco, San Francisco, CA 94107 USA; 8 Chan Zuckerberg Biohub, San Francisco, CA 94107 USA; 9 Department of Medicine, Division of Infectious Diseases, University of California San Francisco, San Francisco, CA 94107 USA

## Abstract

**Background:**

Metagenomic next-generation sequencing (mNGS) of body fluids is an emerging approach to identify occult pathogens in undiagnosed patients. We hypothesized that metagenomic testing can be simultaneously used to detect malignant neoplasms in addition to infectious pathogens.

**Methods:**

From two independent studies (n = 205), we used human data generated from a metagenomic sequencing pipeline to simultaneously screen for malignancies by copy number variation (CNV) detection. In the first case-control study, we analyzed body fluid samples (n = 124) from patients with a clinical diagnosis of either malignancy (positive cases, n = 65) or infection (negative controls, n = 59). In a second verification cohort, we analyzed a series of consecutive cases (n = 81) sent to cytology for malignancy workup that included malignant positives (n = 32), negatives (n = 18), or cases with an unclear gold standard (n = 31).

**Results:**

The overall CNV test sensitivity across all studies was 87% (55 of 63) in patients with malignancies confirmed by conventional cytology and/or flow cytometry testing and 68% (23 of 34) in patients who were ultimately diagnosed with cancer but negative by conventional testing. Specificity was 100% (95% CI 95–100%) with no false positives detected in 77 negative controls. In one example, a patient hospitalized with an unknown pulmonary illness had non-diagnostic lung biopsies, while CNVs implicating a malignancy were detectable from bronchoalveolar fluid.

**Conclusions:**

Metagenomic sequencing of body fluids can be used to identify undetected malignant neoplasms through copy number variation detection. This study illustrates the potential clinical utility of a single metagenomic test to uncover the cause of undiagnosed acute illnesses due to cancer or infection using the same specimen.

**Supplementary Information:**

The online version contains supplementary material available at 10.1186/s13073-021-00912-z.

## Background

Pathogen identification using metagenomic testing has recently been clinically implemented for patient care by our group and others [1–8]. While clinical metagenomic sequencing is often performed for patients who lack a definitive diagnosis to search for an infectious organism, the underlying disease may also be rooted in a non-infectious cause such as a malignant neoplasm. Detection of malignancies in various body fluids is primarily based on cytological analysis as the gold standard test. However, the estimated sensitivity for cytology is 60% for pleural fluid [9], 67% for peritoneal fluid in the context of ovarian carcinoma [10], and approaching undetectable for liver masses without concurrent peritoneal carcinomatosis [11].

**Fig. 1:**
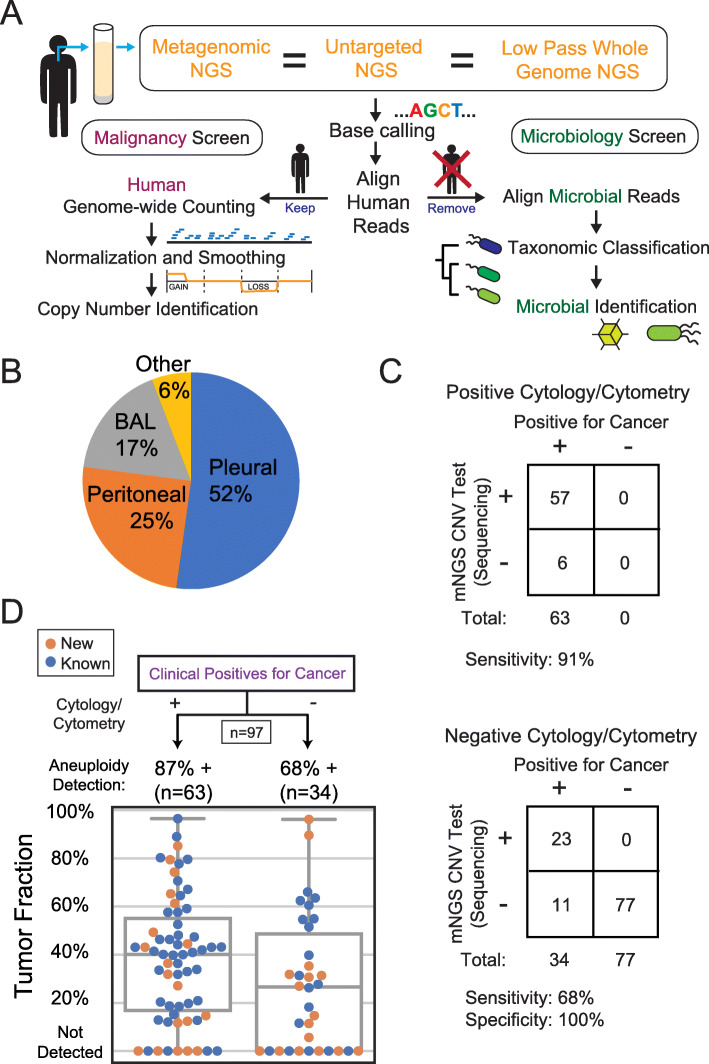
**A** Schematic of the bioinformatics pipeline. After whole genome sequencing of cell-free DNA from body fluids, adapter sequences are trimmed and aligned to the human genome. The cancer pipeline aligns human reads and counts reads over moving windows across the human genome [12, 17]. The microbial pipeline aligns non-human reads to a microbial database, taxonomically classifies the microbial aligned reads, and identifies pathogens [2, 18]. **B** Sample type composition of the 205 body fluid samples. **C** Contingency table comparing conventional cancer detection to sequencing in patients with malignancy. Negative controls did not have a history of cancer and were explained by infections with positive microbiological testing (top). Patients with cancer detected by positive cytology and/or flow cytometry testing of body fluid (bottom). Patients diagnosed with cancer but with negative or ambiguous detection based on conventional clinical testing in the same fluid by cytology or cytometry. **D** Detection accuracy and tumor fractions. Detection of malignancies through CNV detection in 2 cancer-positive case categories described in **C**. The “New” category refers to samples collected from patients with a new diagnosis who have no previous cancer history and have not been treated. Tumor fractions were estimated through the magnitude of copy changes detected (see online Methods, “Equation 1”)

By repurposing the residual human reads in metagenomic sequencing data from non-circulating fluids (e.g., pleural, peritoneal, respiratory fluids), we hypothesized that we would concurrently detect cancer associated CNVs using a depth of coverage method [12–17]. This method was previously used to detect fetal aneuploidy in non-invasive prenatal testing (NIPT) [12] and later cytogenetic aberrations in cancer (Fig. 1A) [13–16]. CNVs are ubiquitous in solid tumors, with aneuploidy alone present in ~ 90% of malignant tumors [19], making this an appealing broad range marker.

## Methods

### Sample selection

The first study incorporated residual body fluid samples sent to the UCSF Clinical Laboratories (San Francisco, CA, USA) between 2017 to 2019 for flow cytometry, cell count, chemistries, and microbiological testing. All samples matching inclusion criteria (see below) in a recent metagenomics study were used, except five samples were excluded because they had less than 450,000 reads [20]. Serial dilutions of the sample input and downsampling of sequencing reads suggested that results are interpretable down to 1.6 pg input and 276,000 reads (Additional file 1). A total of 65 cancer-positive and 59 cancer-negative samples were collected. The samples consisted of 62 (50%) pleural fluid, 31 (25%) peritoneal fluid, 24 (19%) bronchoalveolar lavage fluid, and 7 (6%) other body fluids. The positive cases were included from patients with a clinical diagnosis of cancer established either by definitive laboratory testing (cytology and/or flow cytometry of a body fluid), tissue biopsy (“histologically confirmed”), or by the treating physician on the basis of history, presentation, radiographic imaging, and supportive laboratory testing results (“histologically unconfirmed”). Patients lacking a clear diagnosis after long-term follow-up were excluded. Patients who were being actively treated for malignancy at the time of sample collection and not positive by cytology or cytometry were excluded. Negative controls were taken from the prior metagenomics study [20], and we included patients with a microbiologically proven infection, who lacked clinical history of cancer, and who were negative for malignancy by cytology and cytometry.

The second study analyzed all consecutively available body fluid samples sent to Stanford clinical laboratories over 2.5 months in 2020 for cytologic testing. There was a total of 81 consecutive cases comprised of 56% pleural, 19% peritoneal, 14% bronchoalveolar lavage, 4% pericardial, and 2% fine needle aspirate. The residual samples were categorized similarly to the first study for positive cases and negative controls. However, the negative controls also included non-microbiological diagnoses by the treating physician. All available samples from cytology were included, except for those with insufficient volumes of less than 0.5 mL and those received outside of working hours.

### Body fluid sample extraction

Body fluid specimens were centrifuged at 16,000*g* for 10 min, and the supernatant was stored at – 80 °C. In the first study, nucleic acid extraction was performed by the EZ1 Advanced XL BioRobot using the EZ1 Virus Mini Kit v2.0 (QIAGEN) with 400 μL input and 60 μL output. In the second study, nucleic acid extraction was performed using the Maxwell RSC ccfDNA Plasma Kit (Promega) with 1000 μL input and 50 μL output.

### Body fluid library preparation

Whole genome sequencing (WGS) library preparation was performed using the NEBNext Ultra II DNA Library Prep Kit (New England Biolabs) on a liquid handler (first study: epMotion 5075 Eppendorf, second study: Hamilton STARlet) using the manufacturer’s protocol unless otherwise stated. All reagent usage was halved, and the input was also halved to 25 μL of extracted DNA. For bead purification, we used Ampure XP beads (Beckman Coulter) or Mag-Bind TotalPure beads (Omega Biotek) in the first and second study respectively. PCR amplification of the adapter ligated DNA was up to 26 cycles using the manufacturer’s protocol, and we used primers with dual indexing. Sequencing was performed on an Illumina HiSeq 1500/2500, Nextseq 550, or Novaseq using the single-end or paired-end rapid run configuration set at 1 × 140 bp or 2 × 140 bp. Only samples with more than 450 thousand reads were considered for this study.

### Tissue extraction and library preparation

Formalin fixed paraffin blocks were used to obtain correlated CNV data from cancer tissue obtained from the same patient. All archival tissue was no longer needed for clinical care. A pathologist (J.S.) identified regions of high tumor content on correlated tissue section(s). A disposable dermal punch was used to either punch out or scrape tissue from regions of interest. This fixed tissue was extracted for nucleic acids using the Quick-DNA FFPE Miniprep kit (Zymo Research). Each sample was sheared using focused acoustics to approximately 250 bp in a microTUBE (Covaris) and quantified on a spectrometer (Nanodrop, Thermo Fisher). About 100 ng was used for WGS library preparation as described above.

### FISH

Abbott Vysis LSI D7S486/CEP7, CEP8, and D20S108 probe sets were used for detecting deletion of chromosome 7q/loss of a chromosome 7, gain of a chromosome 8, and deletion of chromosome 20q, respectively. These probes were ordered from Abbott Molecular (Des Plaines, IL). FISH was performed following a standard protocol (https://www.molecular.abbott/us/en/vysis-fish-knowledge-center). Interphase cells were counterstained using DAPI II (Abbott Molecular) and FISH results were analyzed using the CytoVision system (Leica Microsystems, San Jose, CA).

### Informatics

Raw data was demultiplexed to raw FASTQ files and adapter trimmed with cutadapt (v1.16). The metagenomic pipeline used SURPI [2, 18] for pathogen detection from metagenomic sequencing data. Raw copy ratio plots were created by deduplicating metagenomic reads with BWA [21] (v0.7.12) and aligning deduplicated reads to the human genome hg38 and. CNVkit [17] (v0.9.1) was used to display a log2 copy ratio across all genomic bins and infer discrete copy number segments using the default circular binary segmentation algorithm (orange in plots). Body fluid samples were normalized to a plasma sample from a healthy male. Correlated tissue samples were normalized to a resected tonsil from an otherwise healthy boy undergoing tonsillectomy due to an infection.

To determine NGS positives, a molecular pathologist (JT) was blinded to and not involved with gold standard determination, sample collection, preparation, and copy ratio plotting. The pathologist identified samples with copy ratio plots showing at least one significant CNV(s) (> 10 Mbp) across all chromosomes with the exception of the entirety of the sex chromosomes (differences in sex were not accounted for) and chromosome 19 due to its GC rich content and known tendency to appear more noisy than all other chromosomes [12, 22]. Chromosome 19 was used as a metric for the extent of noise on a per sample basis, typically for samples with low DNA content. Smaller telomere and centromere regions and deviations from diploid that are gradual rather than abrupt were both interpreted with caution. Individually binned copy ratios (gray dots in plots) were primarily used rather than the results of segmentation algorithms (orange/red line in plots). Before interpreting the second study, the interpreter was able to review the gold standard for the first study that was already interpreted.

The tumor fraction (Equation 1) was estimated from the log 2 ratio of the sample to the diploid control copy number. An assumption is made that certain deletions were haploid (e.g., monosomy) or that certain gains were haploid (e.g., trisomy).

1 $$ Tumor\ Fraction=\frac{1-{2}^{\left(\log 2\  ratio\right)}}{1-\frac{\left( assumed\ ploidy\right)}{2}} $$

## Results

### Test performance study

A total of 65 cancer-positive and 59 cancer-negative samples were collected from University of California San Francisco (UCSF) Medical Center. The samples consisted of 62 (50%) pleural fluid, 31 (25%) peritoneal fluid, 24 (19%) bronchoalveolar lavage fluid, and 7 (6%) other body fluids (Fig. 1B). Samples were from patients who were hospitalized (78% of positives and 94% of negatives) and who all presented with symptoms that warranted a diagnostic workup, including cytology and other laboratory testing of the body fluid. Metagenomic whole genome sequencing was performed on physiologically fragmented DNA yielding a median of 7.6 million reads (IQR 4.6–11.2 M) per sample with the vast majority of reads (> 95%) consistently aligning to human host cell-free DNA.

To provide an initial assessment of the test sensitivity, we analyzed the genomic human DNA reads for large (> 10 Mbp) CNVs in 36 cases that were positive for malignancy based on the conventional testing of the sample using cytology and/or flow cytometry. CNVs were called based on blinded interpretation of algorithmically generated copy ratio plots while considering the deviation of the copy ratio from diploid against background noise among other factors (see the “Methods” section). Of these cases, 31 of these had detectable CNVs at a sensitivity of 86% (95% CI 71–95%, Clopper-Pearson method) (Fig. 1C, Additional file 2: Table S1). The median tumor fraction of all 36 cases was 43% (IQR 25–59%) based on Equation 1 in the “Methods” section (Fig. 1D).

To better estimate the diagnostic sensitivity of body fluid CNV testing in the undiagnosed patient population, we analyzed additional cases where (i) cytology and/or flow cytometry results were negative (benign) or inconclusive (e.g., atypical cells), and (ii) a malignancy was eventually diagnosed through a subsequent tissue biopsy or as a histologically unconfirmed clinical diagnosis (Table 1). Patients lacking a diagnosis after long-term follow-up or were actively treated for malignancy at the time of sample collection were also excluded. Out of 29 such cases, CNVs were still detected in 19 at a sensitivity of 66% (95% CI 46–82%) (Fig. 1C, Table 1). The median tumor fraction was 30% (IQR 1.4–56%) (Fig. 1D). Both the sensitivity and the tumor fraction were lower when cytology/cytometry were negative, but unexpectedly high considering that conventional testing was not able to detect the malignancy. We therefore sought to confirm the positive CNV findings further by matching the CNVs in the body fluid and correlated cancer tissue from the same patient. In all 12 cases (out of these 19) for which clinical cytogenetic or molecular testing of the tumor was available, the CNVs found in the body fluid matched those in the associated cancer tissue (Additional file 1).

**Table 1:** Positive for cancer but negative by conventional testing (cytology/flow cytometry)

Sample ID	Sample type	Presentation	Cancer type	New Dx^a^	Cytology (Flow cytometry if available)	Confirmation	NGS Pos for CNVs and EBV	CNV count by NGS	Tumor Fraction
PC37	BAL	New lung nodules after chemotherapy, fever	Anaplastic large cell lymphoma	−	n/a	EBUS/FNA	−	0	0.00
PC38^b^	Pleural	Exudative pleural effusion, fever	Sarcoma	−	Benign	Tissue CNV correlation, clinical suspicion	+	5+	0.62
PC39^b^	Pleural	Lung masses (history of Leiomyosarcoma on chemotherapy), exudative pleural effusion, tachycardia, leukocytosis, dyspnea	Leiomyosarcoma	−	Benign	Tissue CNV correlated with NGS CNVs; imaging; clinical history	+	5+	0.28
PC40	Peritoneal	Lung and liver metastasis, peritonitis (low SAAG/low protein), fever	Poorly differentiated carcinoma most consistent with neuroendocrine carcinoma	−	Benign	Autopsy	+	5+	0.12
PC41^b^	Pleural	Lung masses, pleural effusion, lymphadenopathy, hypotension, dyspnea	Undifferentiated carcinoma	+	Benign (negative)	Tissue based FoundationONE testing: MET amplification correlated with NGS amplification	+	5+	0.056
PC42	Pleural	lymphadenopathy (cervical/thoracic), non-diagnostic biopsies (2), hypercalcemia, weight loss (history of disseminated coccidioidomycosis, breast cancer)	Unknown—presumptive lymphoproliferative disorder	+	Benign (negative)	Probable: High clinical suspicion of lymphoproliferative disorder +/− tuberculosis (working diagnosis)	+/EBV+	5+	0.32
PC43	Pleural	Liver masses, exudative pleural effusions, dyspnea, fatigue, weight loss	Unknown—presumptive hepatocellular carcinoma	+	Benign	Probable: High clinical suspicion	+	5+	0.31
PC44^b^	Pleural	Chest mass, pleural based lesions, dyspnea	Rhabdomyosarcoma	−	Benign (negative)	Tissue based UCSF500 testing: CNVs correlated	+	1	0.66
PC45	Pleural	Exudative effusion, lymphadenopathy (recent diagnosis of Hodgkin lymphoma, untreated), weight loss	Hodgkin lymphoma	+	Benign (negative)	Clinical suspicion, recent tissue diagnosis without treatment, CNV decrease after therapy, and EBV decrease after therapy	+/EBV+	4	0.35
PC46^b^	Pleural	Chronic pleural effusion requiring repeated drainage with known malignancy	Hodgkin lymphoma	−	Benign (negative)	Probable: High clinical suspicion	+	5+	0.26
PC47	Peritoneal	Known relapsed malignancy	Diffuse large B cell lymphoma	−	Benign (negative)	Probable: High clinical suspicion of known DLBCL with suggestive imaging	−	0	0.00
PC48	Peritoneal	Hepatic mass, ascites	Intrahepatic cholangiocarcinoma	+	Benign	Core Biopsy of liver: Adenocarcinoma	+	5+	0.31
PC49	Pleural	Pleural effusion, recently treated community acquired pneumonia, recurrent fever	Invasive ductal carcinoma	+	Benign	Breast core biopsy shows invasive ductal carcinoma	−	0	0.00
PC50	Pleural	Suspected malignant pleural effusion	Anal squamous cell carcinoma	−	Benign	Probable: High clinical suspicion for malignant effusion	+	5+	0.61
PC51	Pleural	Lung mass, exudative effusion	Unknown—suspected cancer	+	Benign	Probable: High clinical suspicion	−	0	0.00
PC52	Pleural	Known pulmonary metastasis	Colon adenocarcinoma	−	Benign	Probable: High clinical suspicion	+	5+	0.52
PC53	BAL	Lung mass, pancreatic mass, night sweats, weight loss	Melanoma	+	Benign	FNA: Melanoma	+	4	0.15
PC54	BAL	Lung mass, lymphadenopathy	Lung adenocarcinoma	+	Benign	FNA: Lung adenocarcinoma	+	5+	0.11
PC55	Peritoneal	Ascites	Breast cancer	−	Benign (negative)	Probable: High clinical suspicion	+	2	0.31
PC56	Pericardial	Effusions, fever, weakness	Castleman’s	+	Benign	Lymph node biopsy	−	0	0.00
PC57	Pleural	Effusions, fever, weakness	Castleman’s	+	Benign	Lymph node biopsy	−	0	0.00
PC58	BAL	Lung nodules, fever (known AML)	Acute myeloid leukemia	−	Benign	Cytogenetics (bone marrow) correlated with NGS CNVs	+^c^	5+	0.40
PC59	Peritoneal	Liver masses, ascites	Cholangiocarcinoma	−	Benign	FNA: adenocarcinoma	+	5+	0.55
PC60	Peritoneal	Liver masses, ascites	Hepatocellular carcinoma	−	Benign	FNA: Metastatic hepatocellular carcinoma	+	5+	0.55
PC61	Pleural	Liver masses, exudative pleural effusion, lung nodules	Colon cancer	−	Benign	Probable: High clinical suspicion	−	0	0.00
PC62	Peritoneal	Liver masses, ascites	Cholangiocarcinoma	−	Benign	FNA: adenocarcinoma	−	0	0.00
PC63^b^	BAL	Lung nodules, lymphadenopathy, non-diagnostic biopsies (5), eosinophilia, hypercalcemia	Myeloid neoplasm	+	Benign (negative)	Tissue FISH and cytogenetics (bone marrow) correlated with NGS CNVs: see Fig. 2	+	5+	0.96
PC64	FNA	Lymphadenopathy, splenomegaly, fever	Hodgkin lymphoma	+	Scant atypical lymphoid cells	Core Biopsy: Hodgkin lymphoma	−	0	0.00
PC65	BAL	Lymphadenopathy	Lung adenocarcinoma	+	Benign	FNA: adenocarcinoma	−	0	0.00

^a^New Dx (diagnosis): either no history of the cancer or no treatment of a newly diagnosed cancer

^b^Body fluid is correlated with cancer tissue (see Fig. 2G, H for PC63 and Additional file 1 for all other cases)

^c^Plasma NGS (same protocol) 1 day prior shows a lower tumor fraction at 27% (versus 40% in the BAL)

To evaluate test specificity, we ran the CNV test on 59 body fluids from acutely ill hospitalized patients with microbiologically proven (culture, serology, antigen, PCR) infection but without evidence of malignancy (Additional file 3: Table S2, Additional file 4: Table S3). All 59 fluids were all negative for detection of CNVs, placing the estimated specificity at 94–100% (95% CI, Clopper-Pearson).

### Example: PC63

**Fig. 2:**
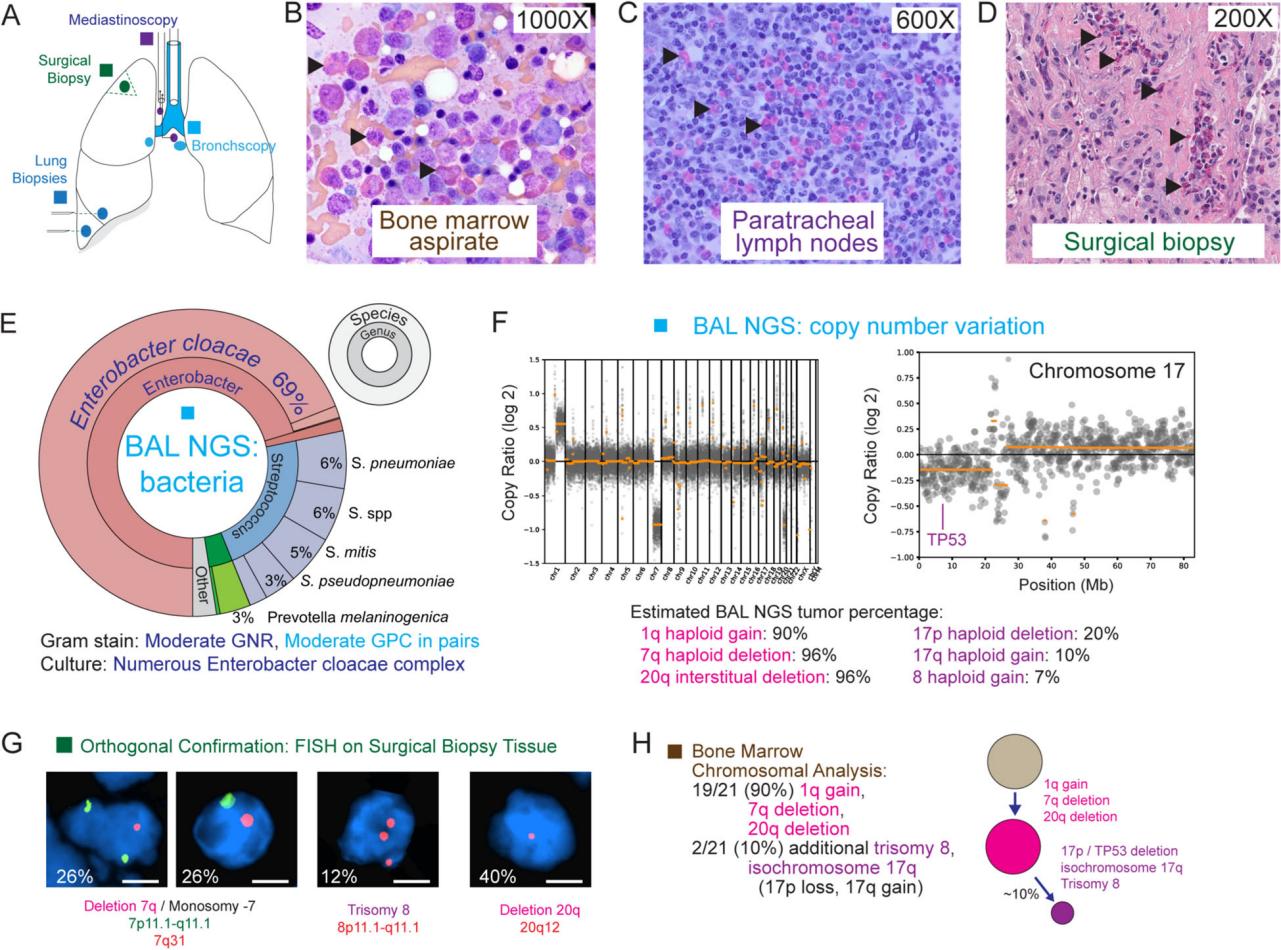
Patient PC63 showing biopsies, mNGS pathogen/CNV results, and orthogonal confirmation. **A** Schematic of the biopsies performed. **B**–**D** Histology of the bone marrow, paratracheal lymph node, and lung wedge biopsy show increased eosinophils (arrowheads) that were morphologically normal and indistinguishable from reactive and benign eosinophils. **E** Bacterial profile from mNGS testing. No viral, fungal, or parasitic pathogens were detected. **F** Copy number plotting across the human genome derived from metagenomic sequencing data. Six chromosomal scale deletions and duplications are identified, 3 of which accounted for > 90% of the human DNA content. **G** FISH (fluorescence in situ hybridization) of wedge biopsy, confirming presence of matching clonal complex cytogenetics to BAL fluid in **F**. Scale bar, 5 μm. **H** Bone marrow biopsy, confirming presence of matching clonal complex cytogenetics to BAL fluid in **F**

An adult patient presented with fever, dyspnea, weakness, and weight loss and was found to have eosinophilia and a > 3-cm lung mass. The patient underwent several non-diagnostic thoracic procedures, including bronchoscopies, mediastinoscopy, thoracentesis, and a surgical biopsy (Fig. 2A). The patient’s bone marrow biopsy revealed increased eosinophils and precursors, and chronic eosinophilic leukemia (CEL) was suspected based on an abnormal karyotype [23]. CEL is a rare entity with diagnostic criteria that include (i) eosinophilia (eosinophil count ≥ 1.5 × 10^9^/L) (criteria not met) and (ii) clonal cytogenetic or molecular genetic abnormality or increase in BM or peripheral blood blasts (criteria met). However, her lung disease was unexplained as eosinophils detected in the thoracic biopsies did not appear dysplastic morphologically (Fig. 2B–D). It was uncertain whether the eosinophils were reactive secondary to pulmonary infection and/or inflammation or myeloid neoplasm.

The bronchoalveolar lavage (BAL) fluid underwent mNGS. Bacteria, but not fungi, viruses, and parasites, were detected by mNGS, and the bacterial profile, consisting predominantly of reads from *Enterobacter cloacae*, matched Gram stain and culture results from the BAL fluid (Fig. 2E). However, this bacterial infection was not considered as the underlying cause for the patient’s initial clinical presentation nor her ongoing pulmonary symptoms. The CNV analysis showed gains in chromosome 1q, 8, and 17q and losses in 7q, 17p, and 20q and indicated that this clonal process comprised up to 94% (range 90–96%) of the total DNA (Fig. 2F). Fluorescent in situ hybridization (FISH) analysis of resected lung tissue confirmed the same cytogenetic abnormalities (Fig. 2G). The CNV and cytogenetic profile found in BAL fluid and lung tissue matched the clone found in the patient’s bone marrow biopsy (Fig. 2H), implicating leukemic infiltration of the lung as the most likely cause of the patient’s lung mass and acute illness.

### Second verification cohort

To further verify our findings, we performed a secondary verification study at a separate medical site (Stanford Medical Center), comprised of 81 consecutive cases (56% pleural, 19% peritoneal, 14% bronchoalveolar lavage, 4% pericardial, 2% fine needle aspirate). These were available residual samples used for testing by cytology from a single laboratory, and no available samples with sufficient volume were excluded.

Using the criteria in the test performance study, there were 32 total positive cases. The sensitivity of the 27 cases that were positive by cytology or flow cytometry was 89% (95% CI 71–98%), with a median tumor fraction of 34% (IQR 14–46%). Of the 5 positive cases that were negative by cytology, 4 were detectable by NGS. The specificity of all 18 cases with no cancer diagnosis and an alternative diagnosis made by the treating physician was 100% (95% CI 81–100%).

The remaining cases (n = 22) that did not match the inclusion criteria for positives and negatives were composed of patients with an unclear gold standard. These cases either had an actively treated cancer or did not have at least a working diagnosis that prompted treatment. Six of the 22 cases (27%) were positive. Of the 9 cases with no history of cancer and had an unclear diagnosis, one was positive.

Across the two studies, the overall sensitivity was 87% (95% CI 77–94%) for cytology/cytometry-positive cases and 68% (95% CI 49–83%) for cytology/cytometry-negative cases but were ultimately diagnosed with an adjacent malignancy (Fig. 1C, D). The overall specificity using only negative controls was 100% (95% CI 95–100%).

### Microbial analysis

We performed three microbiological evaluations of the current data. First, we evaluated all positive cancer cases for oncoviruses. In three of the 97 cancer-positive cases (65 from test performance study and 32 from the verification cohort), Epstein-Barr virus (EBV)/human herpesvirus 4 (HHV4), a gammaherpesvirus human oncovirus [24], was detected by mNGS. The cases were angioimmunoblastic T cell lymphoma (P13), Hodgkin lymphomas (P45), and a presumptive lymphoproliferative disorder, otherwise not classified (P42). In one case, CNV detection alone was negative (PC13). In 2 of 3 EBV positive cases with sufficient EBV reads for further characterization, both had cfDNA length distributions consistent with oncovirus integration into the human genome (as opposed to EBV reactivation), based on criteria previously reported for cfDNA from EBV-positive nasopharyngeal carcinoma [25] (Additional file 1). Alphapapillomavirus 9, which includes human papillomavirus (HPV) type 16, was positive in two cases (PC50, 3134) related to the patient’s squamous cell carcinoma of the anus and vulva, the latter of which was known to express HPV p16 on immunohistochemistry.

The performance characteristics for microbial detection of the first study were reported previously [20]. However, in the second analysis, we found 10 cases (11 microbial pathogens) across all cases in the first study that had a gold standard pathogen as previously reported and with non-specific clinical presentations that could be associated with infection or cancer (e.g., fever, lymphadenopathy, weight loss, mass) (Additional file 3: Table S2). When assessing the positive gold standard organisms, all but one had more organisms than all other samples in the first study (Additional file 6: Figure S1).

In the third analysis, we analyzed all new cases in the second cohort without a clear diagnosis prior to the NGS result (n = 9) and found 2 significant pathogens based on past criteria [20]. Case 3026 was a transplant patient with a B cell deficiency and a remote cancer history who presented with hemoptysis and was found to have pulmonary consolidations and eosinophilia. Microbial analysis showed that *Haemophilus influenzae* as a significant occult pathogen at 1412 species-specific reads, and all reads compatible with H. *influenzae* declassified up to the taxonomic Family level accounted for 95% of all of the bacterial and fungi reads. The patient had a history of *H. influenzae* infection and previously received amoxicillin for presumed pneumonia, which may not have been an adequate treatment initially. The patient improved under empiric therapy that included a third-generation cephalosporin. In our experience with metagenomic NGS, *H. influenzae* is an organism often missed by conventional methods [20, 26, 27]. Anelloviruses were also found, consistent with the patient’s known immunocompromised status. Case 3095 was a transplant patient with bilateral pleural effusions of uncertain etiology that was attributed to acute respiratory distress syndrome (ARDS). Microbial analysis showed 7434 EBV reads and degraded human DNA precluded analysis for oncovirus integration. The patient was known to be immunocompromised with a low level of EBV viremia in the past year.

## Discussion

In this study, we show that residual data from metagenomic and whole genome sequencing can provide reliable CNV data and detect 68% (23 of 34) of malignant body fluids when they were undetectable through conventional testing provided by cytology and flow cytometry. Detection of missed cases highlights the potential of sequencing-based tests in finding malignancies earlier or less invasively in cases without a clear diagnosis. Surprisingly, these NGS-positive body fluids were high in tumor fraction (median 32%, IQR 27–58%) despite negative conventional testing by cytology and flow cytometry. These cases, including the case PC63, underscore the challenges in the diagnosis of malignancy or infection in acutely ill patients who have overlapping clinical presentations. Both conditions can present with B-symptoms (fever [28], night sweats, weight loss), lymphadenopathy, eosinophilia [29], exudative effusions [9], and nodules/masses/cavitations [30]. Notably, over 25% of pulmonary nodule biopsies are non-diagnostic and 21% of those had a final diagnosis that was malignant [30]. Across both studies, whole genome testing detected 7 of 10 of such pulmonary nodule/masses in cases not found by conventional testing. As another example, 25% of cryptogenic hepatocellular carcinoma had ascites as part of their presentation [31] whereas few are positive based on traditional testing including cytology [11]. Whole genome testing detected 4 of 5 (80%) of such liver mass cases not found by conventional testing.

Here we demonstrate dual use of metagenomic sequencing of cfDNA in body fluids to simultaneously screen for infection and cryptogenic malignancy. Previous groups have detected incidental malignancies in pregnant women by non-invasive prenatal testing (NIPT) of blood [32]. However, the incidence of malignancy in asymptomatic pregnant women is ~ 0.1%, which is low compared to the 20–25% incidence in hospitalized patients with non-specific acute illness [30, 31]. We and other groups have also previously demonstrated the presence of tumor cfDNA in body fluids [33–36], but these studies have not focused on broad-based screening for cryptogenic malignancies nor the potential repurposing of metagenomic data used for pathogen identification for cancer diagnosis.

The advantages of CNV body fluid testing to screen for malignancies include (i) leveraging of clinical mNGS data already generated for infectious disease diagnosis [1–3], (ii) rapid turnaround time (< 48 h) that is crucial for critically ill patients, (iii) straightforward interpretation compared to cancer gene panel testing [37], (iv) increase in diagnostic yield over conventional testing (detection of 66% of cases not found by conventional testing), and (v) high analytical specificity (no false positives out of 59 samples). High specificity was also illustrated in 3 large NIPT studies [13–15] involving 124,000, 450,000, and 1.93 million patients, where the frequency of CNV abnormal cases (multiple aneuploidies) in plasma was only 0.031%, 0.012%, and 0.033%, with confirmation of maternal cancer in 18%, 47%, and 7.6% of those positives respectively. Another advantage is the addition of cytogenetic and viral (e.g., EBV) driver characterization of the tumor to facilitate diagnosis, provide prognostic information, and potentially guide targeted therapy (Table 1, Additional file 2: Table S1). Finally, body fluids are often available in ample quantities and are both easier and less invasive to collect than tissue biopsies. The CNV test presented here uses only 0.4 mL of body fluid input and can be performed on discarded supernatant byproducts of traditional cell-based assays such as cytology, flow cytometry, or microbiological culture.

Limitations of this testing approach include the lack of CNVs in a minority (< 10%) of malignant neoplasms even though > 90% of solid tumors have CNVs [19] and the analytical requirement for approximately > 5% tumor fraction, similar to NIPT [38]. Although cancer gene panels are capable of detection at lower tumor fractions, often down to 1% [37], there is potential concern for false-positive results of low burden pathogenic mutations that can be incidentally detected in normal controls [39–42] and benign growths [43, 44]. Using subsequent targeted gene panels is not ruled out by this testing approach, but rather informed by the rapid assessment for positive cancer samples, which can also have higher tumor fractions than tissue biopsies (e.g., PC46, Additional file 1). In the current study, the median tumor fractions in laboratory-confirmed and unconfirmed cancer samples were 43% and 26%, respectively, well above the minimum threshold.

## Conclusions

The dual ability to screen for cryptogenic malignancies and pathogens by metagenomic whole genome sequencing of body fluids simultaneously on the same patient specimen may reduce time to diagnosis and increase diagnostic accuracy. Early diagnosis of malignancy and/or infection may enable further workup and guide more timely treatment, while the availability of high tumor fraction material in the body fluids allows for further molecular testing to classify the cancer and find actionable driver mutations (e.g., KIT [45] in the index case). Clinical validation and prospective diagnostic trials will be needed to investigate the clinical utility and ethical ramifications of this test for simultaneous cancer diagnosis and pathogen detection.

## Supplementary Information

**Additional file 1: Supplementary Results**.

**Additional file 2: Table S1**. Body fluid samples from patients positive for malignancy by cytology and/or flow cytometry.

**Additional file 3: Table S2**. Select microbiological cases that have overlapping features with cancer presentations.

**Additional file 4: Table S3**. Microbiological cases - all other cases.

**Additional file 5: Table S4**. Verification cohort.

**Additional file 6: Figure S1**. Microbiological cases with overlapping features with cancer presentations.

## Data Availability

CNVkit (https://cnvkit.readthedocs.io/) [17] and SURPI+ v.1.0 (https://github.com/chiulab/SURPI-plus-dist) software [18] for CNV and pathogen detection are both available for free online. CNV data, Metagenomic Fastq data, image data, and data analysis scripts were deposited or linked to on Zenodo (10.5281/zenodo.4697549) [46]. The CNV datasets can be read with a text editor or CNVkit [17]. Metagenomic sequencing data (FASTQ files) with human genomic reads removed were also deposited as a NCBI SRA under Bioproject PRJNA707099, https://www.ncbi.nlm.nih.gov/bioproject/?term=PRJNA707099 [47].

## Abbreviations

FNA: Fine needle aspirate
EBUS: Endobronchial ultrasound
EBV: Epstein-Barr virus
BAL: Bronchoalveolar lavage
CNV: Copy number variation
DLBCL: Diffuse large B cell lymphoma
AML: Acute myeloid leukemia
FISH: Fluorescence in situ hybridization
NGS: Next-generation sequencing
FoundationONE/UCSF500: Cancer panels based on NGS
