# Supplementary material for: Detection of cryptogenic malignancies from metagenomic whole genome sequencing of body fluids
Source: Genome Med. 2021 Jun 1;13:98. doi: 10.1186/s13073-021-00912-z (PMC8167833; doi:10.1186/s13073-021-00912-z)
Supplement: Supplementary file 1 — Additional file 1: Supplementary Results. [file 13073_2021_912_MOESM1_ESM.docx]

**Supplementary Results**

**Detection of Cryptogenic Malignancies from Metagenomic Whole Genome Sequencing of Body Fluids**

Wei Gu^1,2,3,4^*, Eric Talevich^5^, Elaine Hsu^1^, Zhongxia Qi^1^, Anatoly Urisman^6^, Scot Federman^1,2^, Allan Gopez^1,2^, Shaun Arevalo^1,2^, Marc Gottschall^1^, Linda Liao^4^, Jack Tung^3^, Lei Chen^4^, Harumi Lim^4^, Chandler Ho^4^, Maya Kasowski^3^, Jean Oak^3,4^, Brittany Holmes^3,4^, Iwei Yeh^6^, Jingwei Yu^1^, Linlin Wang^1^, Steve Miller^1,2^, Joseph DeRisi^7,8^, Sonam Prakash^1^, Jeff Simko^6,^ ^§^ , and Charles Chiu^1,2,9,^ ^§^ *

^1^Department of Laboratory Medicine, University of California San Francisco, CA 94107, USA

^2^UCSF-Abbott Viral Diagnostics and Discovery Center, San Francisco, CA 91407, USA

^3^Department of Pathology, Stanford University, CA 94305, USA

^4^Stanford Health Care, Stanford University, CA 94305, USA

^5^DNANexus, Mountain View, CA 94040, USA

^6^Department of Pathology, University of California San Francisco, CA 94107, USA

^7^Department of Biochemistry and Biophysics, University of California San Francisco, CA 94107, USA

^8^Chan Zuckerberg Biohub, San Francisco, CA 94107, USA

^9^Department of Medicine, Division of Infectious Diseases, University of California San Francisco, CA 94107, USA

^§^These authors contributed equally: Charles Chiu, Jeff Simko

* Corresponding authors. Email: weigu@stanford.edu, charles.chiu@ucsf.edu

**Supplementary Results - Table of Contents**

Body fluid correlation with cancer tissue

Example of Negative CNV Results

Read Depth Analysis

EBV Length Distributions

**Body fluid correlation with cancer tissue**

| ID | Pearson correlation |
| --- | --- |
| PC1 | 0.966684872 |
| PC2 | 0.427046753 |
| PC3 | 0.50901895 |
| PC38 | 0.557296819 |
| PC39 | 0.756523035 |
| PC7 | 0.3737086 |
| PC14 | 0.864863224 |
| PC15 | -0.004801686 |
| PC41 | -0.129946645 |
| PC46 | 0.610734373 |

For each tissue-body fluid paired case, the Pearson correlation of gene-level copy ratio estimates was calculated. Correlation was strong where significant aneuploidy was present, up to a maximum correlation coefficient of .97. In the two cases where no aneuploidy was present, there was no correlation, as would be expected. Across all 10 cases, regardless of the degree of aneuploidy in each sample, the median Pearson correlation was 0.53. The discrepancies can partly be explained by tumor evolution, as the two samples in each case were collected at different time points, sometimes years apart.

Sample PC1

| Pleural Fluid  NGS | 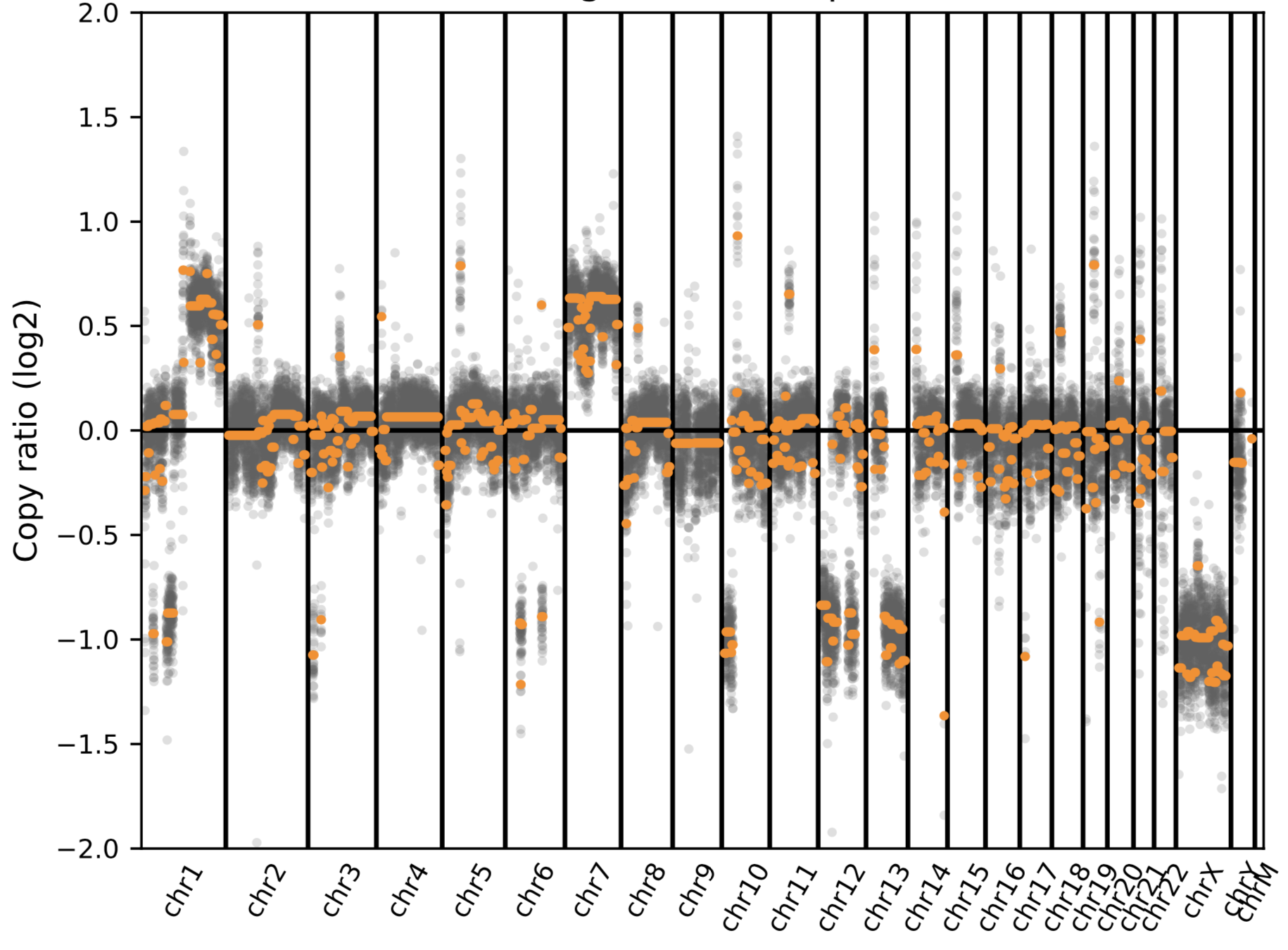 |
| --- | --- |
| Cancer Tissue | 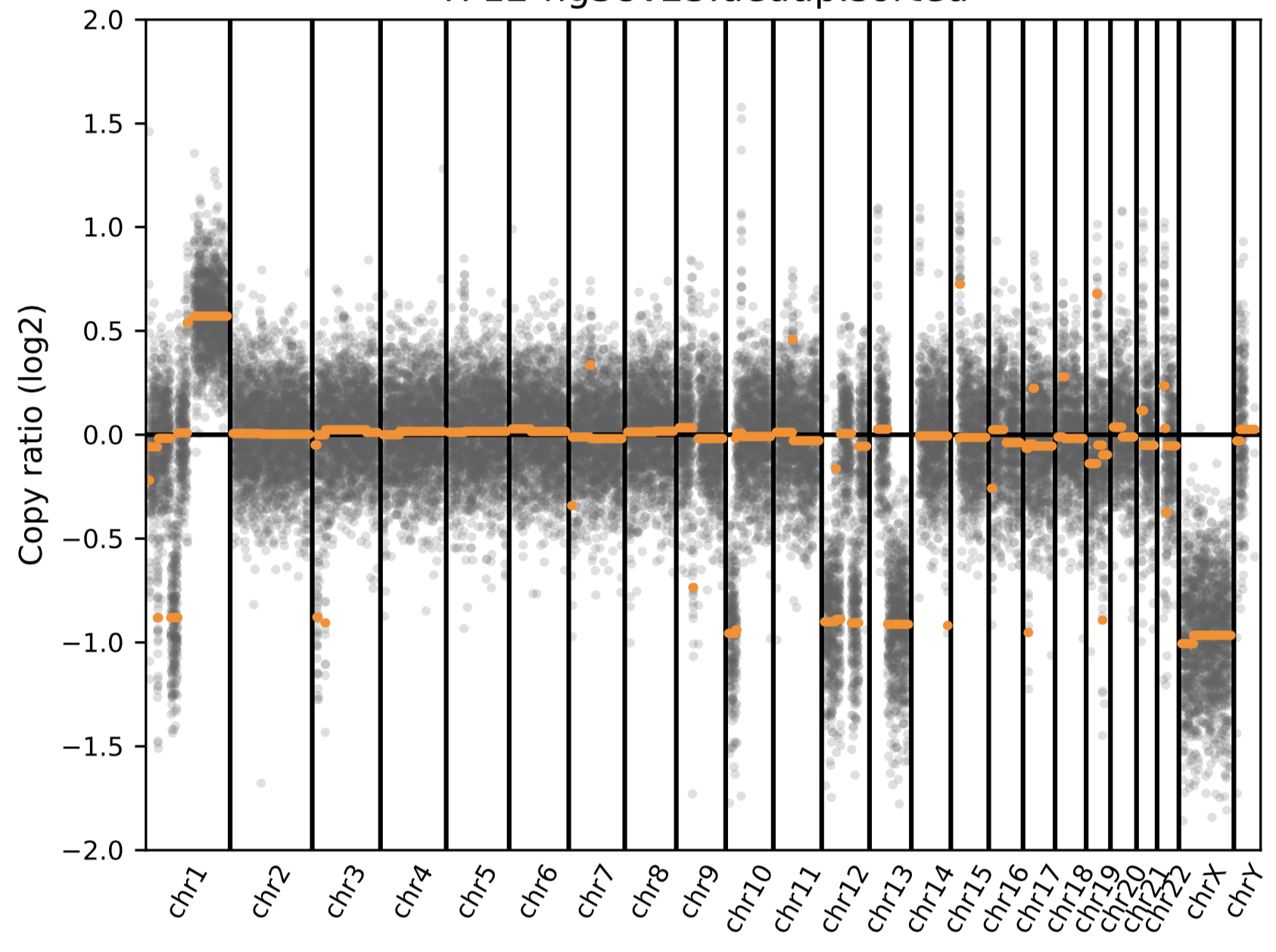 |

Sample PC2

| Peritoneal Fluid  NGS | 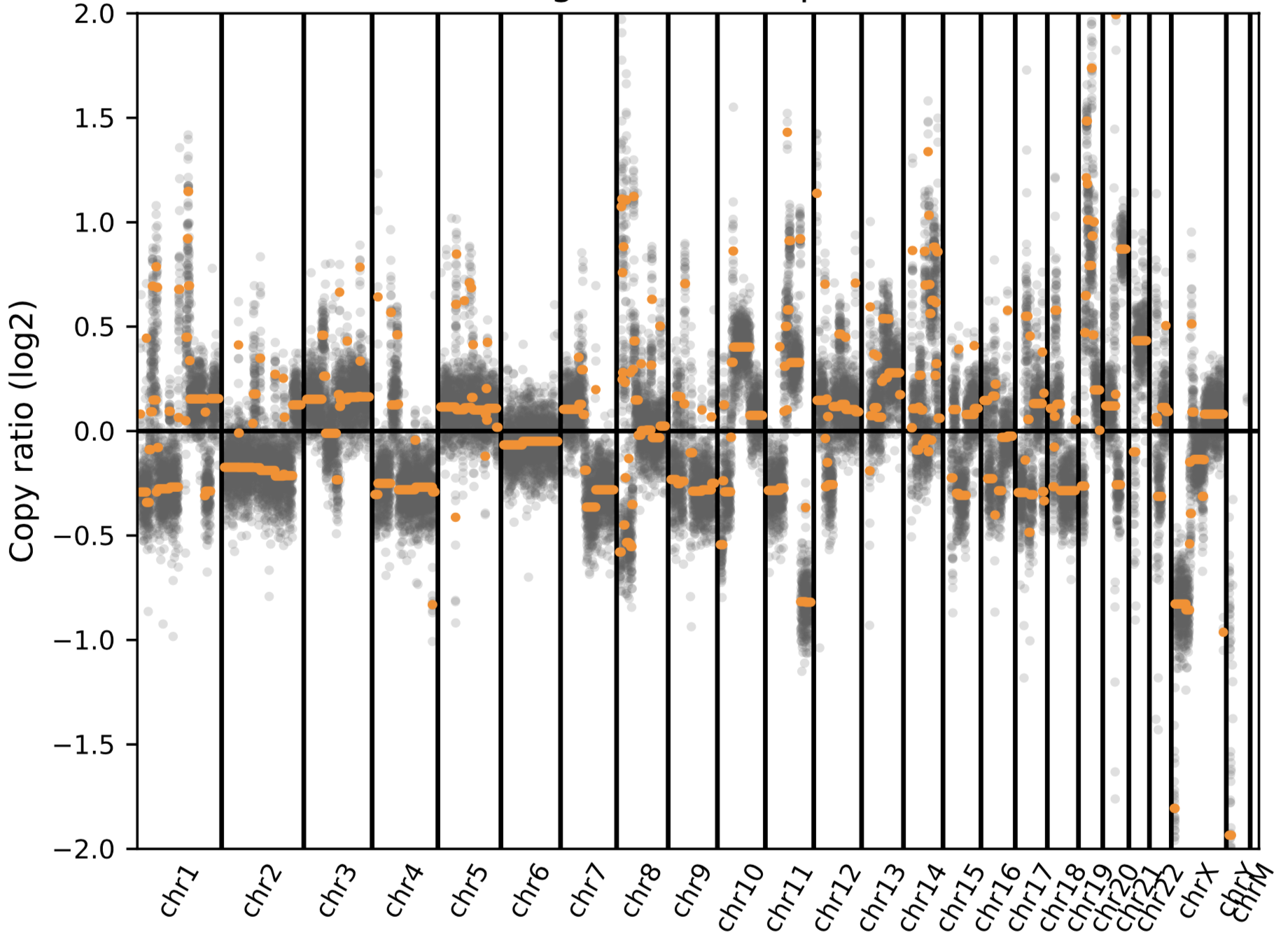 |
| --- | --- |
| Cancer Tissue | 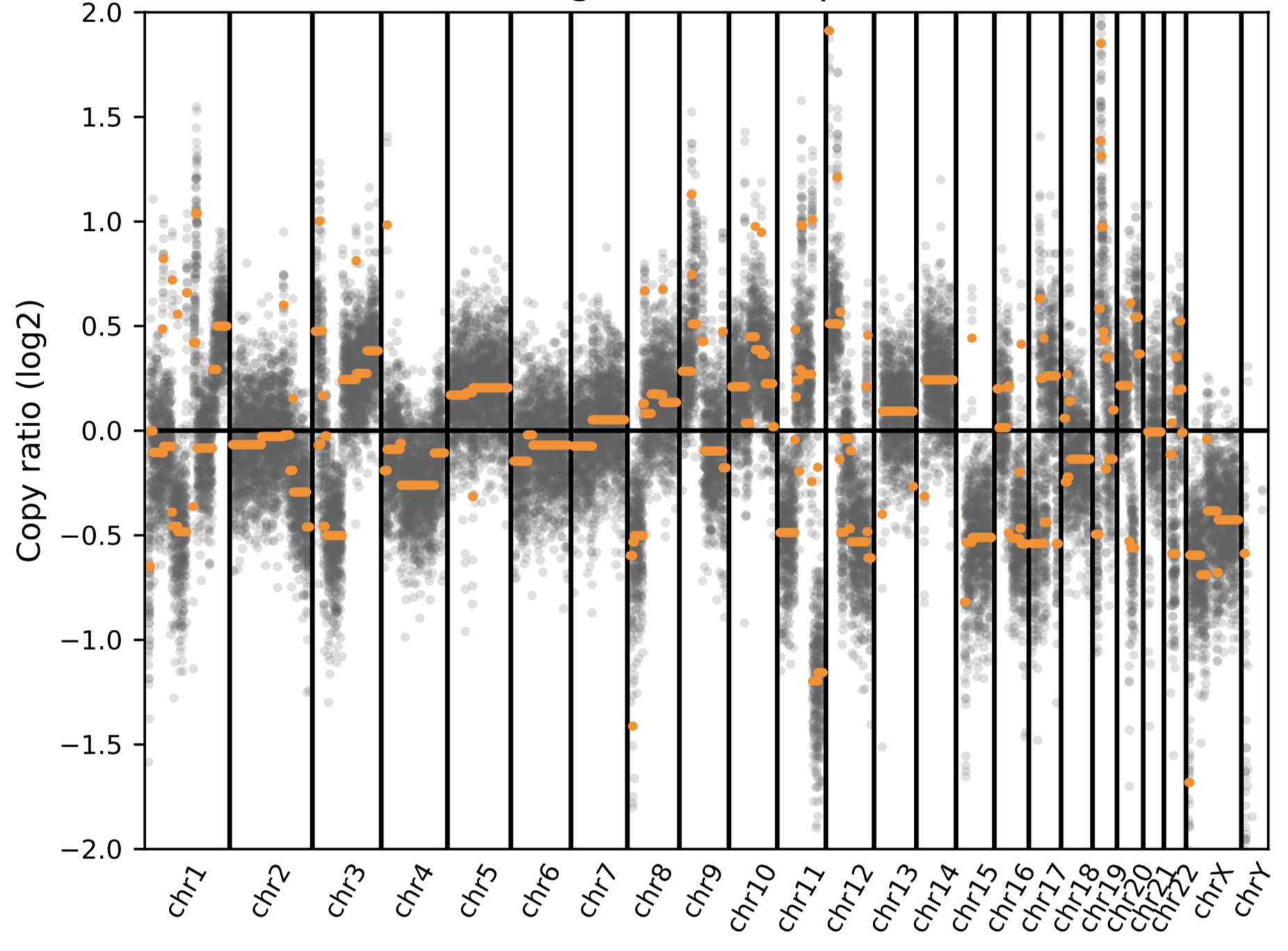 |

Sample PC3

| Pleural Fluid  NGS | 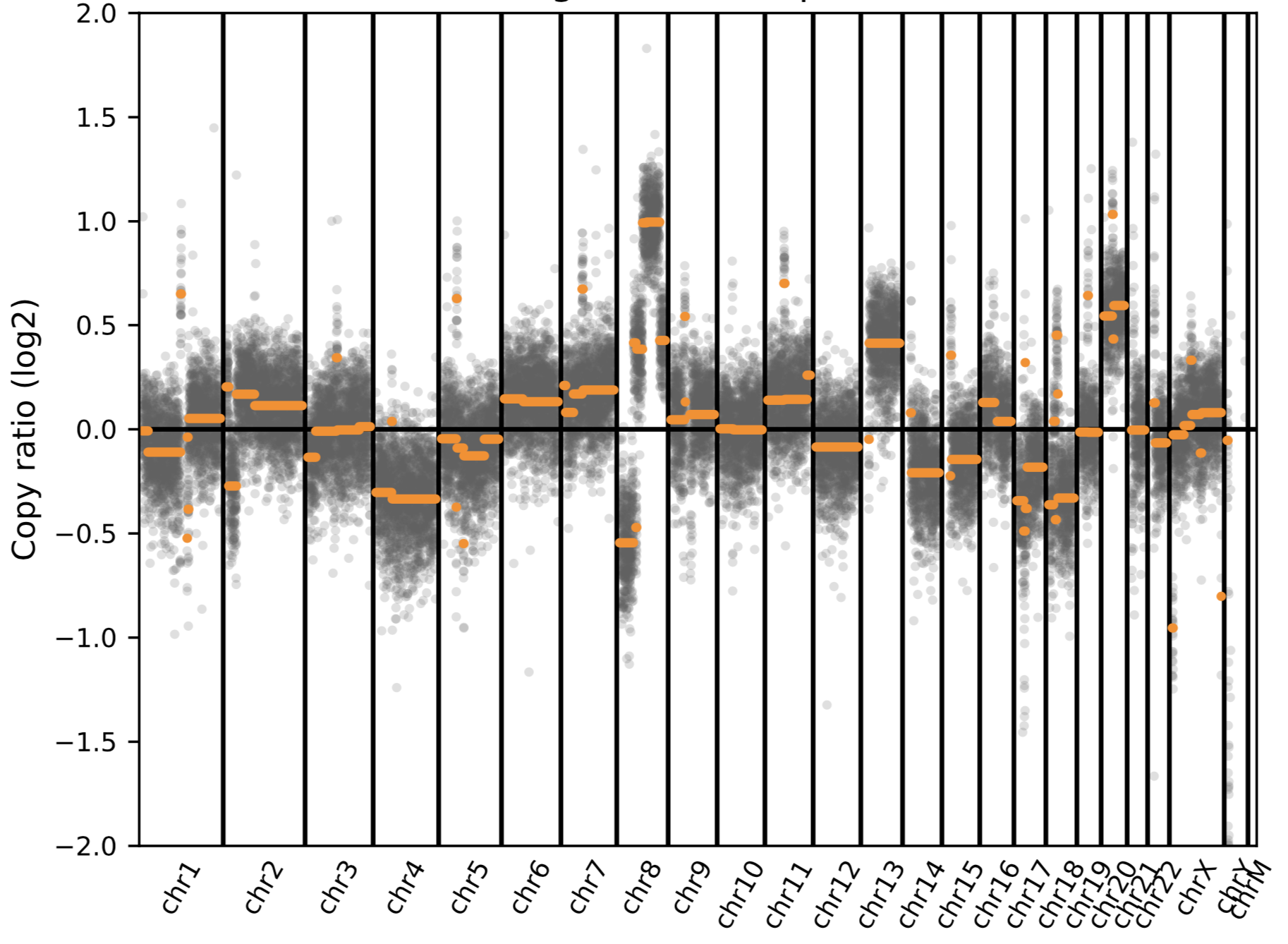 |
| --- | --- |
| Cancer Tissue | 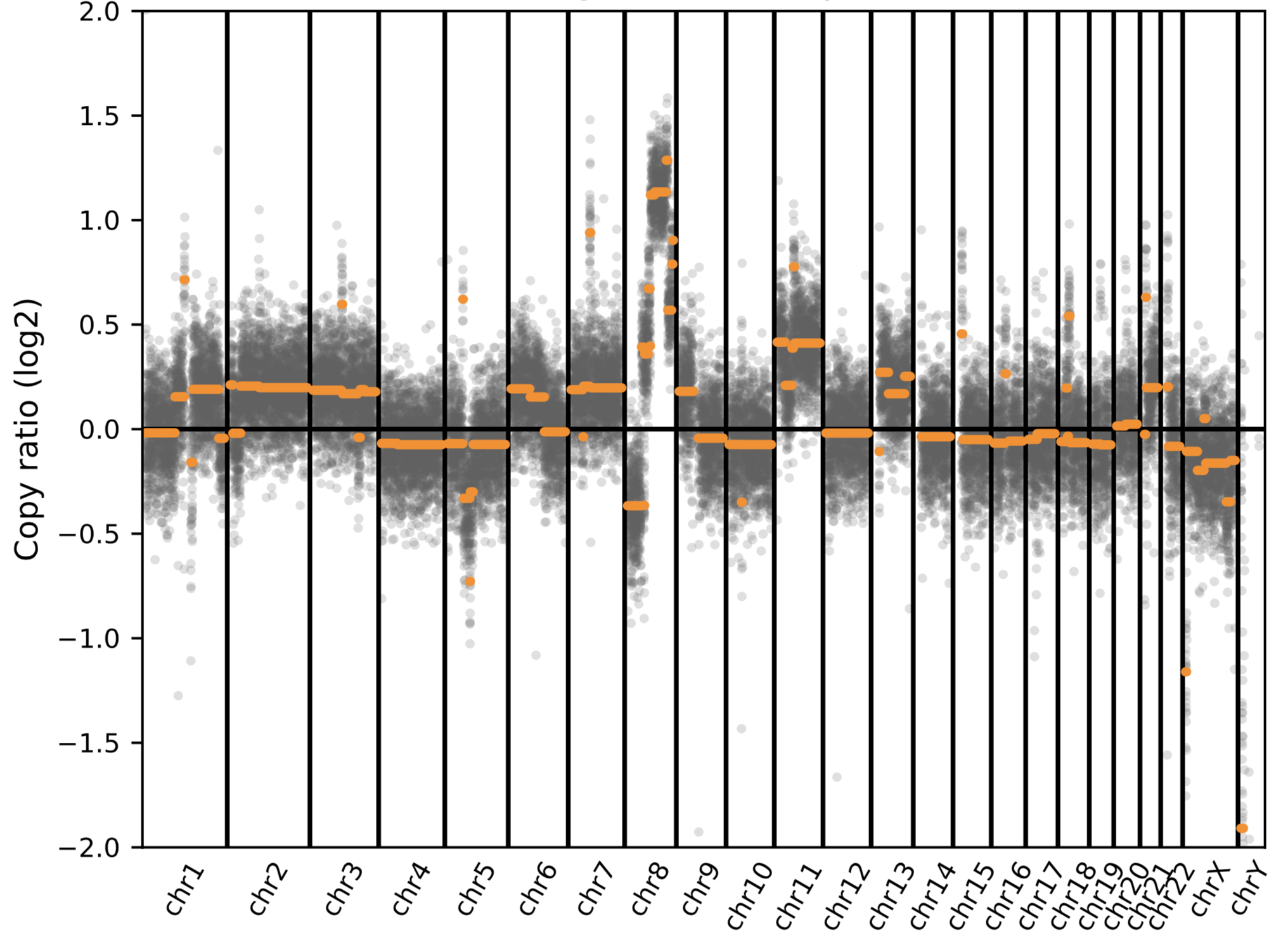 |

Sample PC7: This follicular lymphoma (grade 3a) was called negative for CNVs in the pleural fluid and missed by NGS, whereas it was positive for CNVs in the tissue. Therefore, the false negative by NGS is presumably due to a low tumor fraction in the pleural fluid.

| Pleural Fluid  NGS | 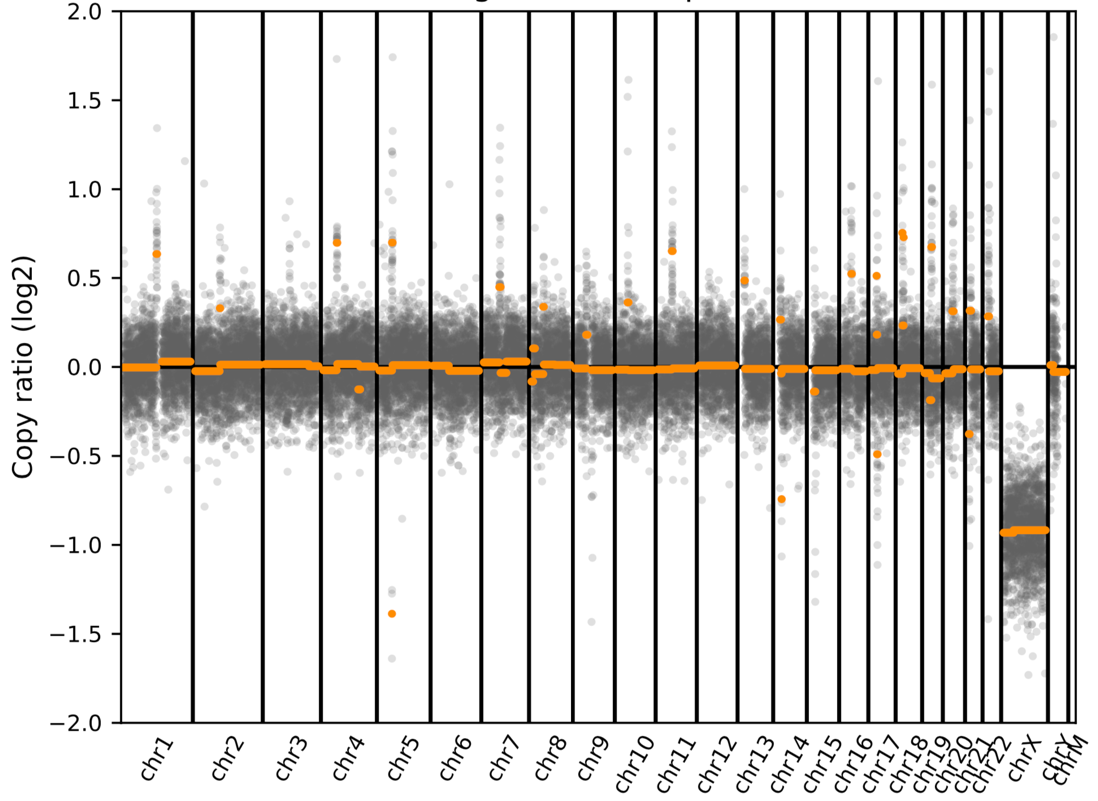 |
| --- | --- |
| Cancer Tissue | 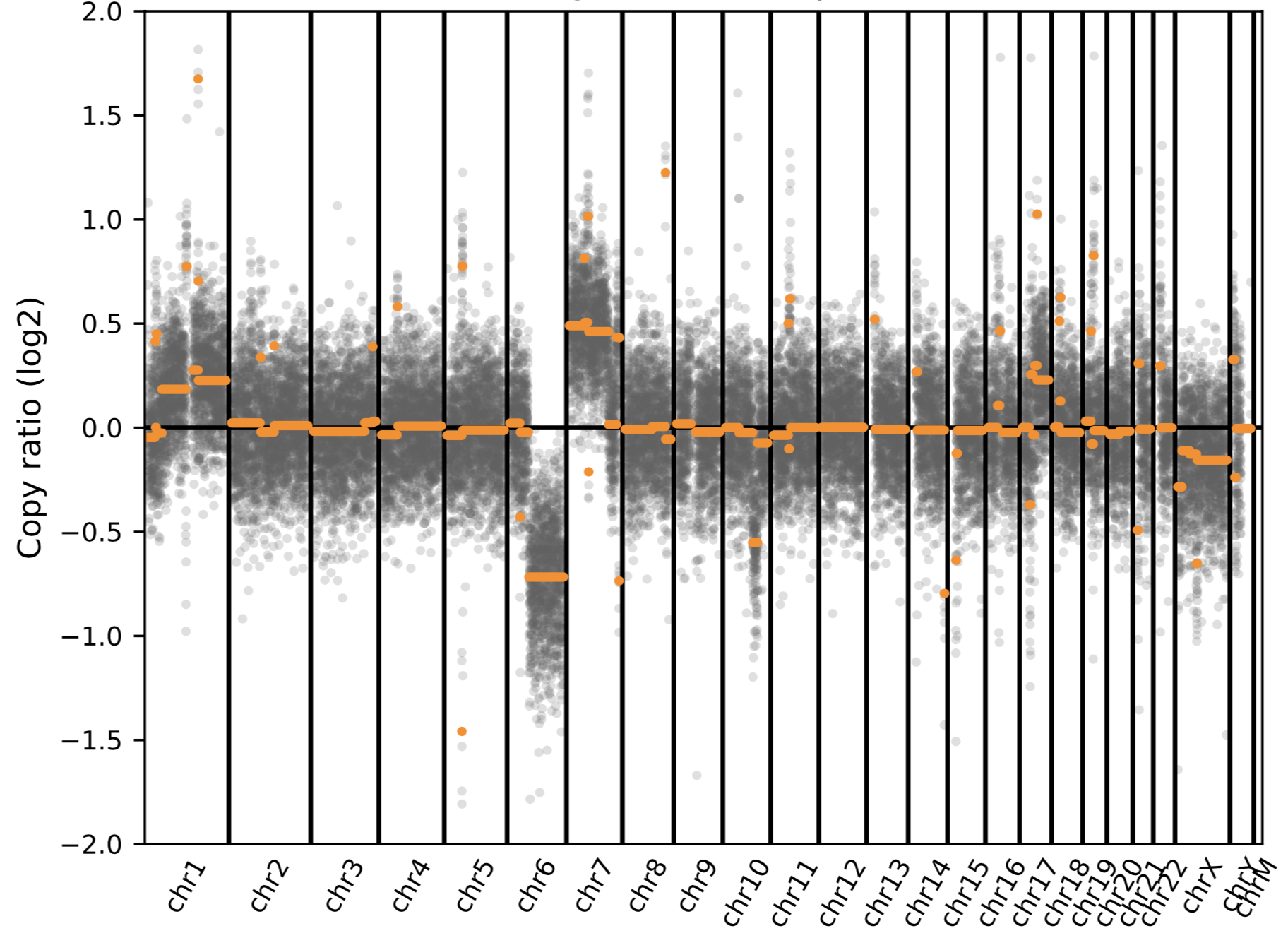 |

Sample PC8

Peritoneal Fluid NGS:
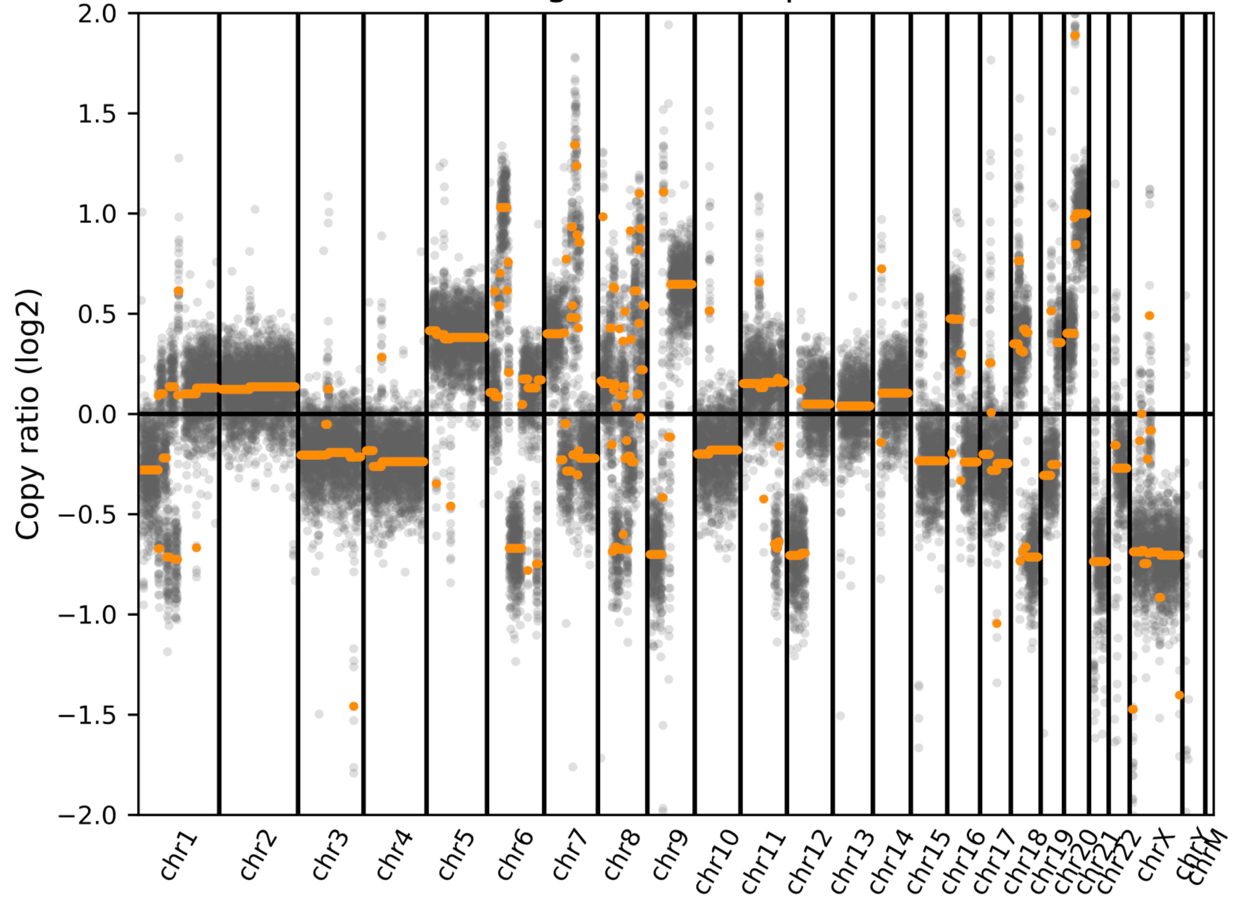


Cancer Panel on Cancer Tissue (Foundation One): CDK6 (7q), EPHB4 (7q) amplifications.

Sample PC14

| Pleural Fluid  NGS | 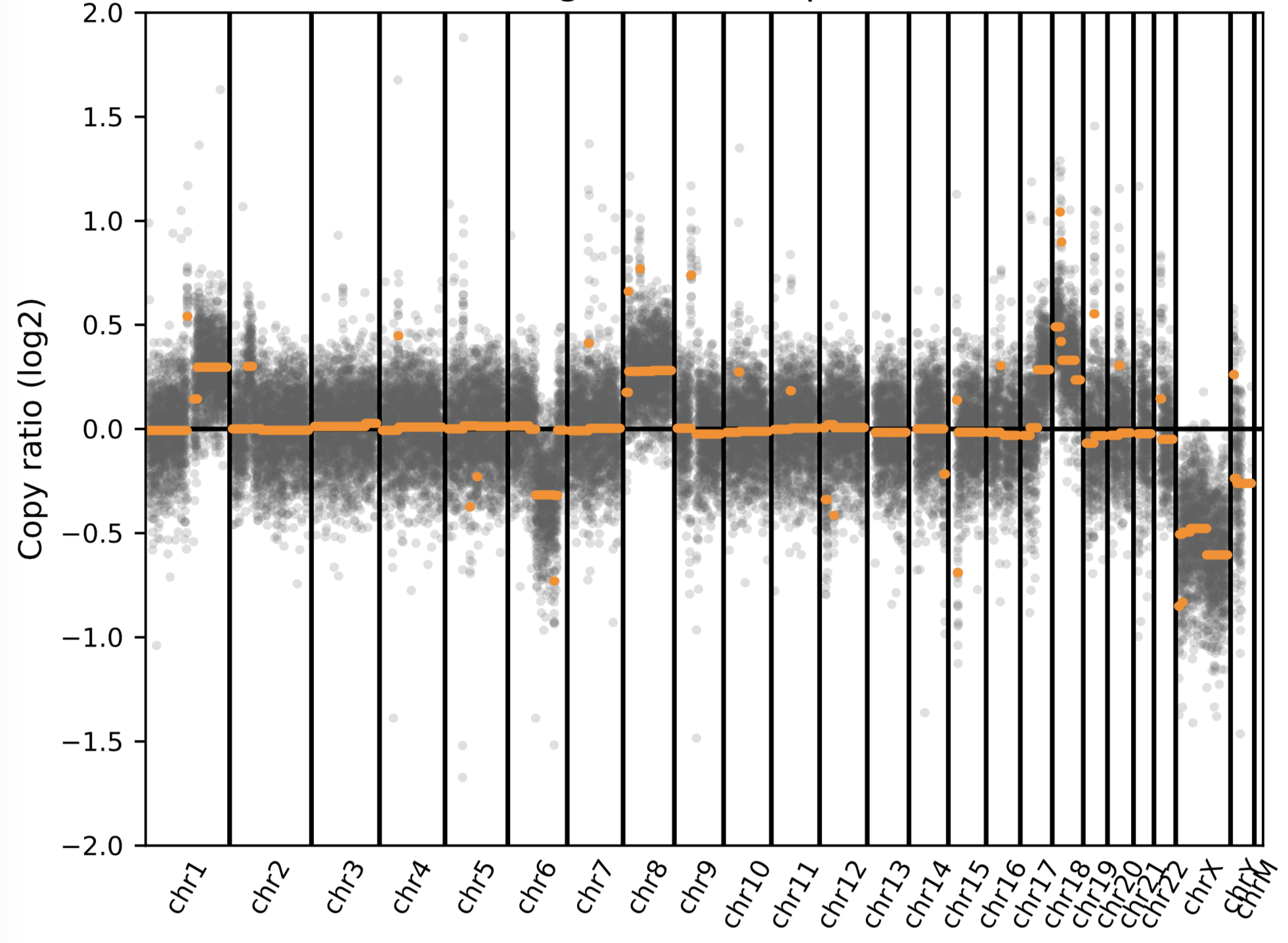 |
| --- | --- |
| Cancer Tissue | 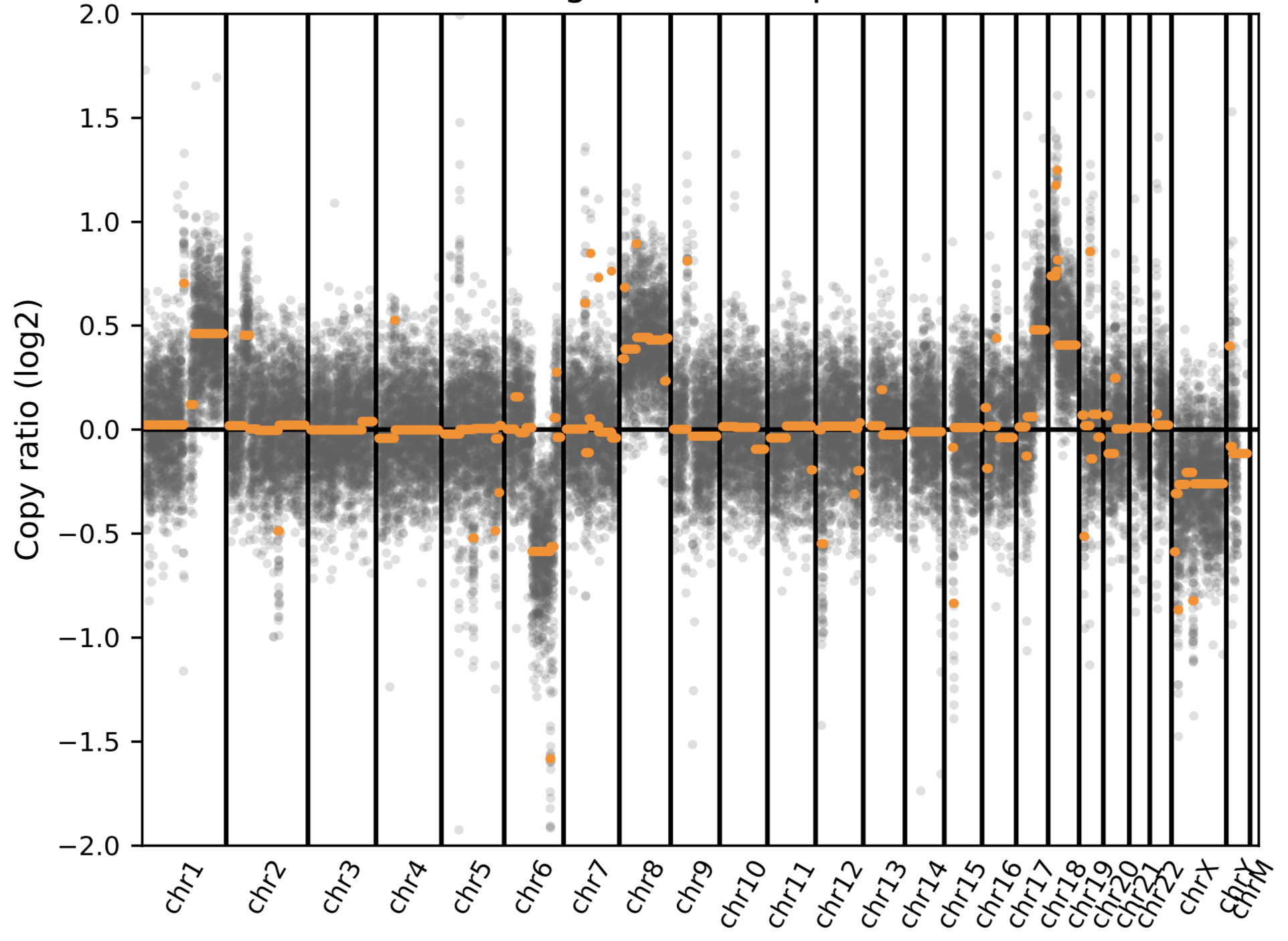 |

Sample PC15

| Pleural Fluid  NGS | 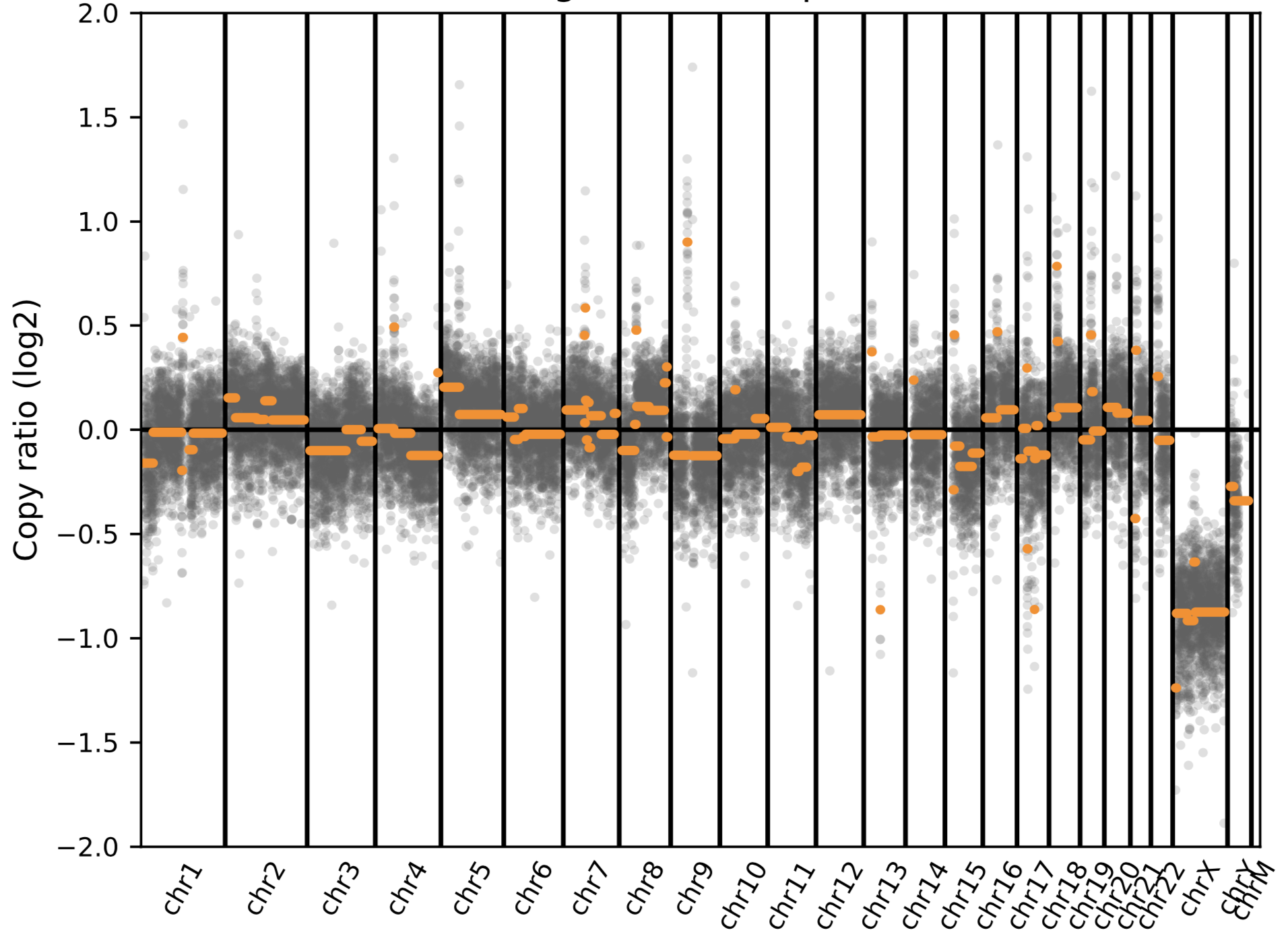 |
| --- | --- |
| Cancer Tissue | 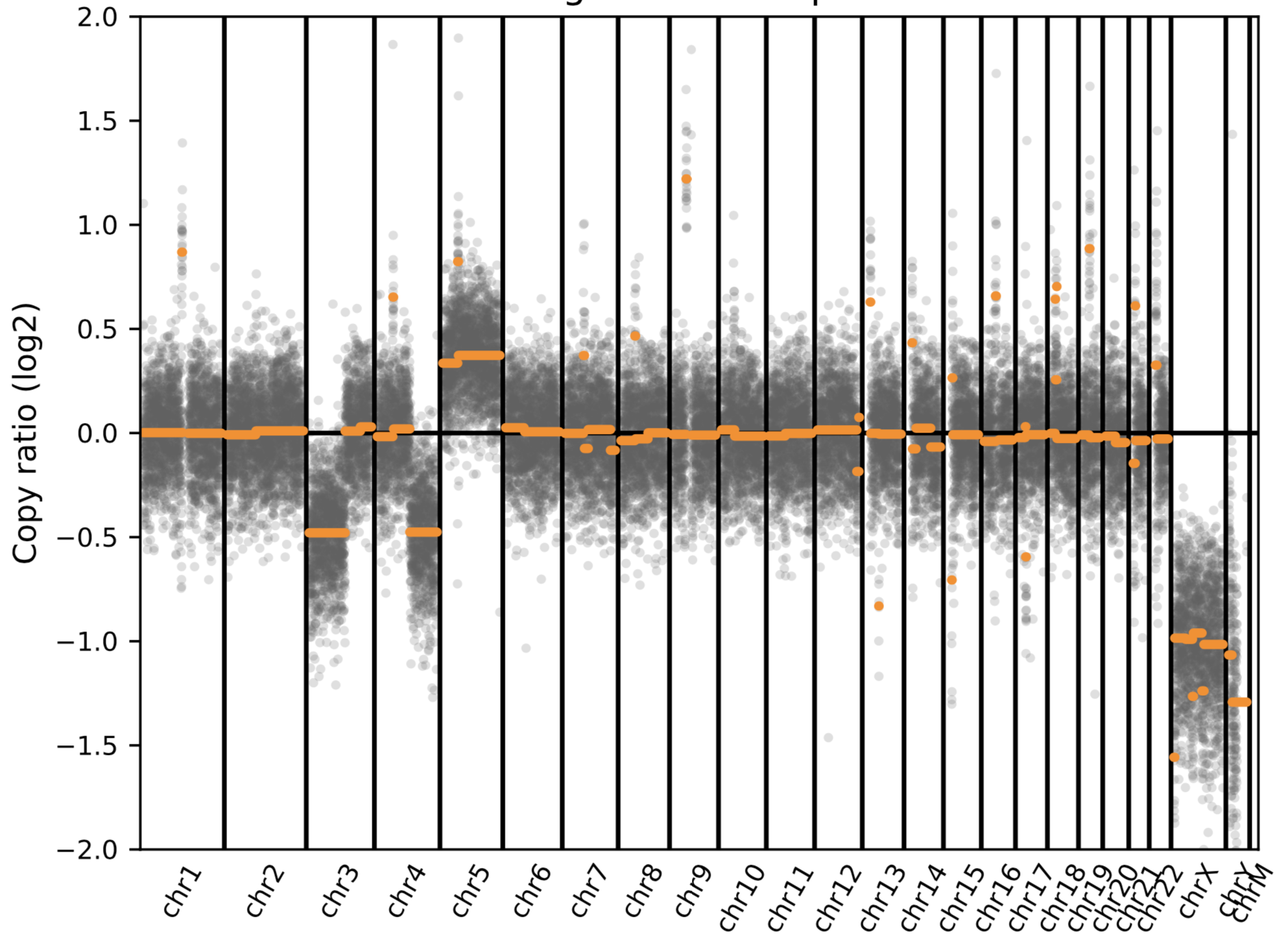 |

Sample PC22

| Pleural Fluid  NGS | 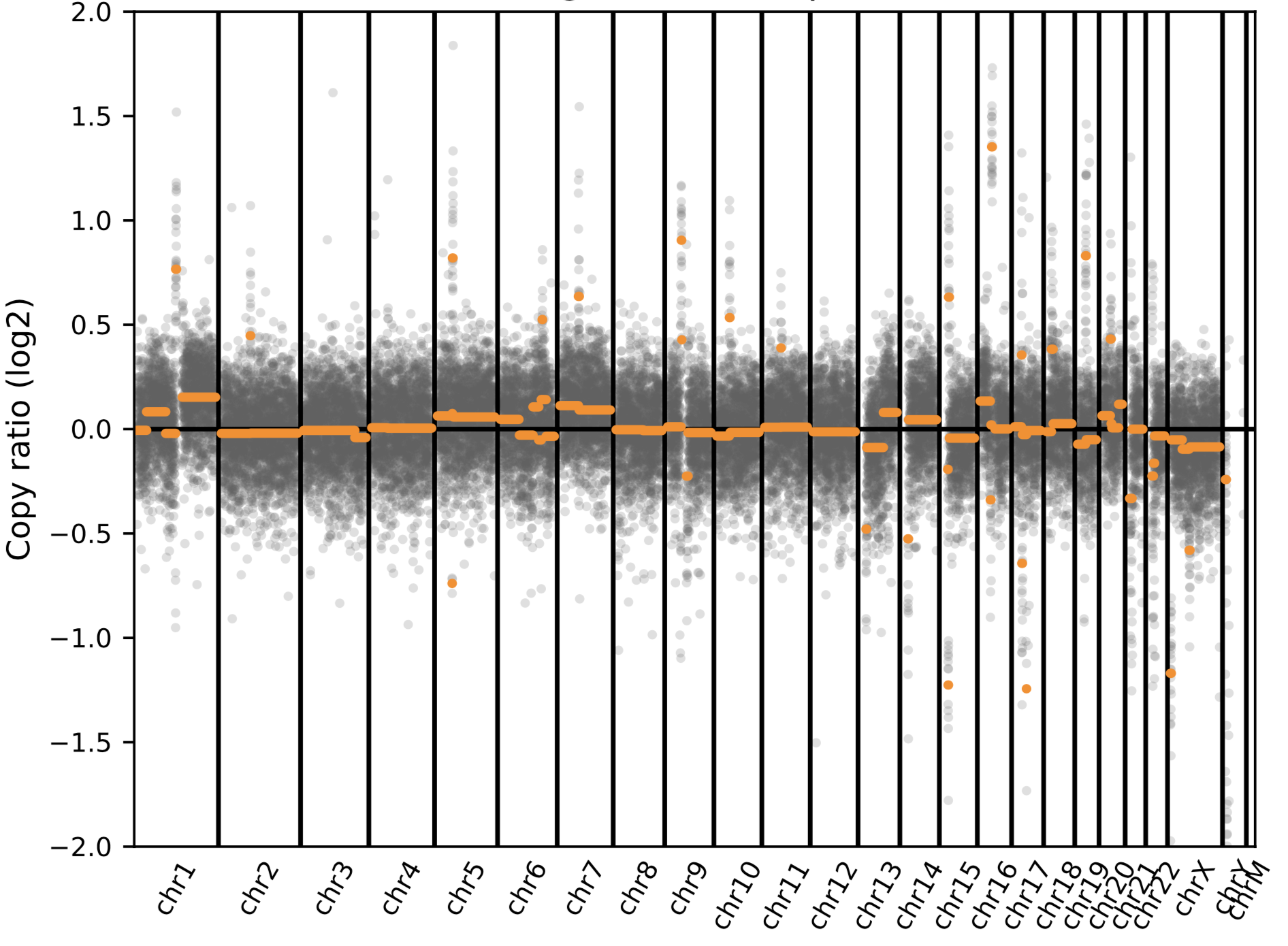 |
| --- | --- |
| Cancer Panel on Tissue (UCSF500) | 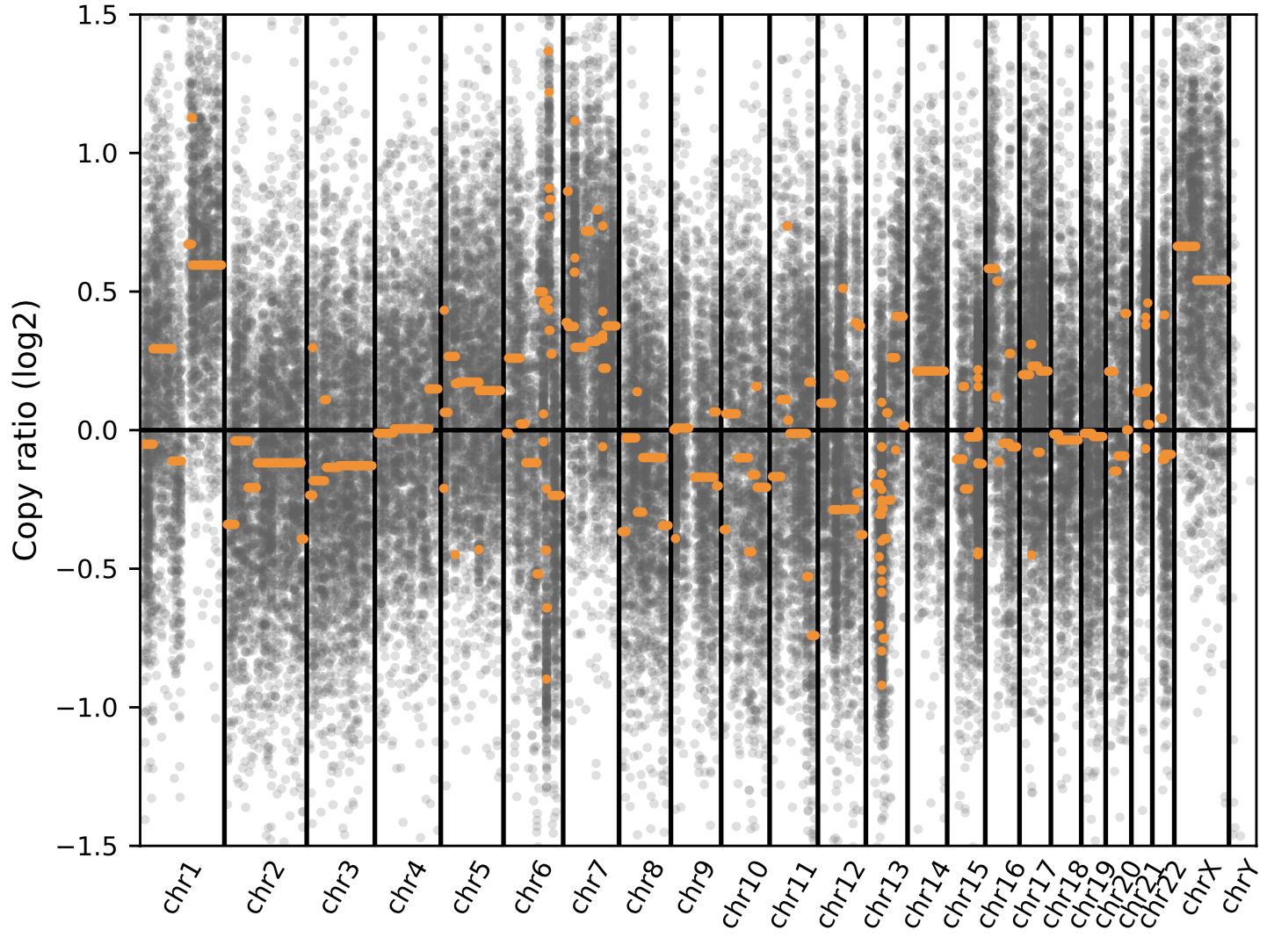 |

Sample PC23

| Peritoneal Fluid  NGS | 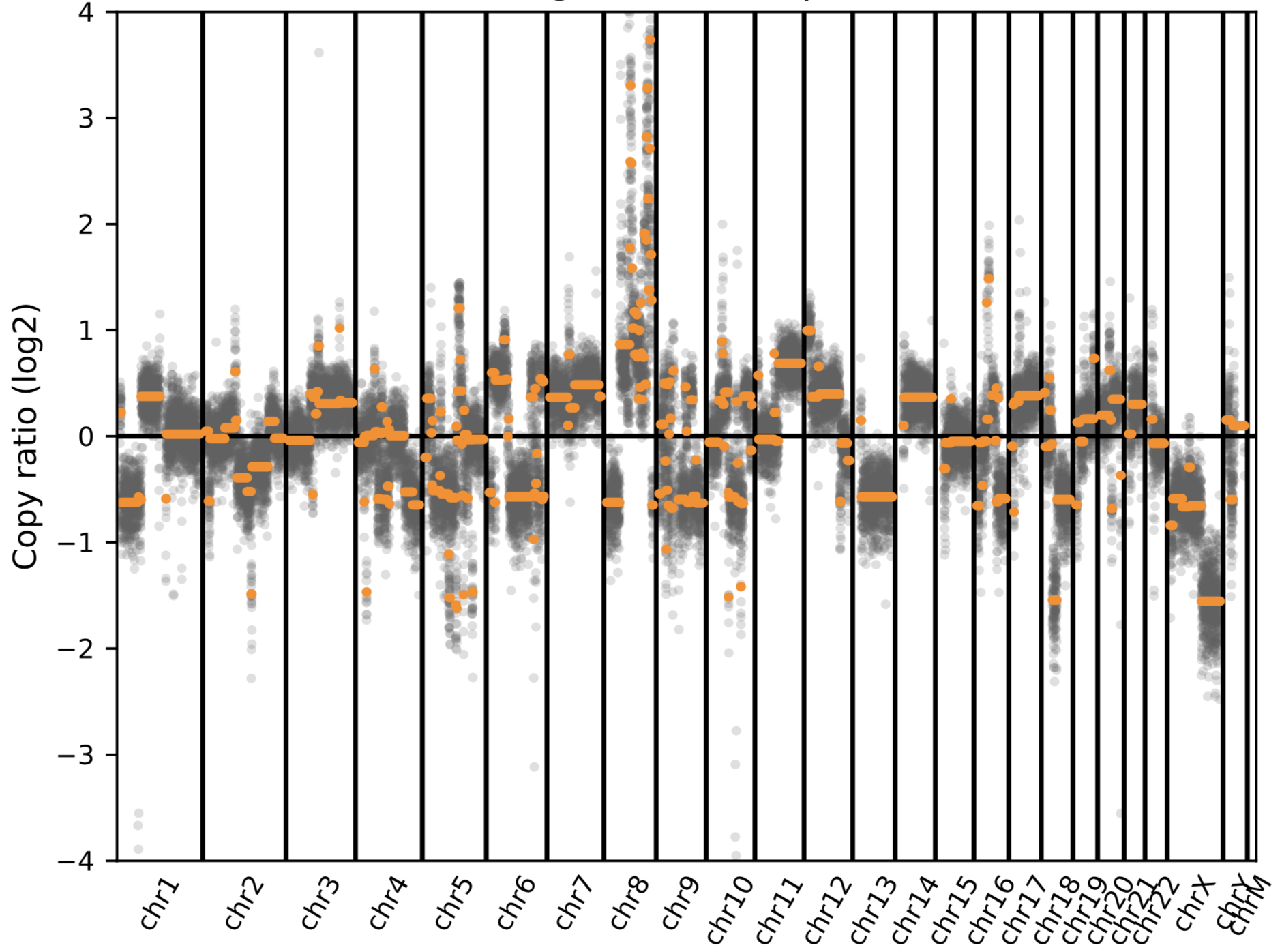 |
| --- | --- |
| Cancer Panel on Tissue (UCSF500) | 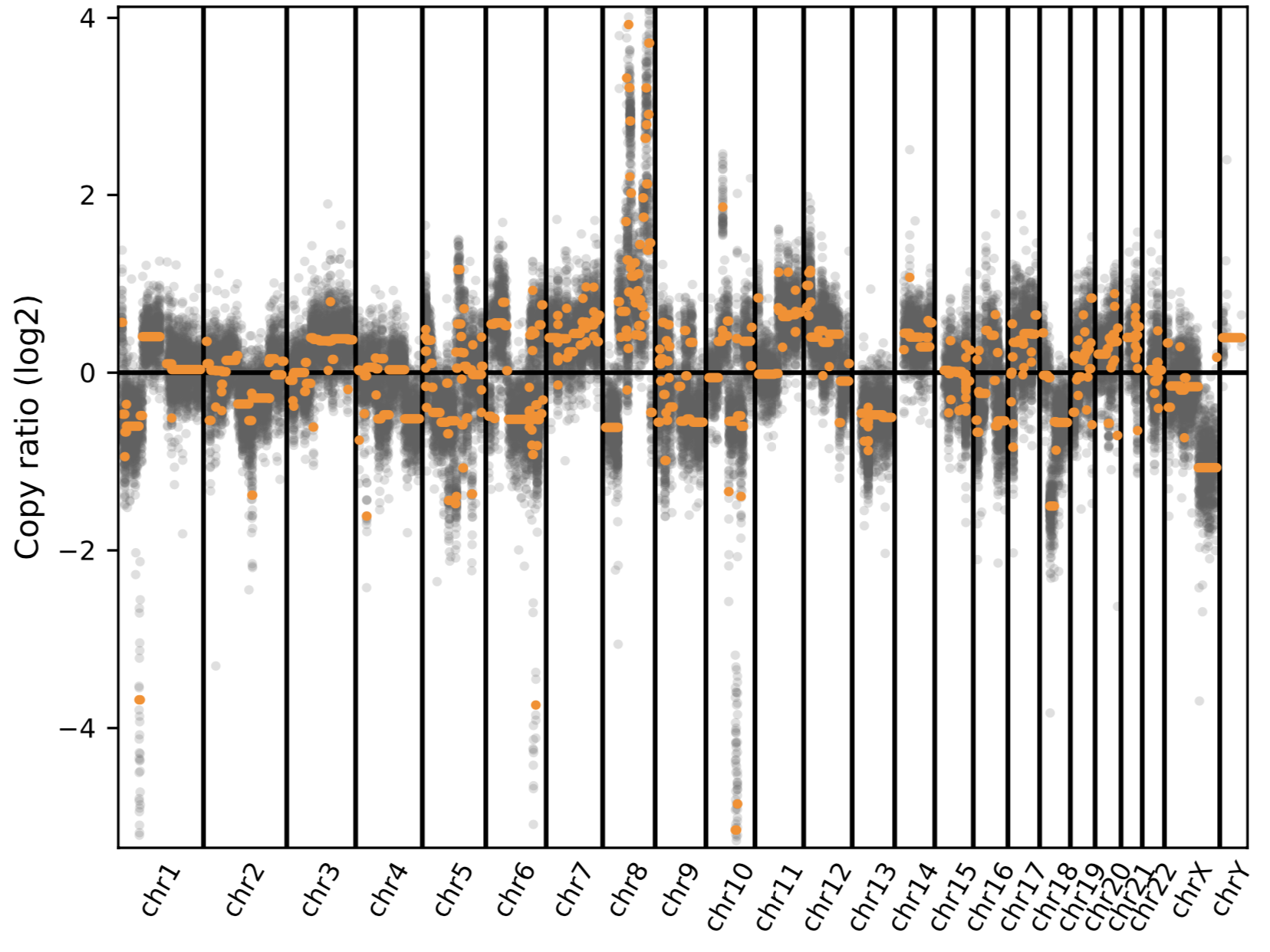 |

Sample PC38

| Pleural Fluid  NGS | 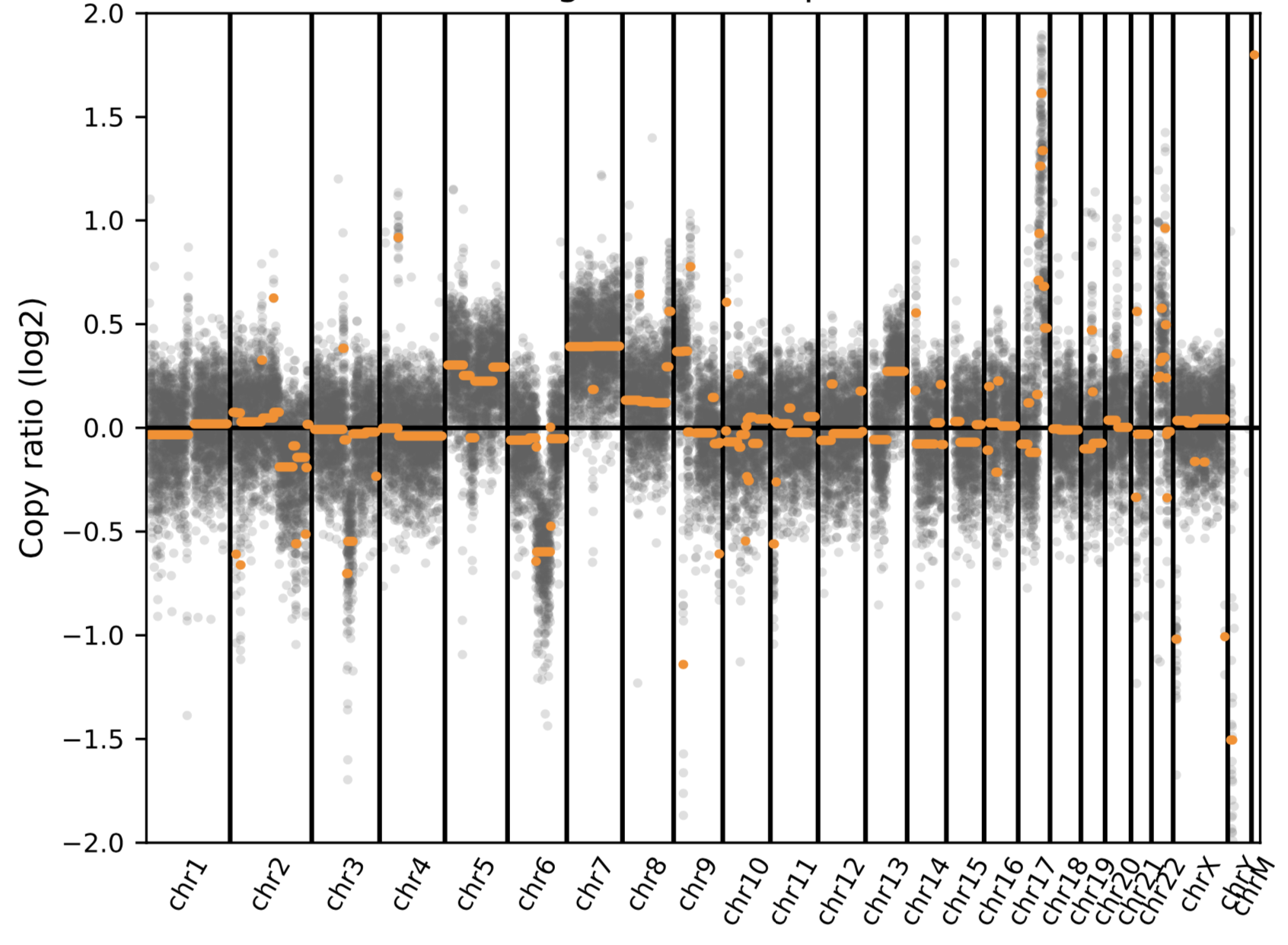 |
| --- | --- |
| Cancer Tissue | 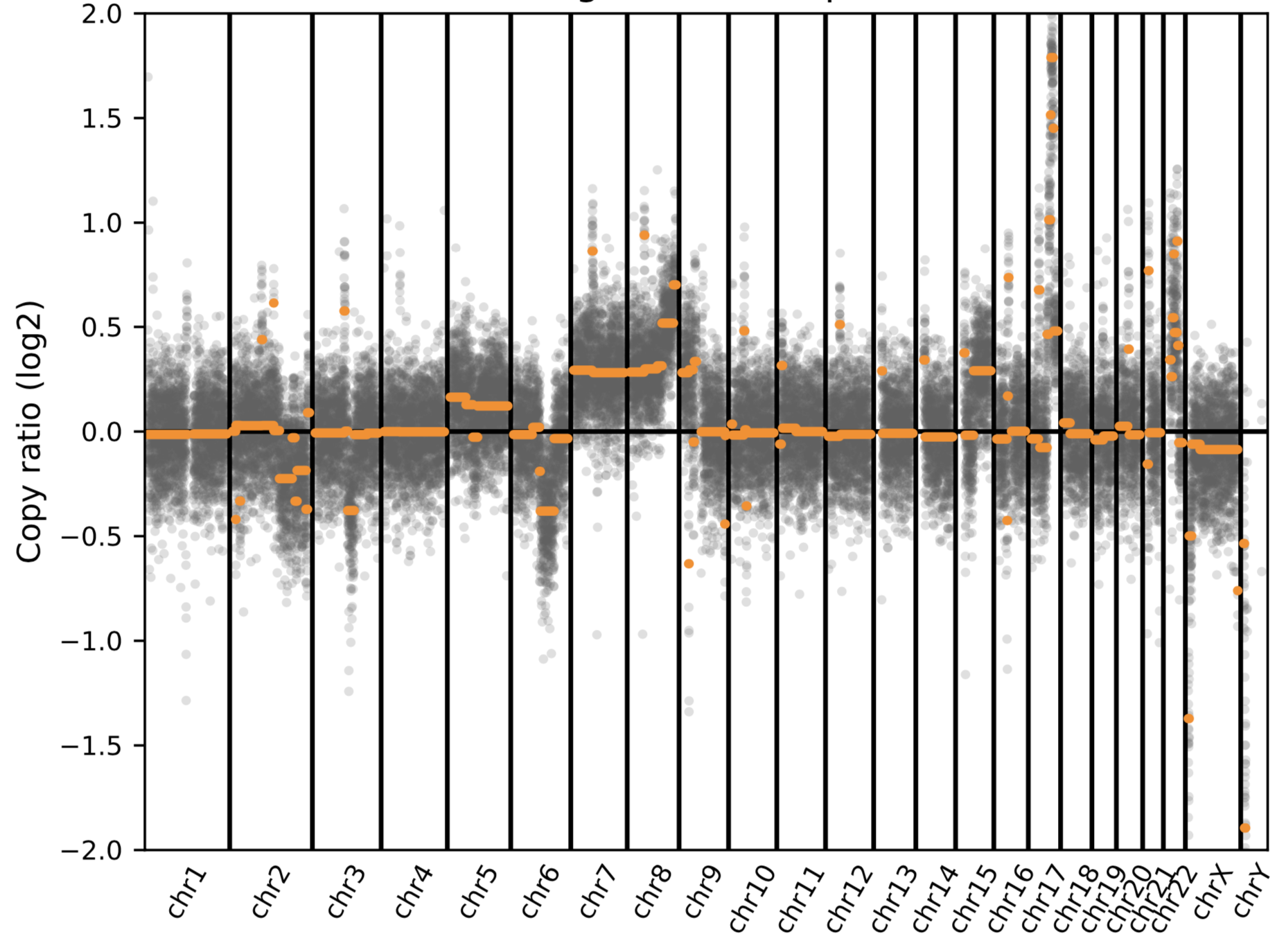 |

Sample PC39

| Pleural Fluid  NGS | 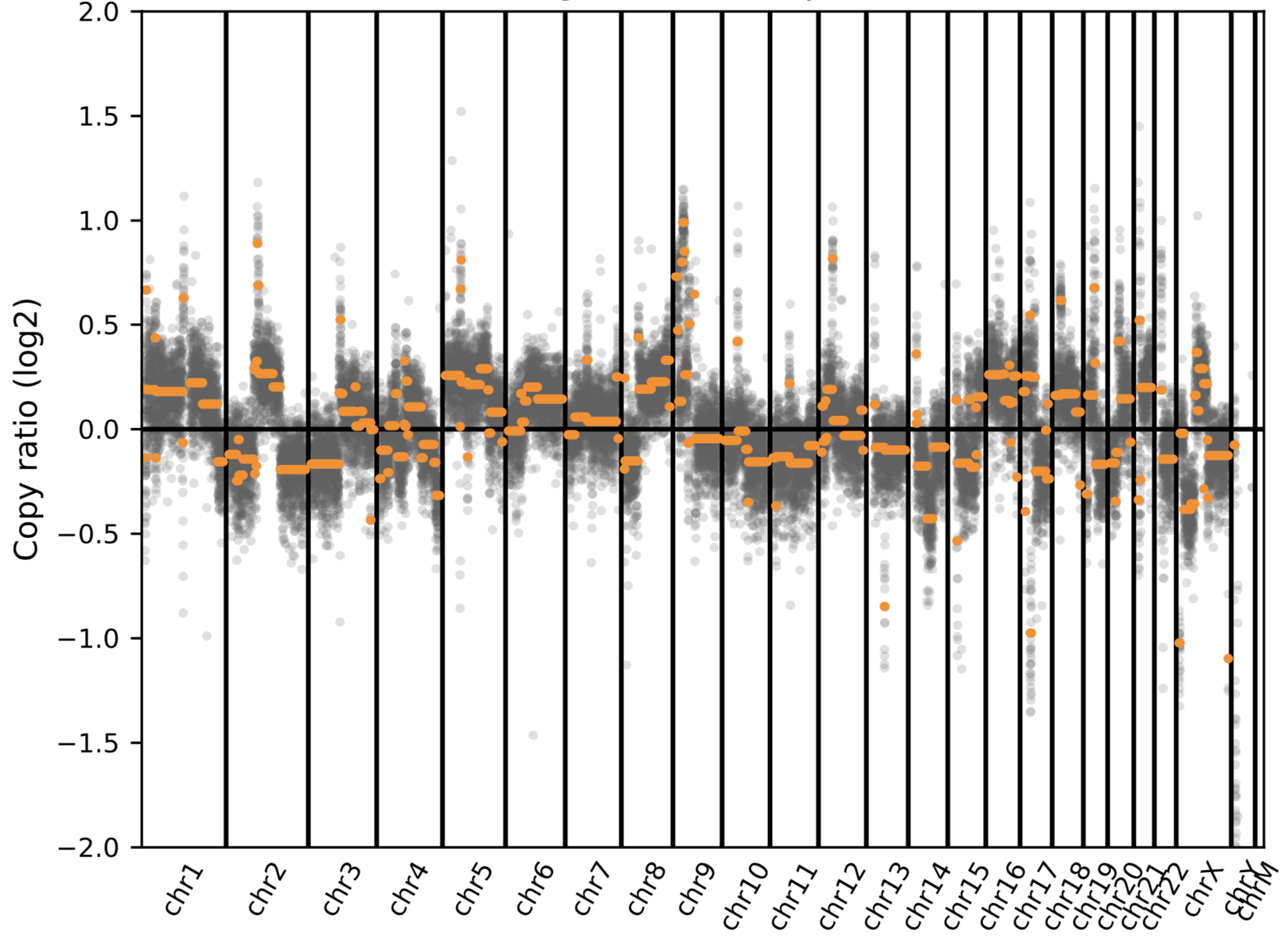 |
| --- | --- |
| Cancer Tissue | 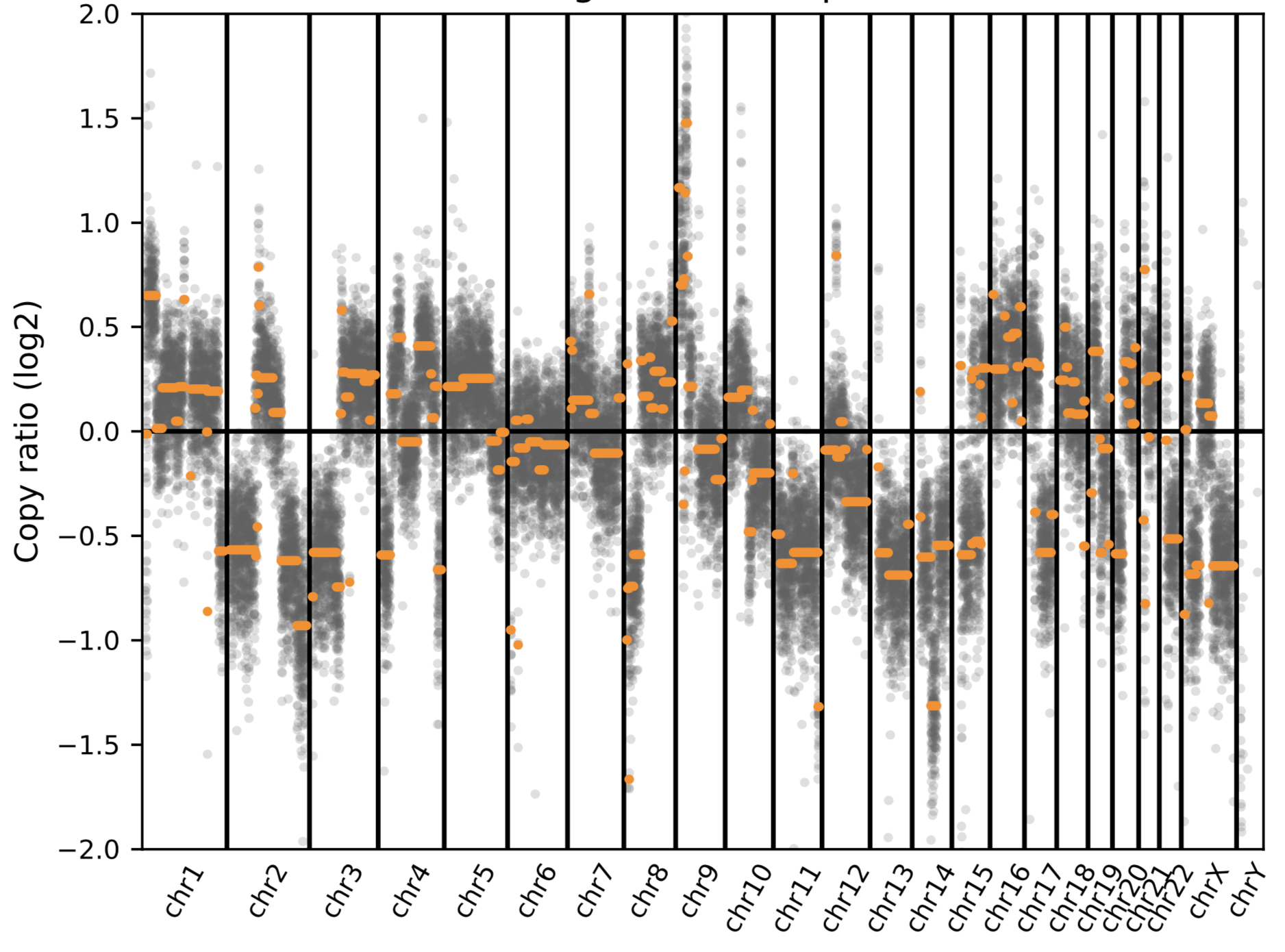 |

Sample PC41

| Pleural Fluid  NGS | 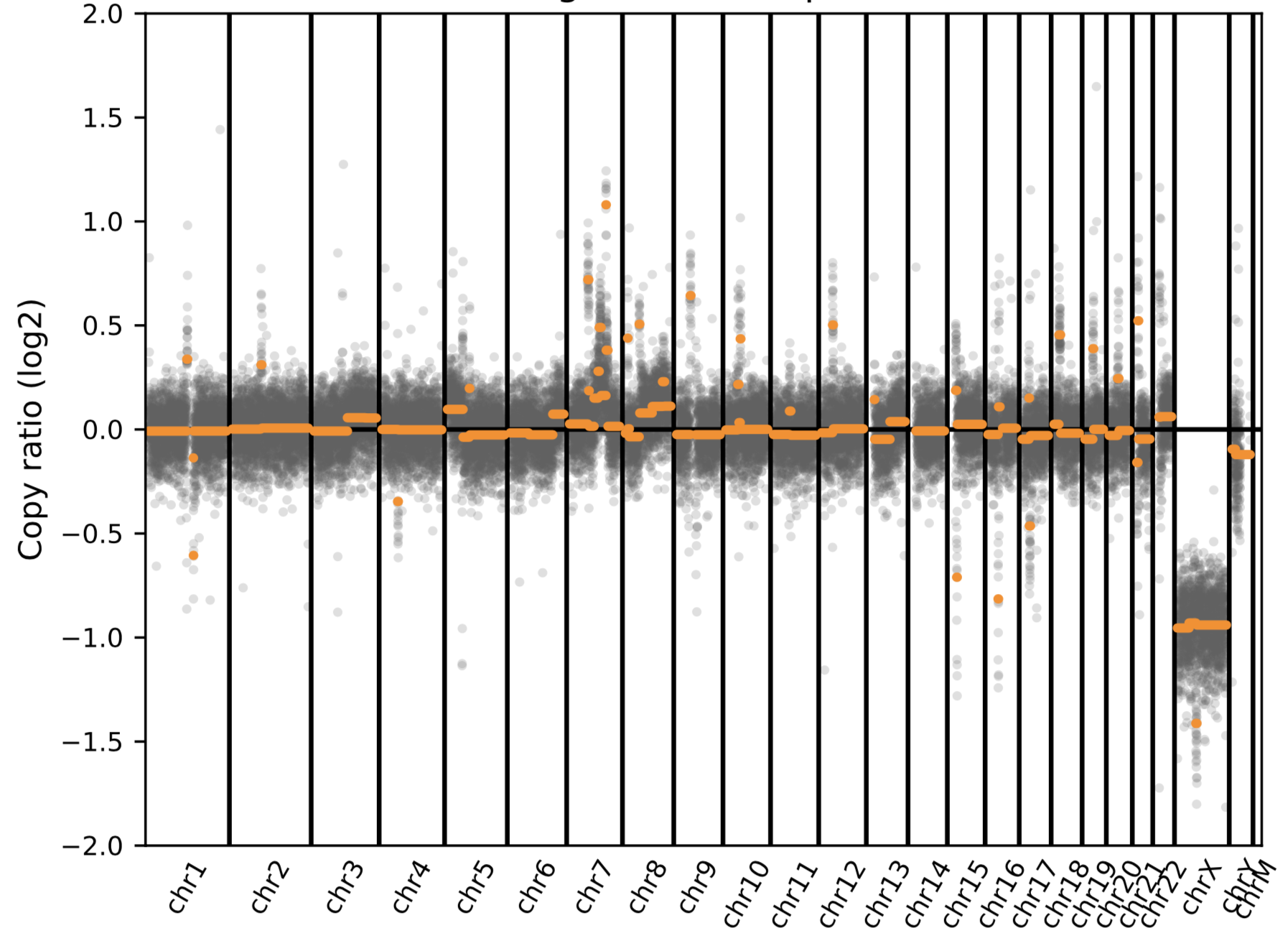 |
| --- | --- |
| Cancer Tissue | 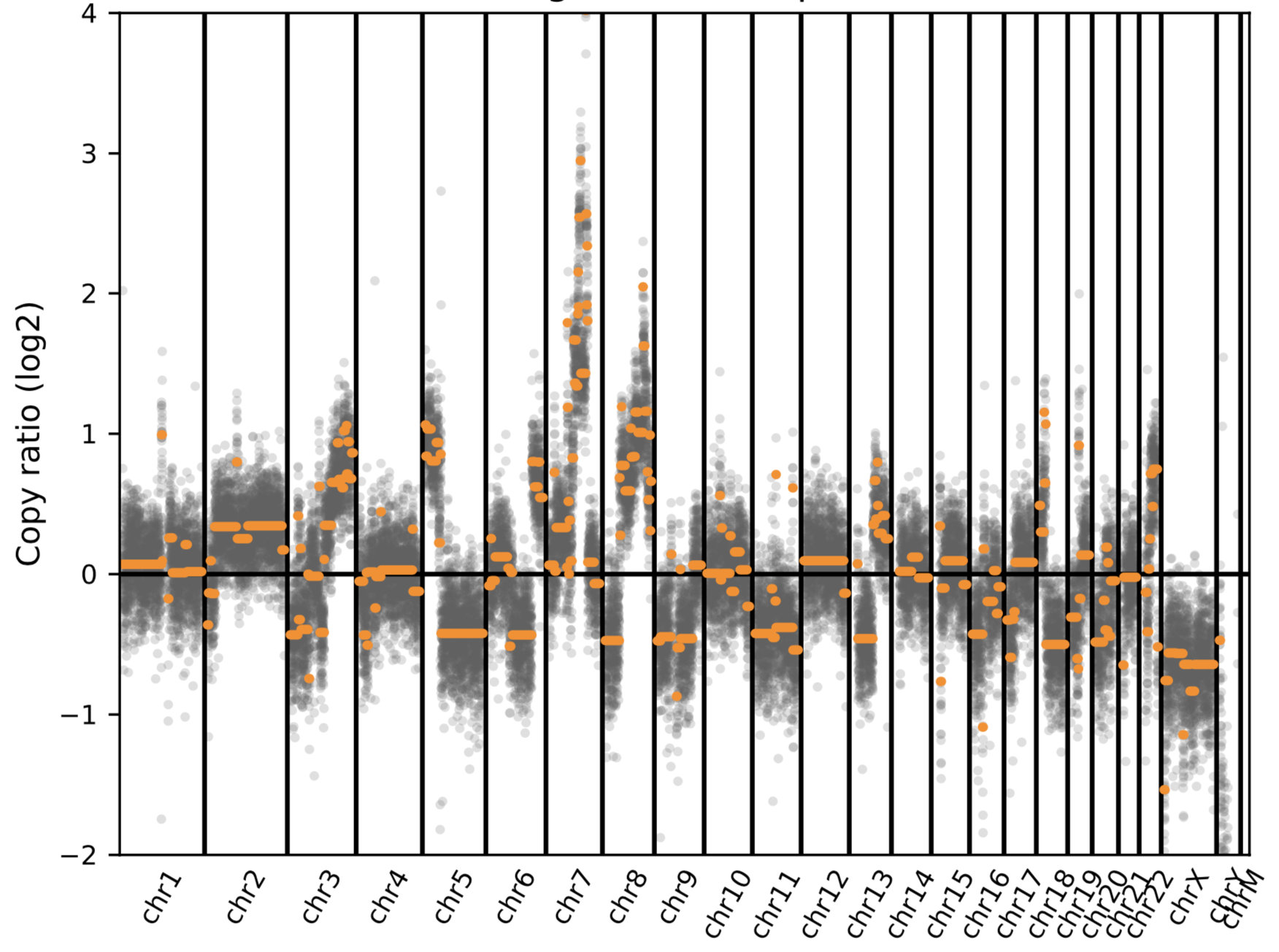 |

Sample PC44

| Pleural Fluid  NGS | 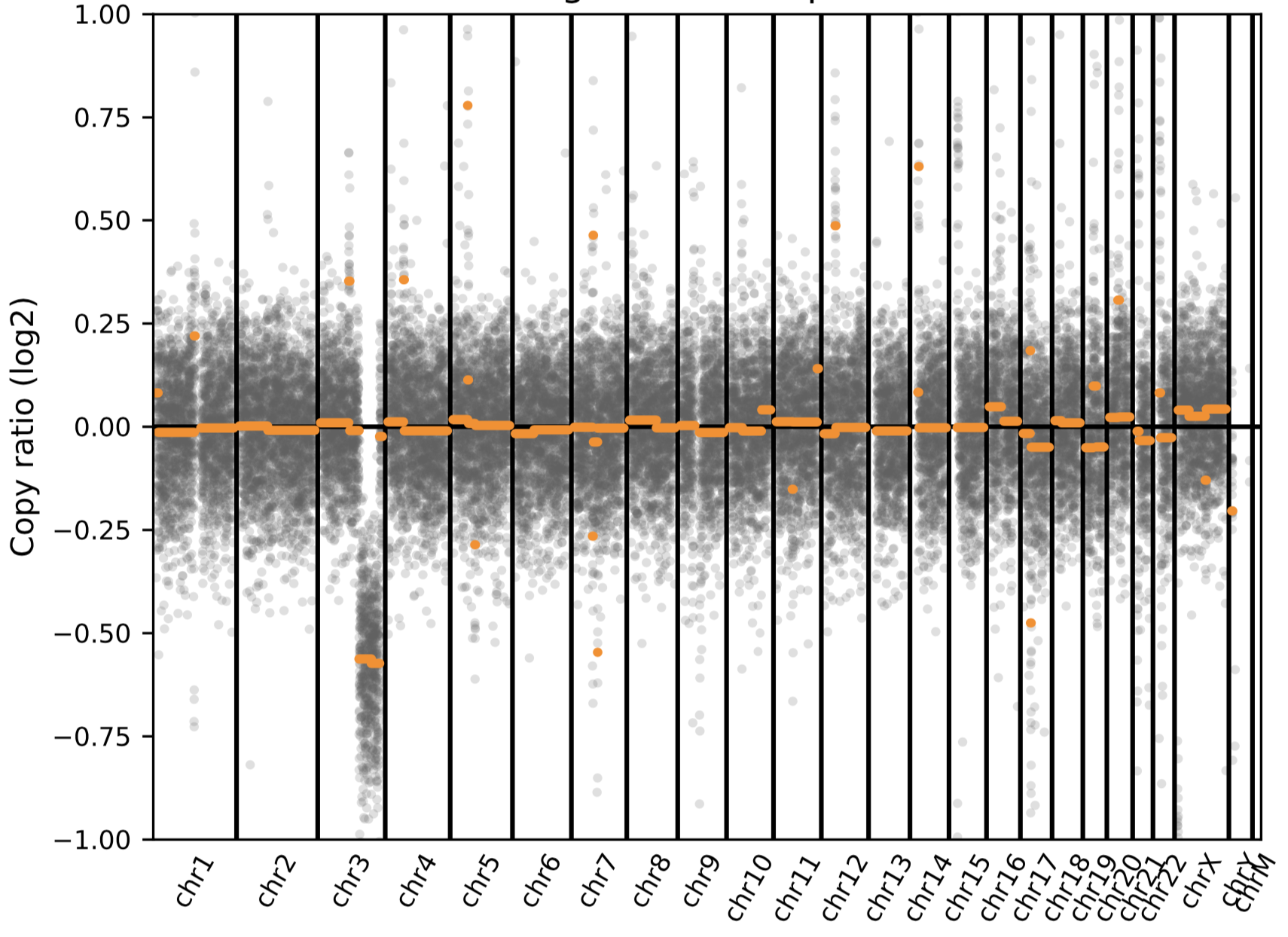 |
| --- | --- |
| Cancer Panel on Tissue (UCSF500) | 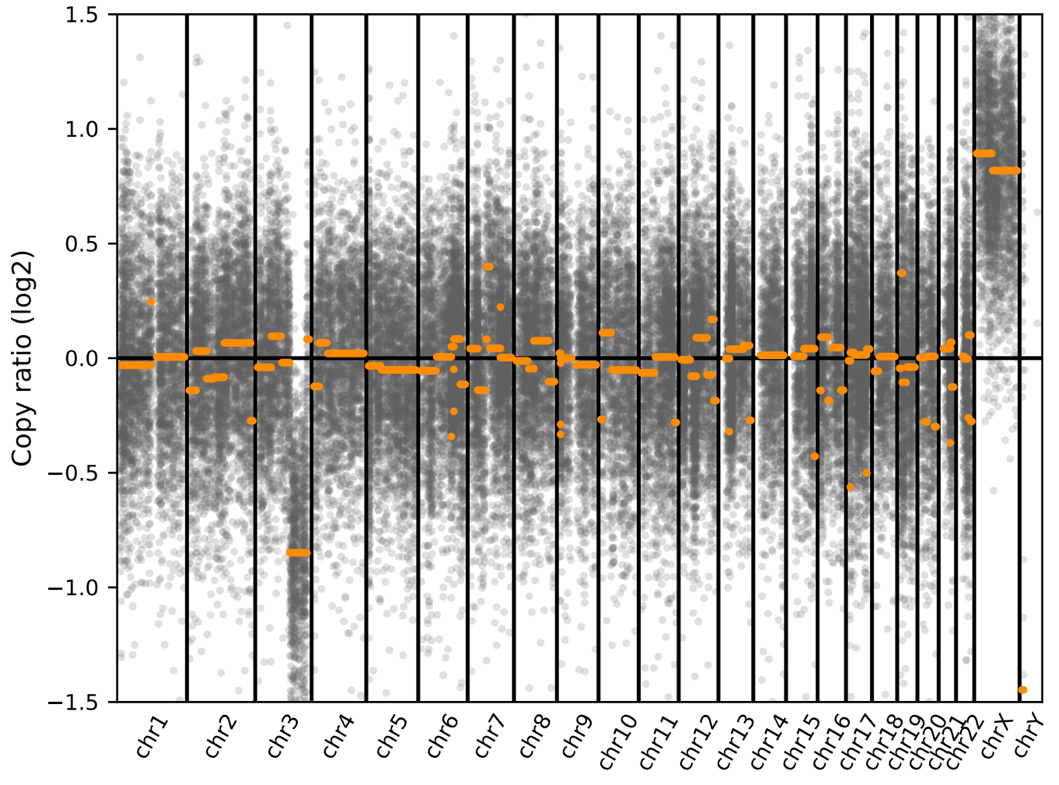 |

Sample PC46: This Hodgkin lymphoma was positive by mNGS, but negative by tissue NGS (the highest tumor percentage site was selected). The Reed-Sternberg cell, the cancer cell in Hodgkin lymphoma, is typically surrounded by many more non-cancerous cells in tissue. A hematopathologist (LW) determined that the tumor percentage in this case was less than 20% by histology.

| Pleural Fluid  NGS | 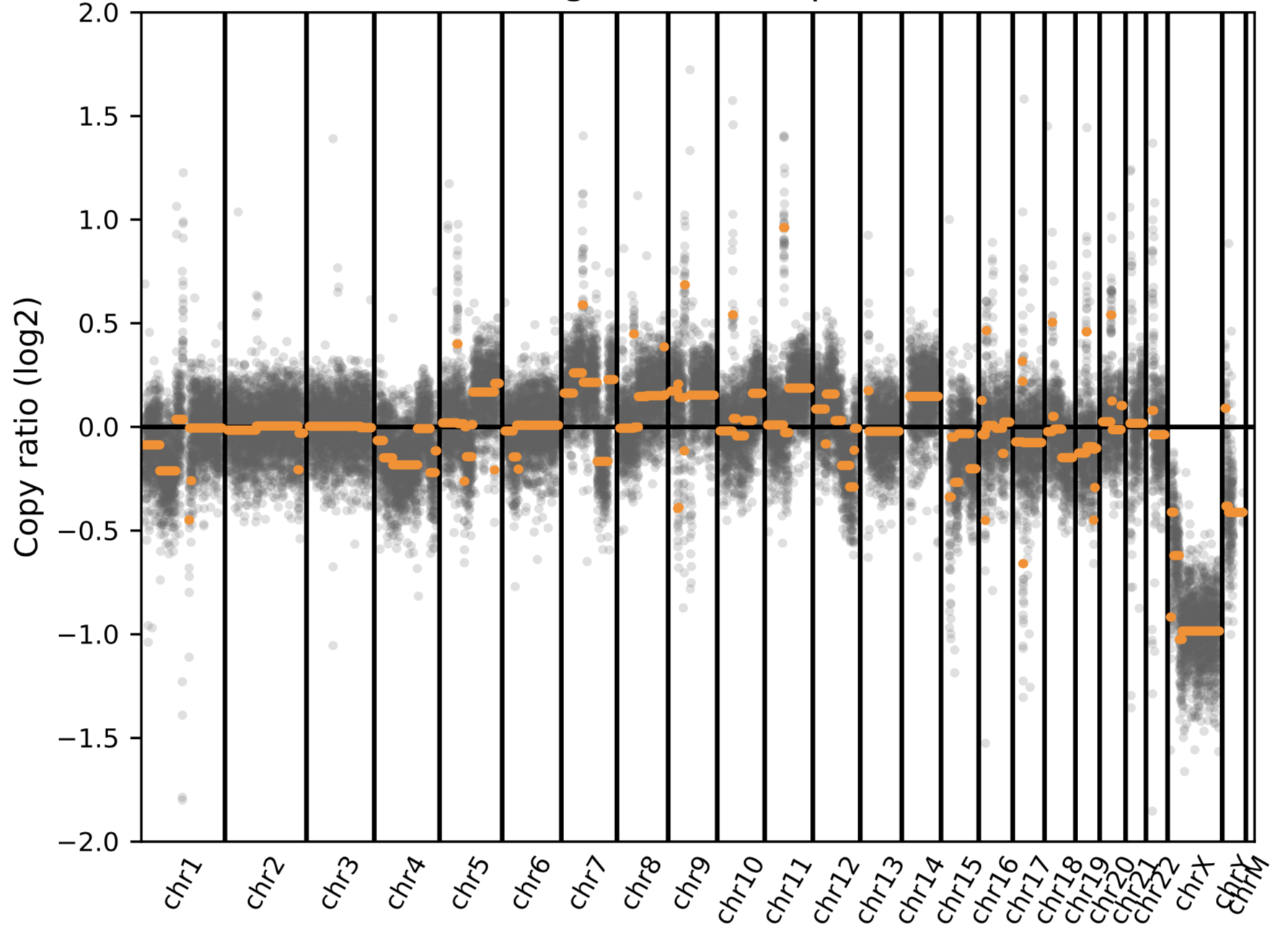 |
| --- | --- |
| Cancer Tissue | 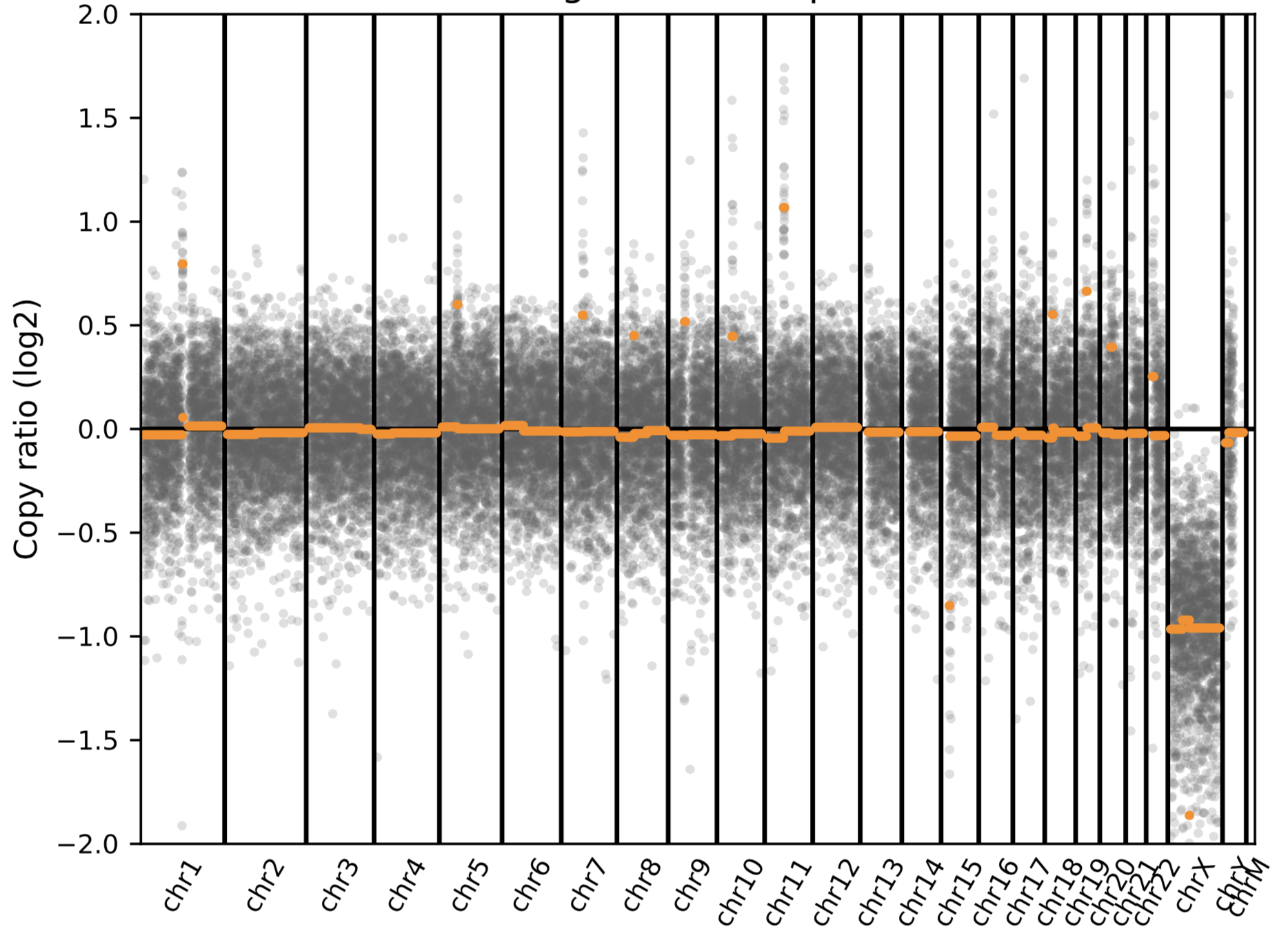 |

**Example of Negative Result**

Case NC108:


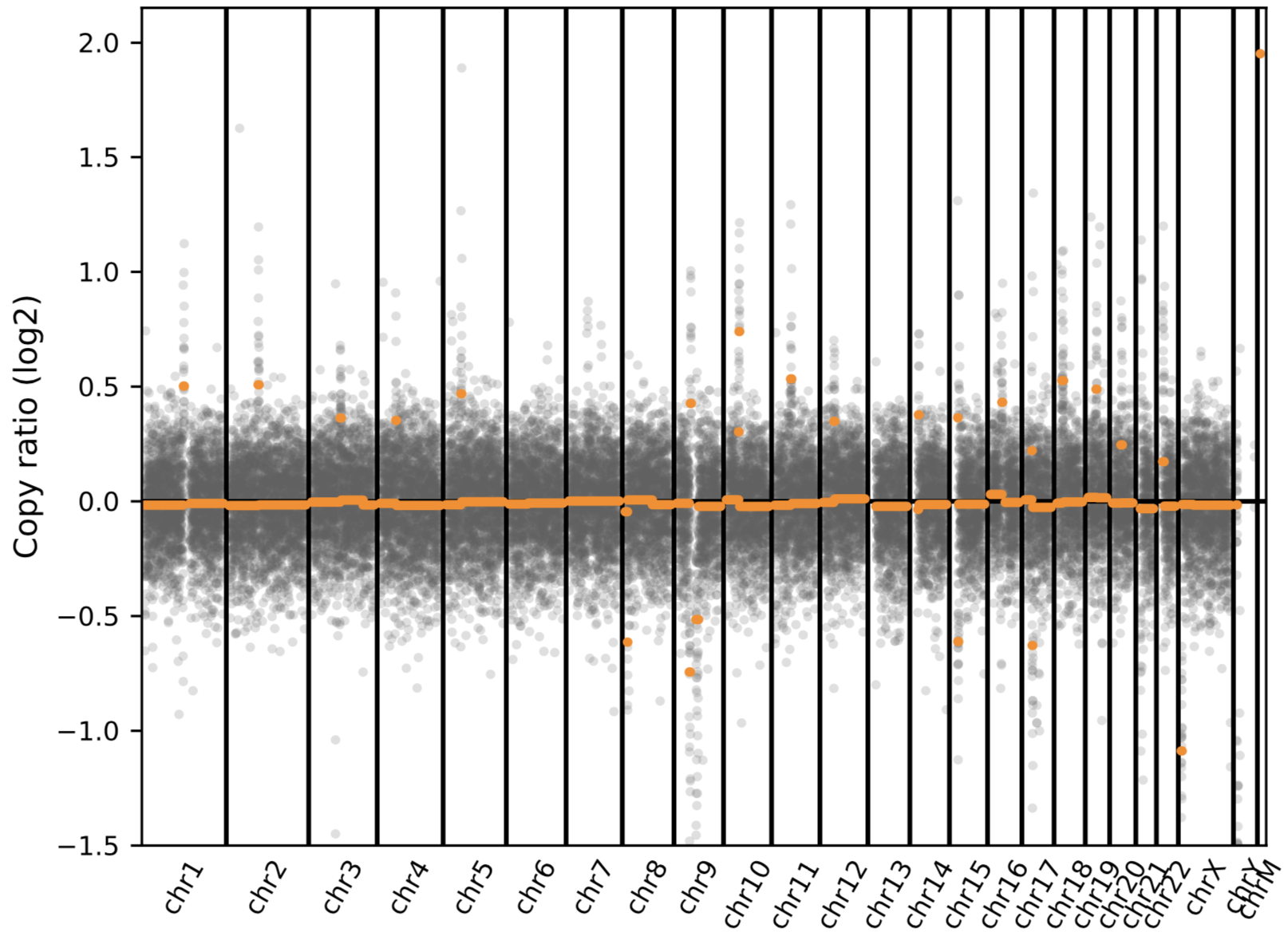


**Read Depth Analysis**

Physical dilution of a plasma control:

| 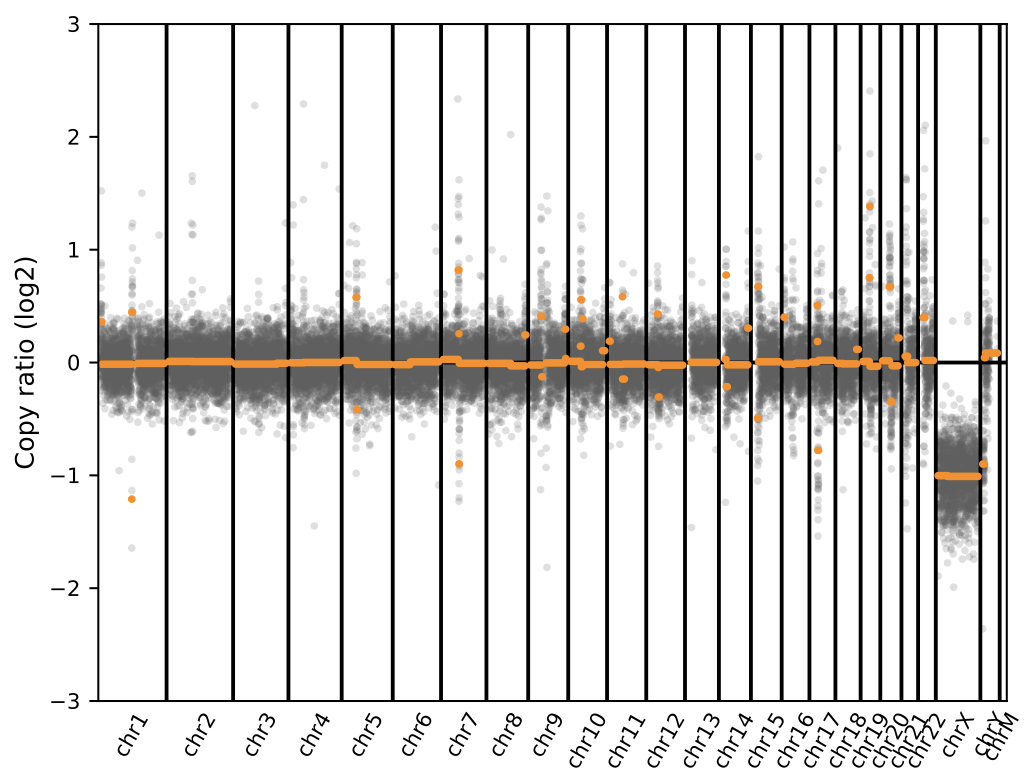 | 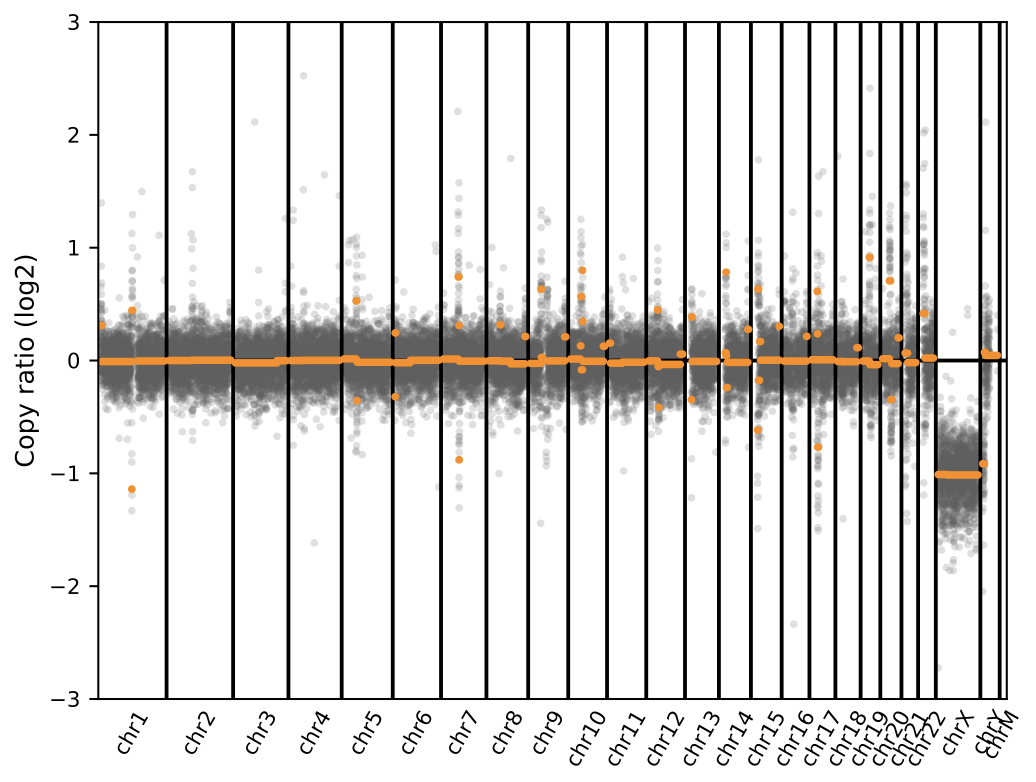 | 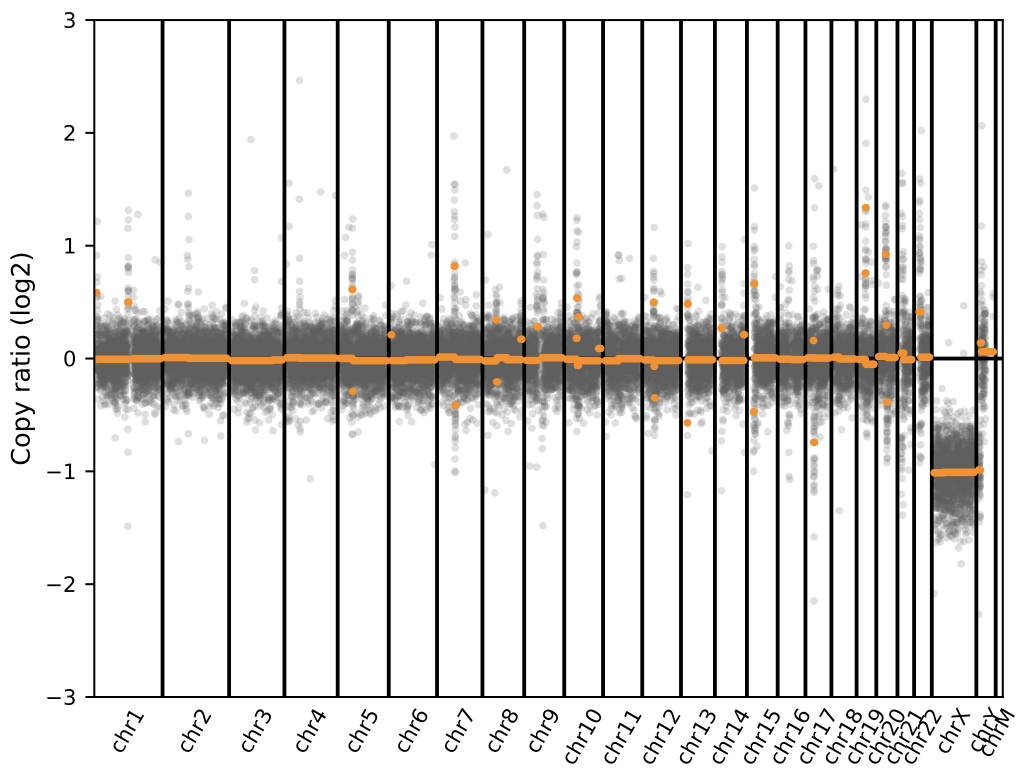 | 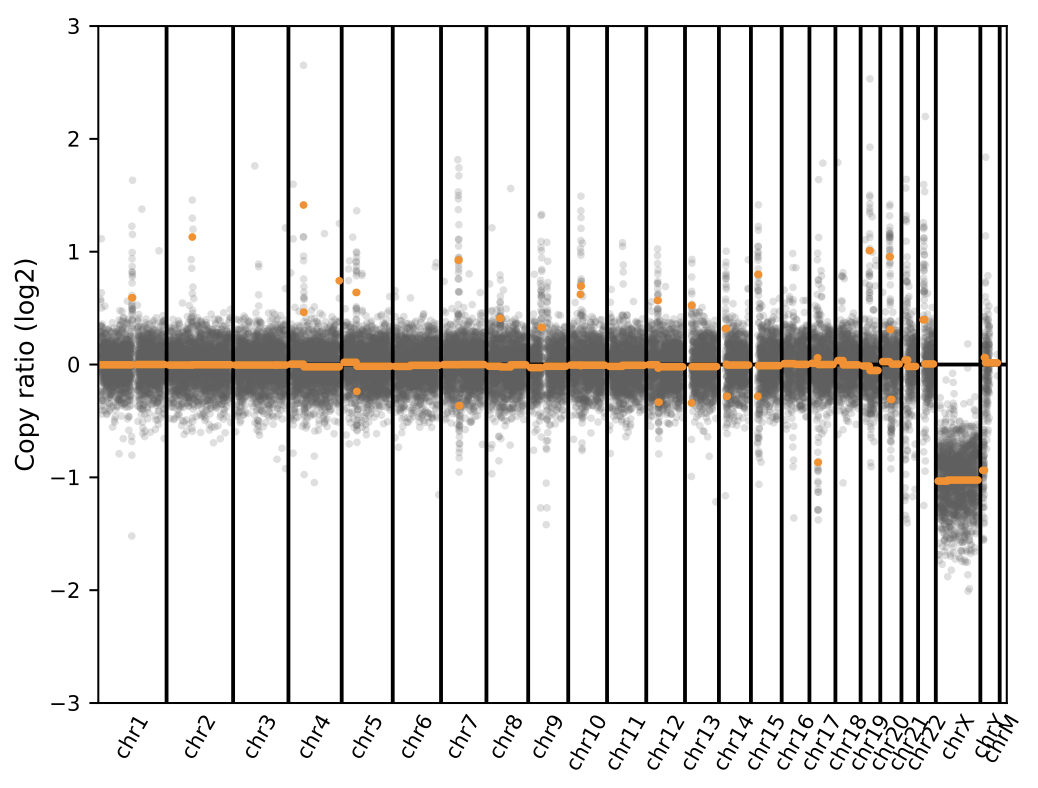 |
| --- | --- | --- | --- |
| 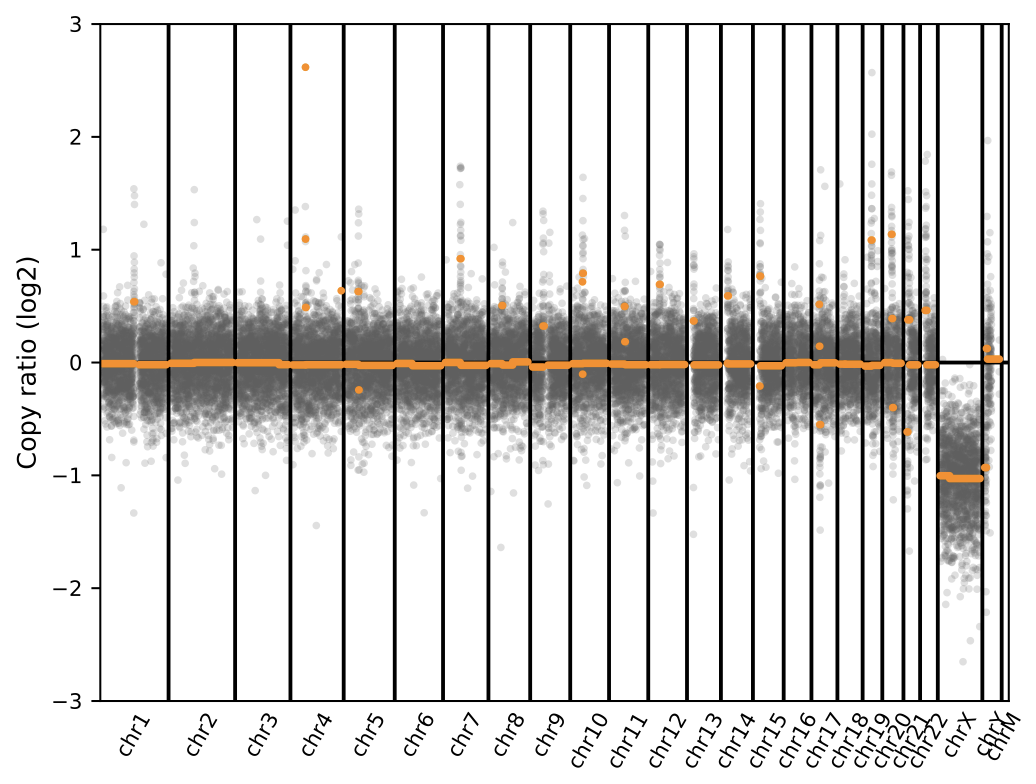 | 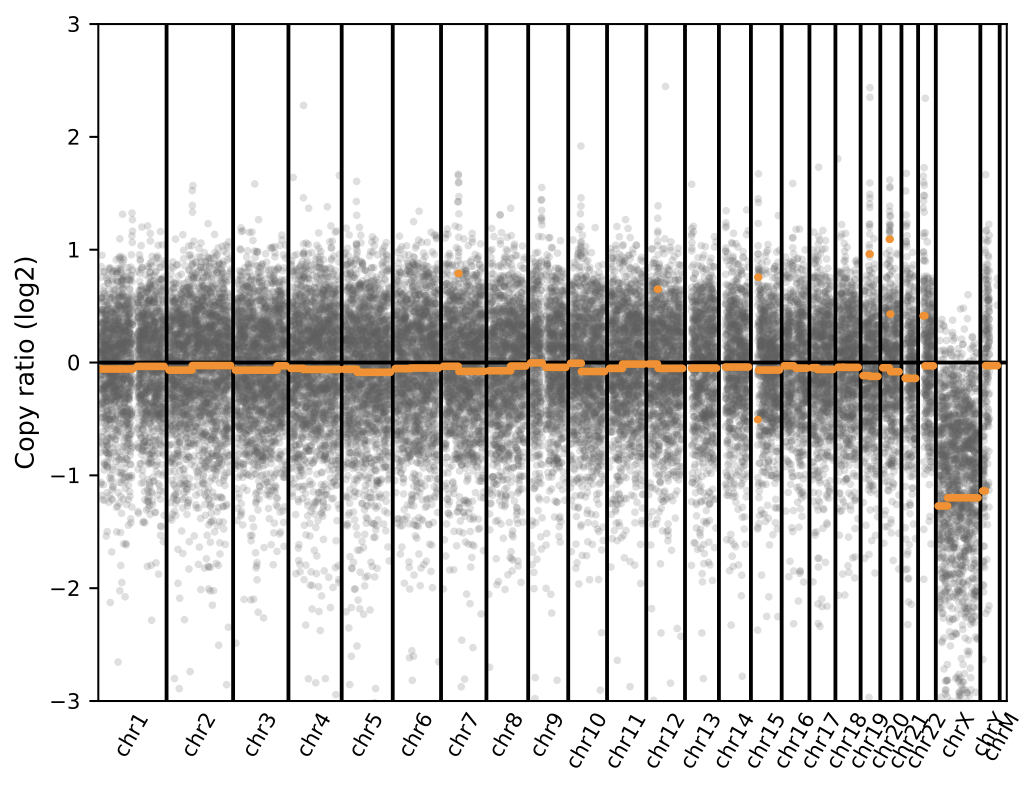 | 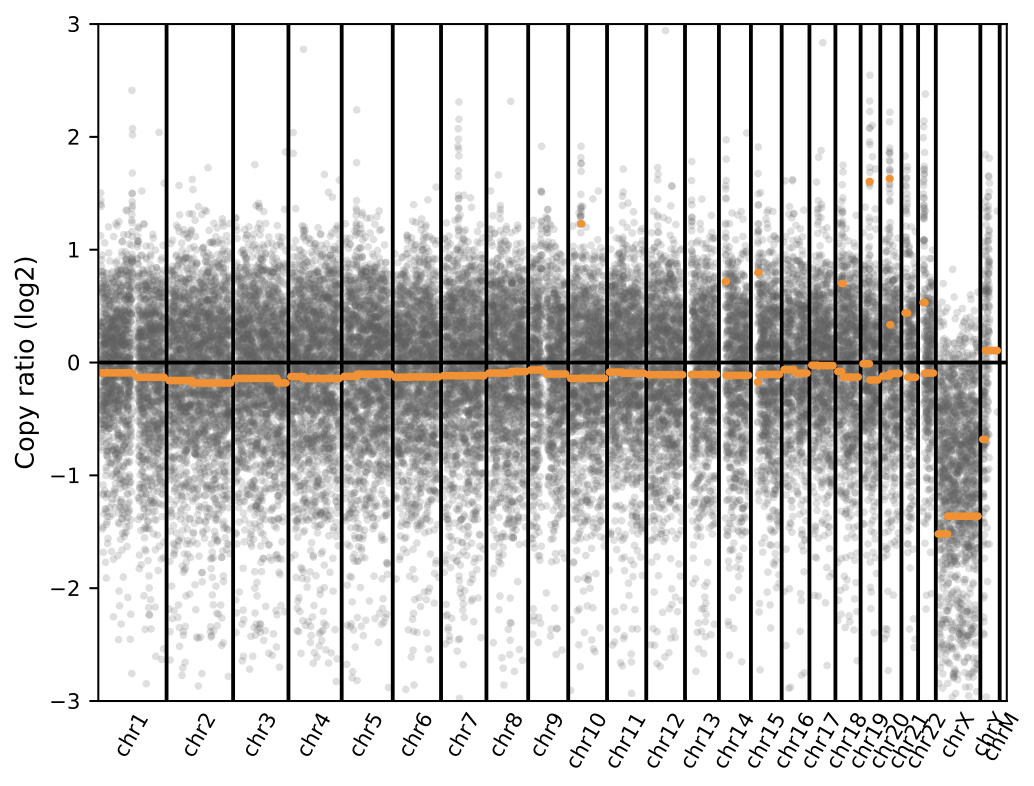 | 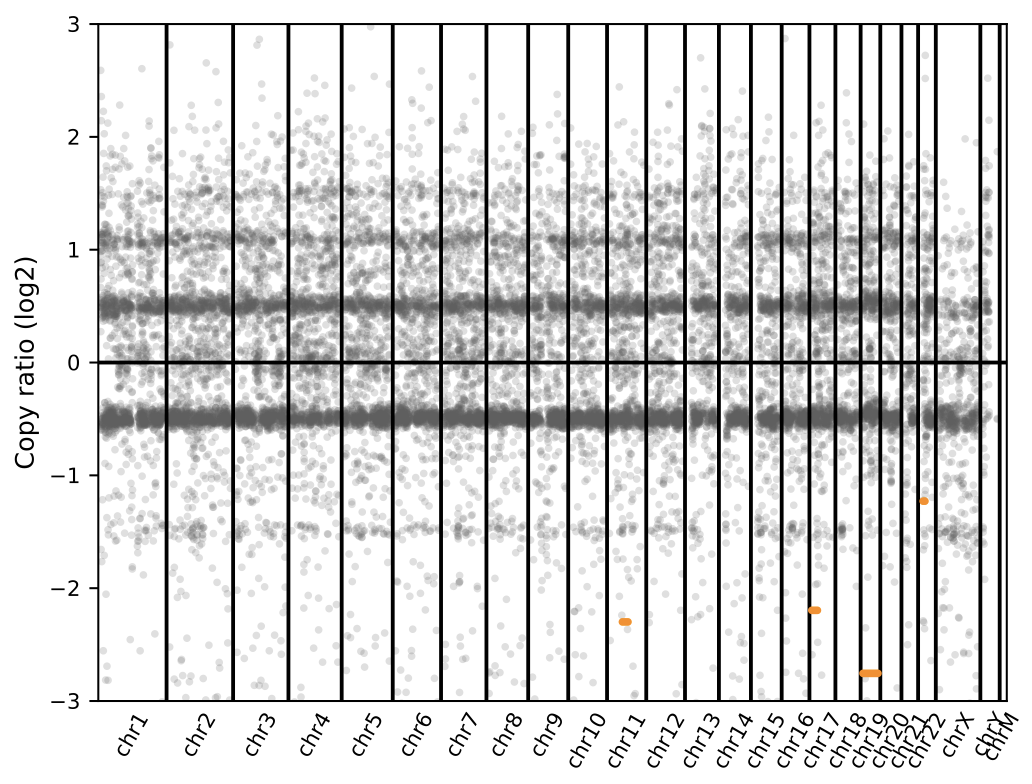 |

From left to right, top down: 1, 4, 16, 64, 256, 1024, 4096, 16384-fold dilutions of the original sample at 6.6 ng per 1 mL of plasma. The results are interpretable down to 1.6 pg input.

Read downsampling of a plasma control:

| 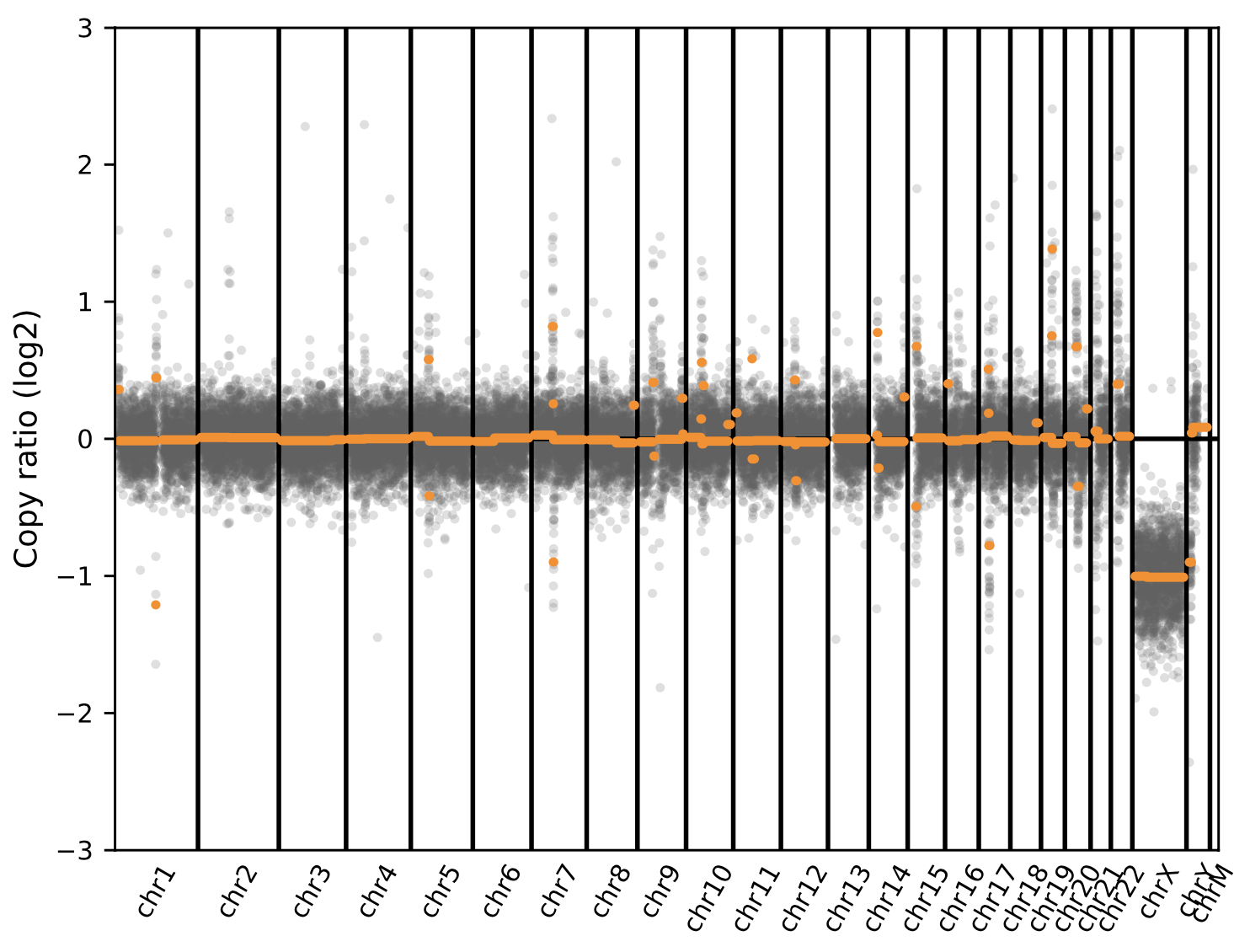 | 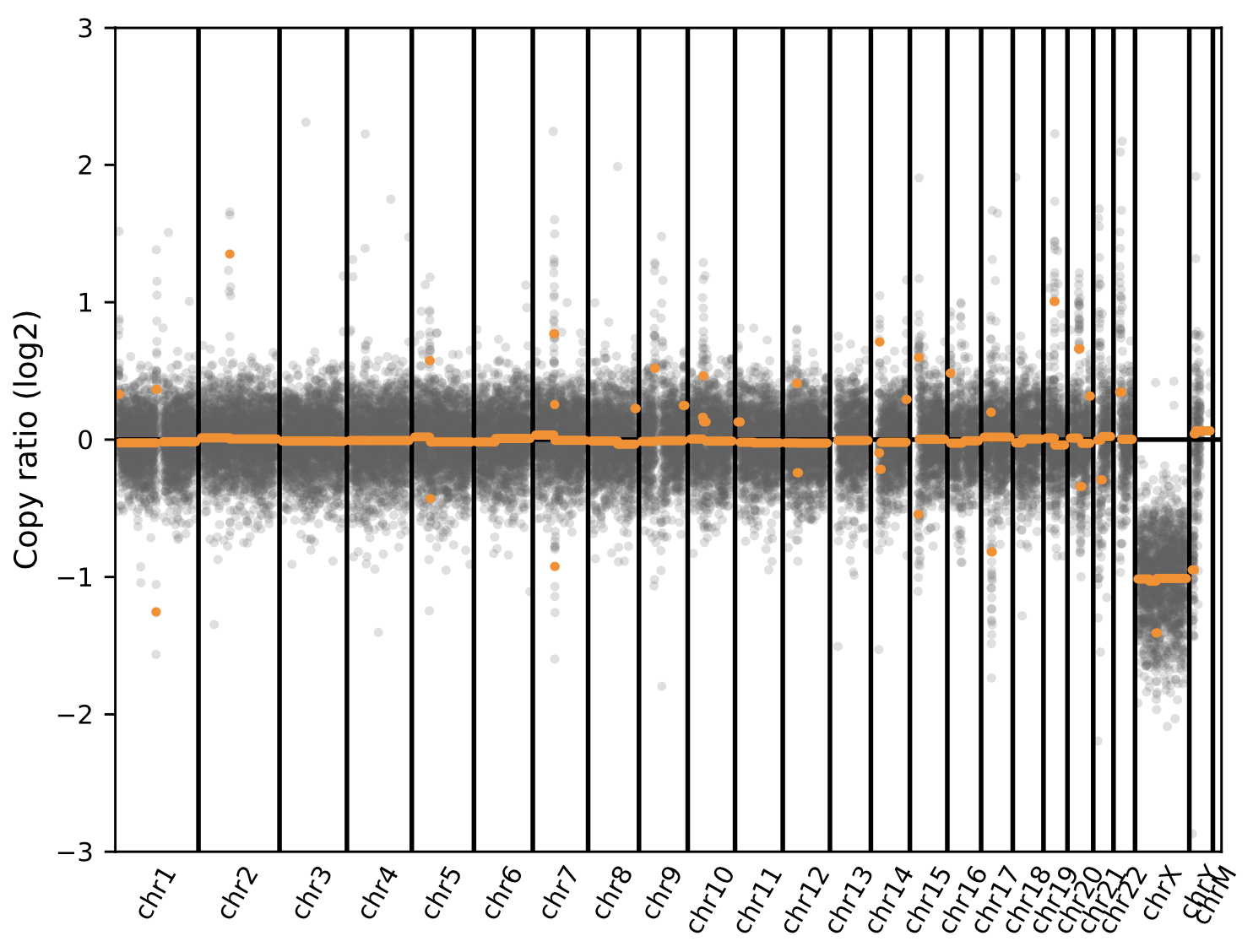 | 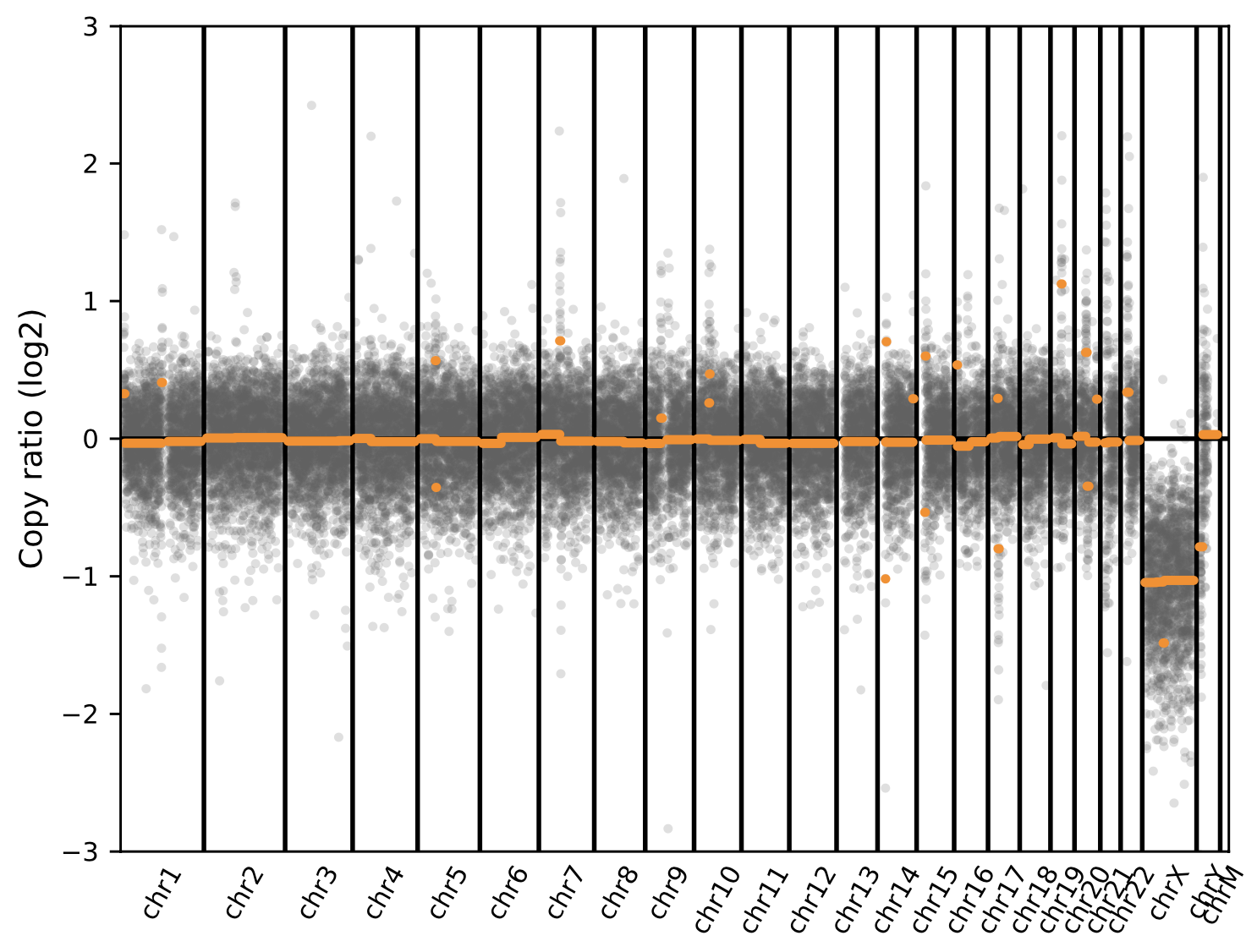 | 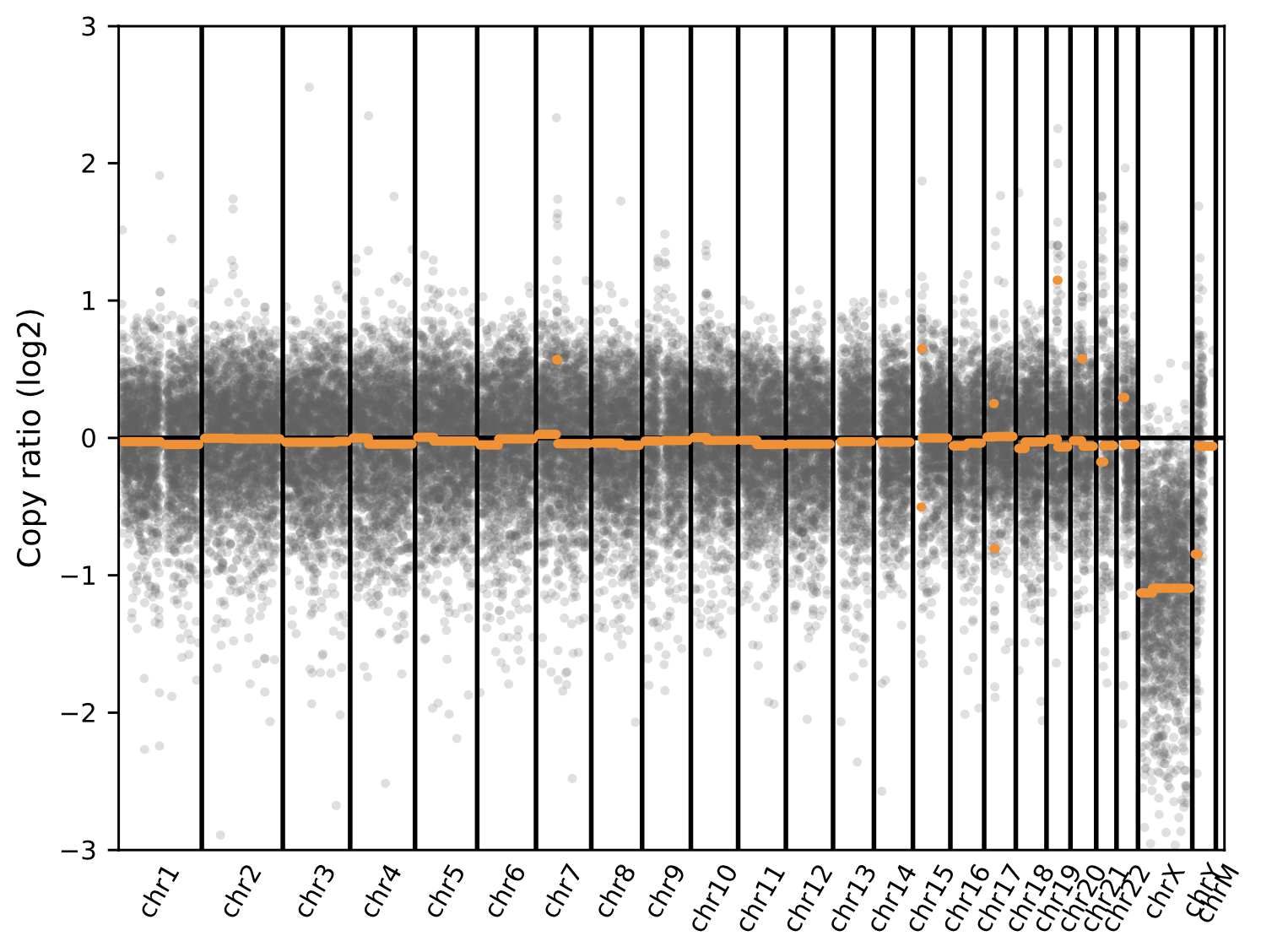 |
| --- | --- | --- | --- |
| 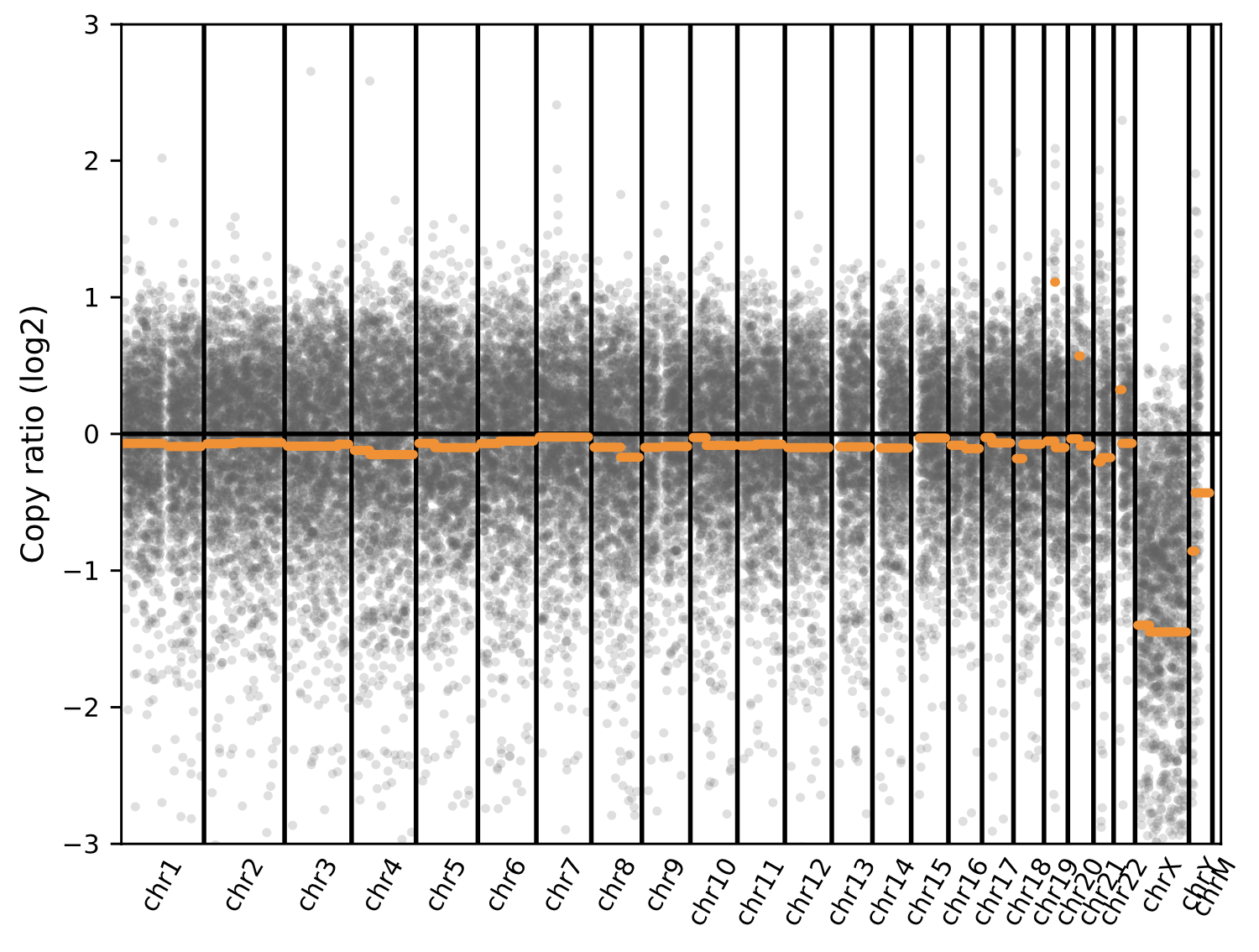 | 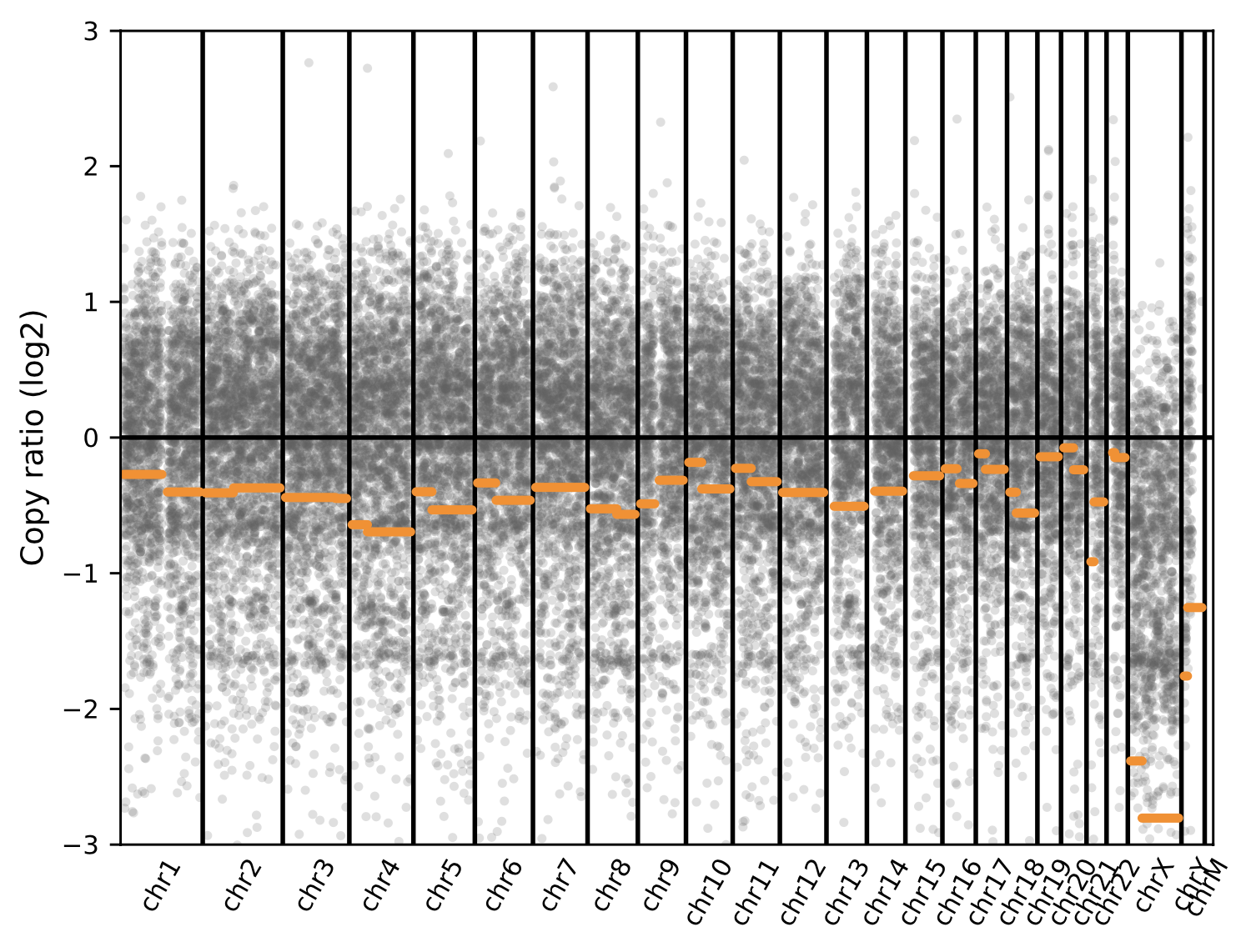 | 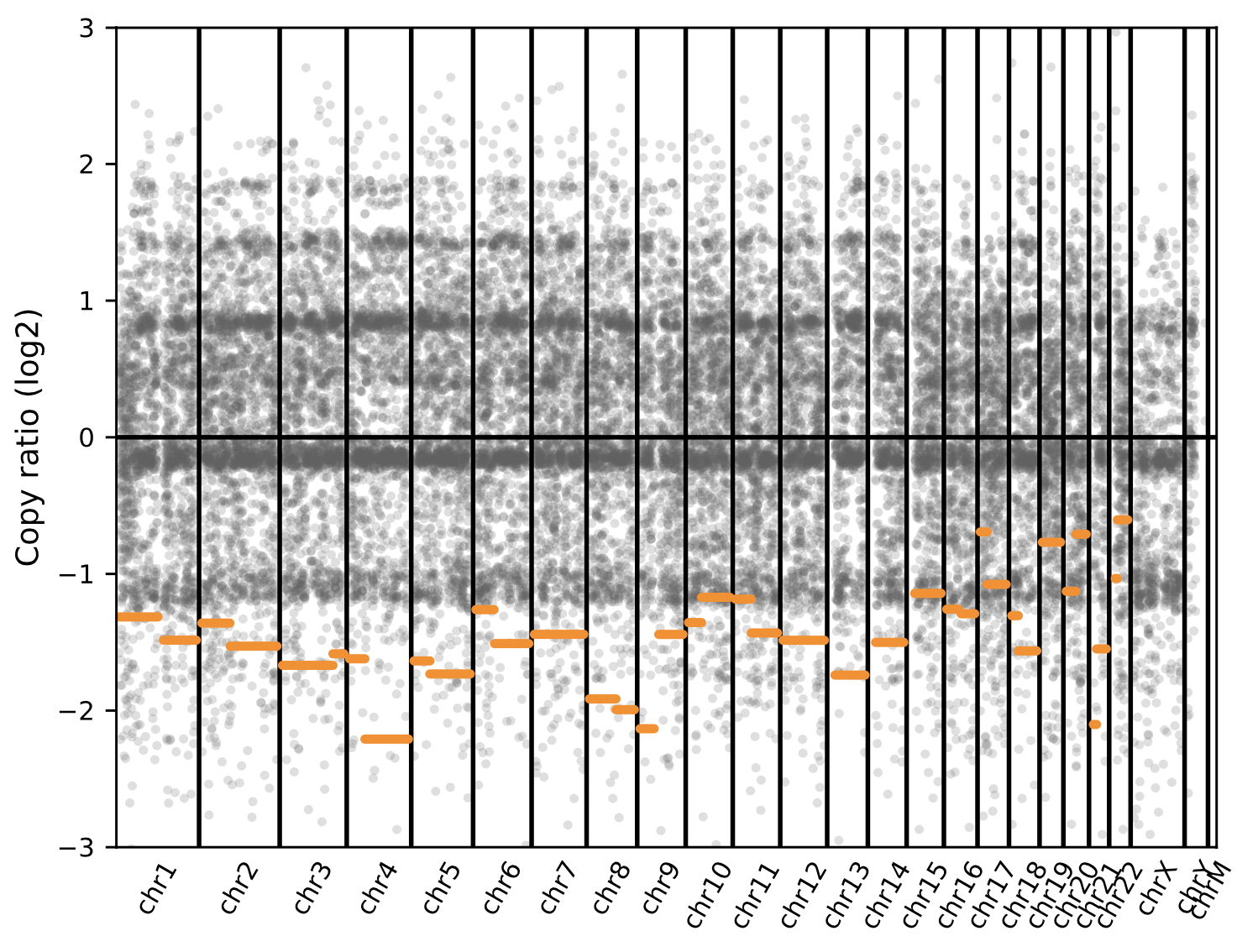 |  |

From left to right, top down: 1, 2, 4, 8, 16, 32, 64-fold computational downsampling of the original sample reads at 4.4 M paired end reads. The results are interpretable down to 276 thousand paired reads.

Read downsampling of PC35

| 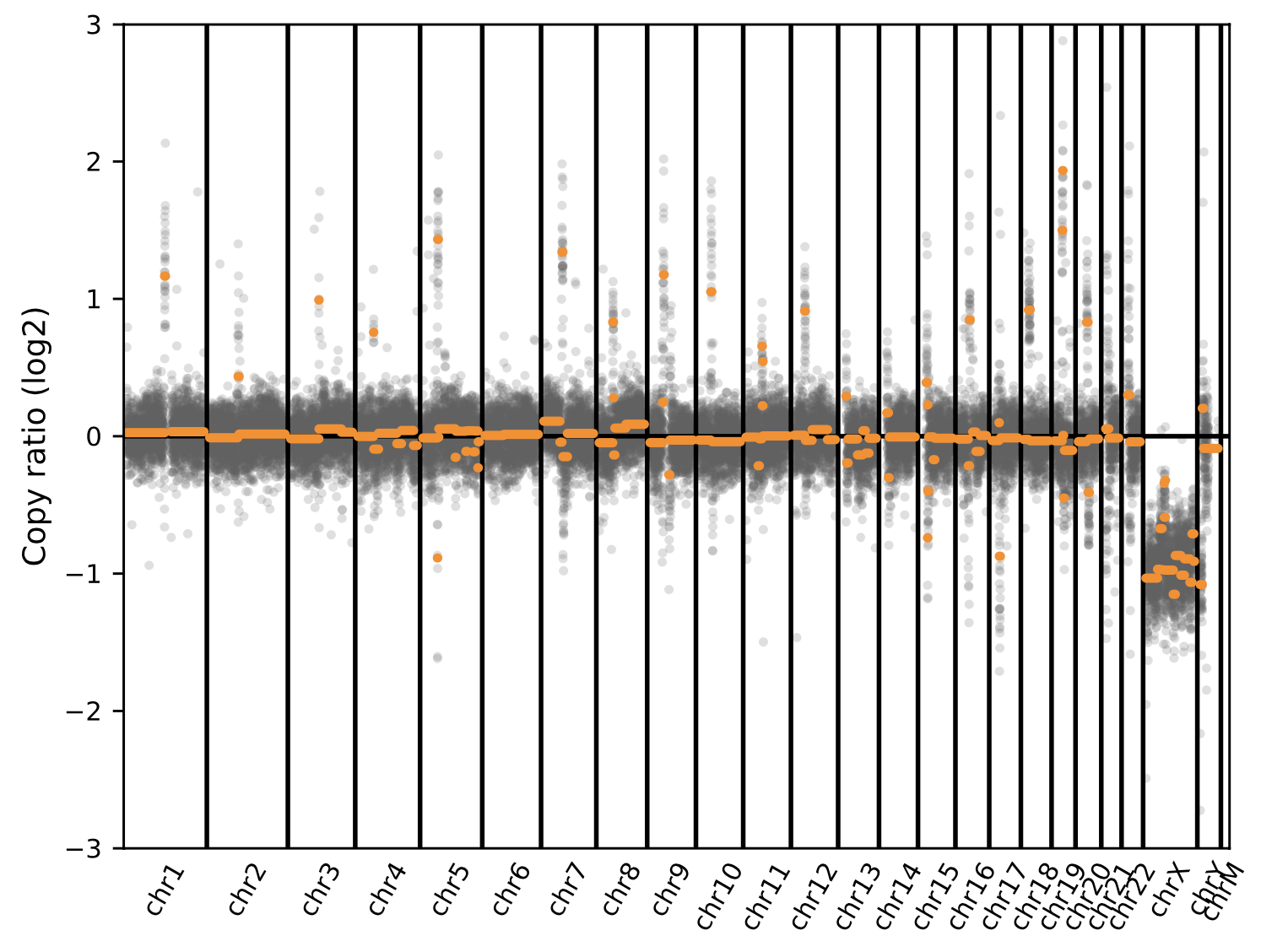 | 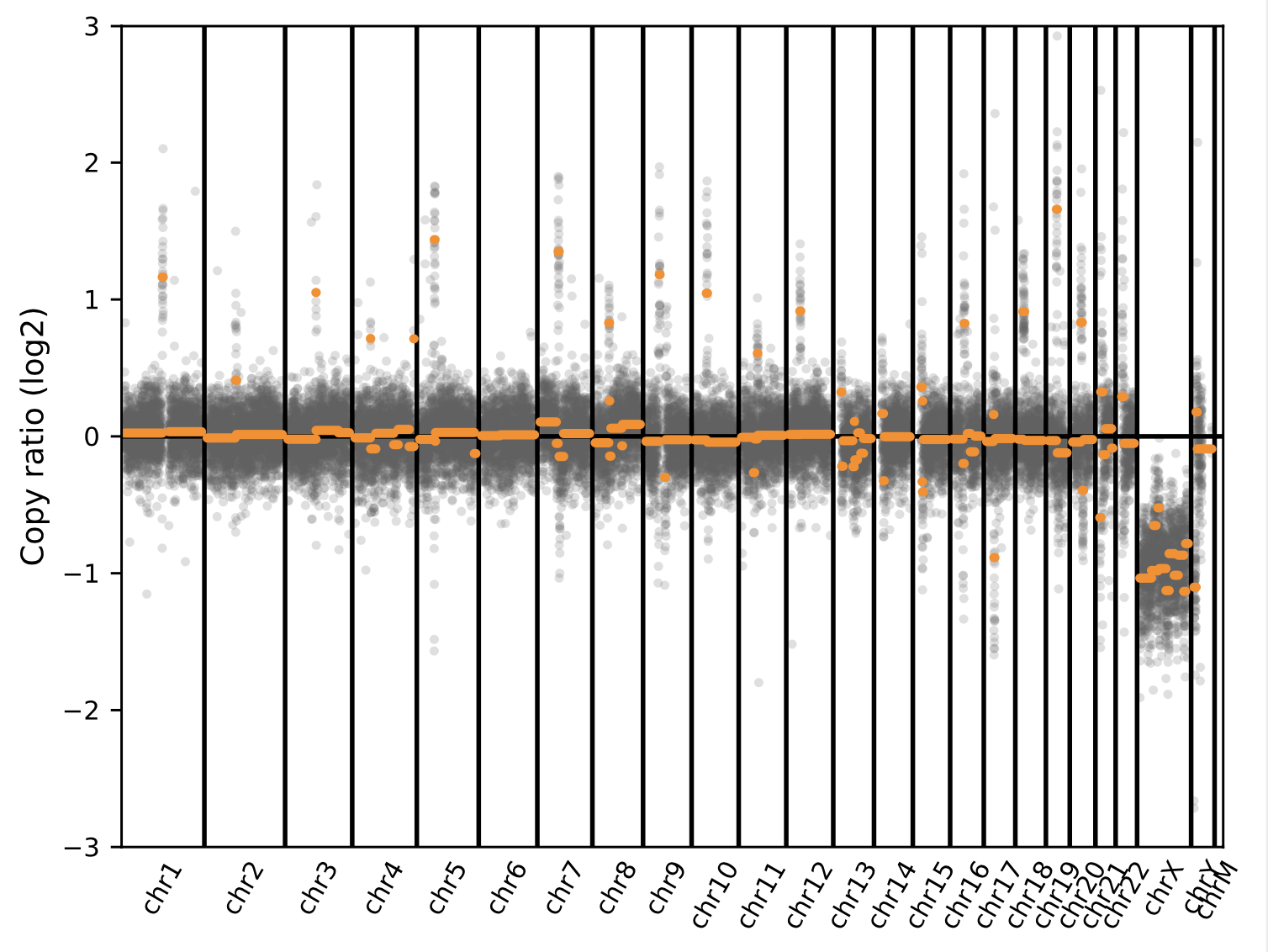 |
| --- | --- |
| 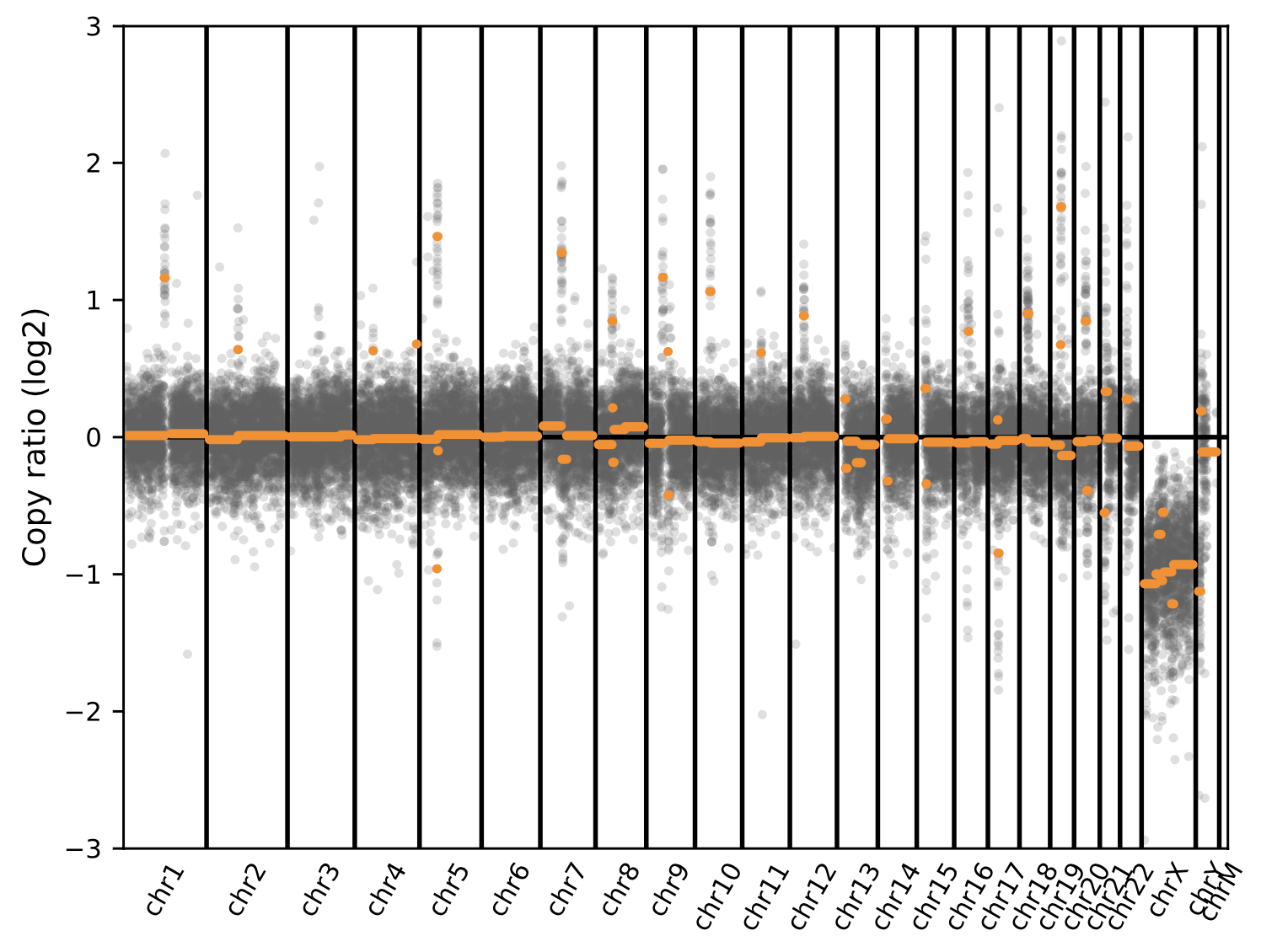 | 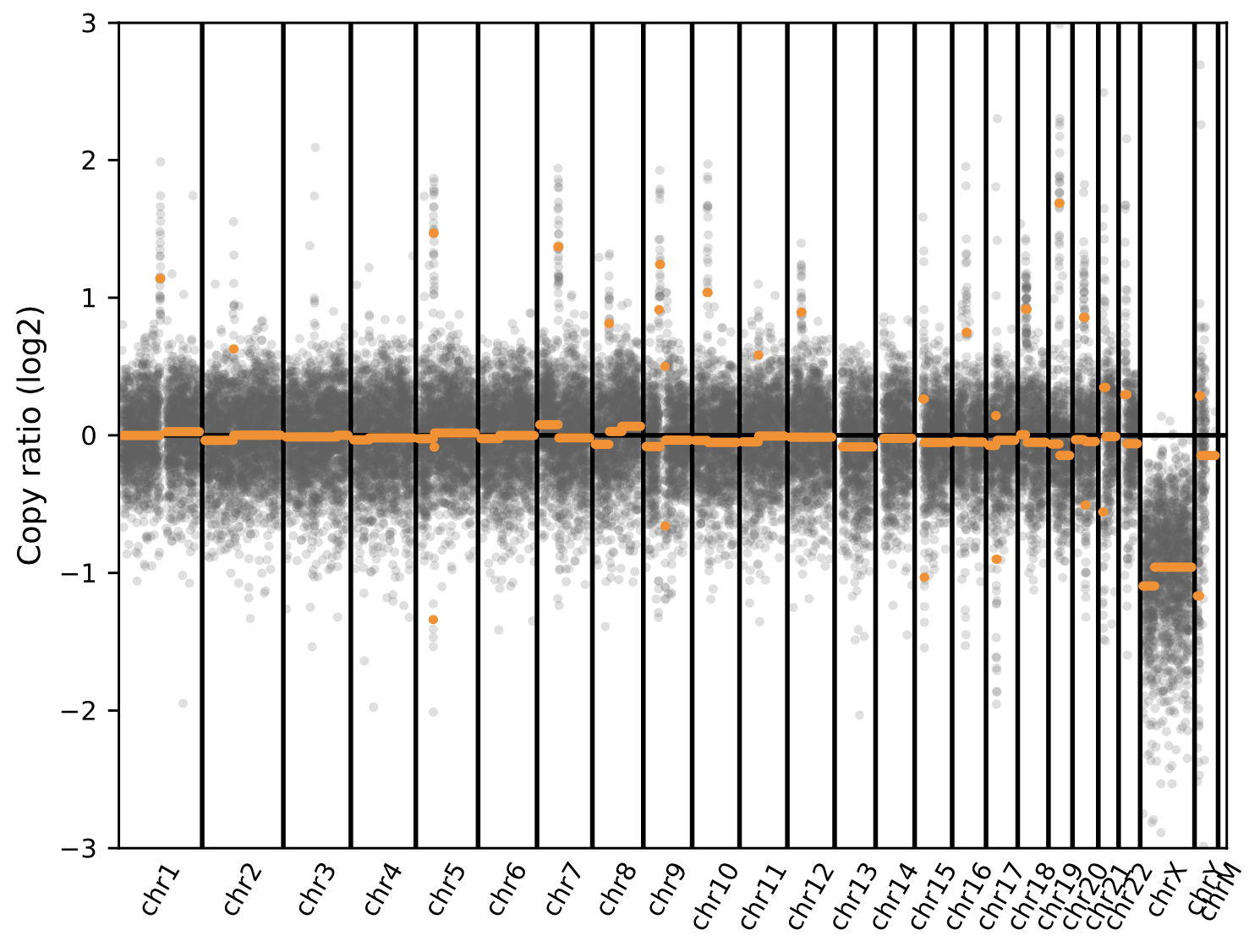 |
| 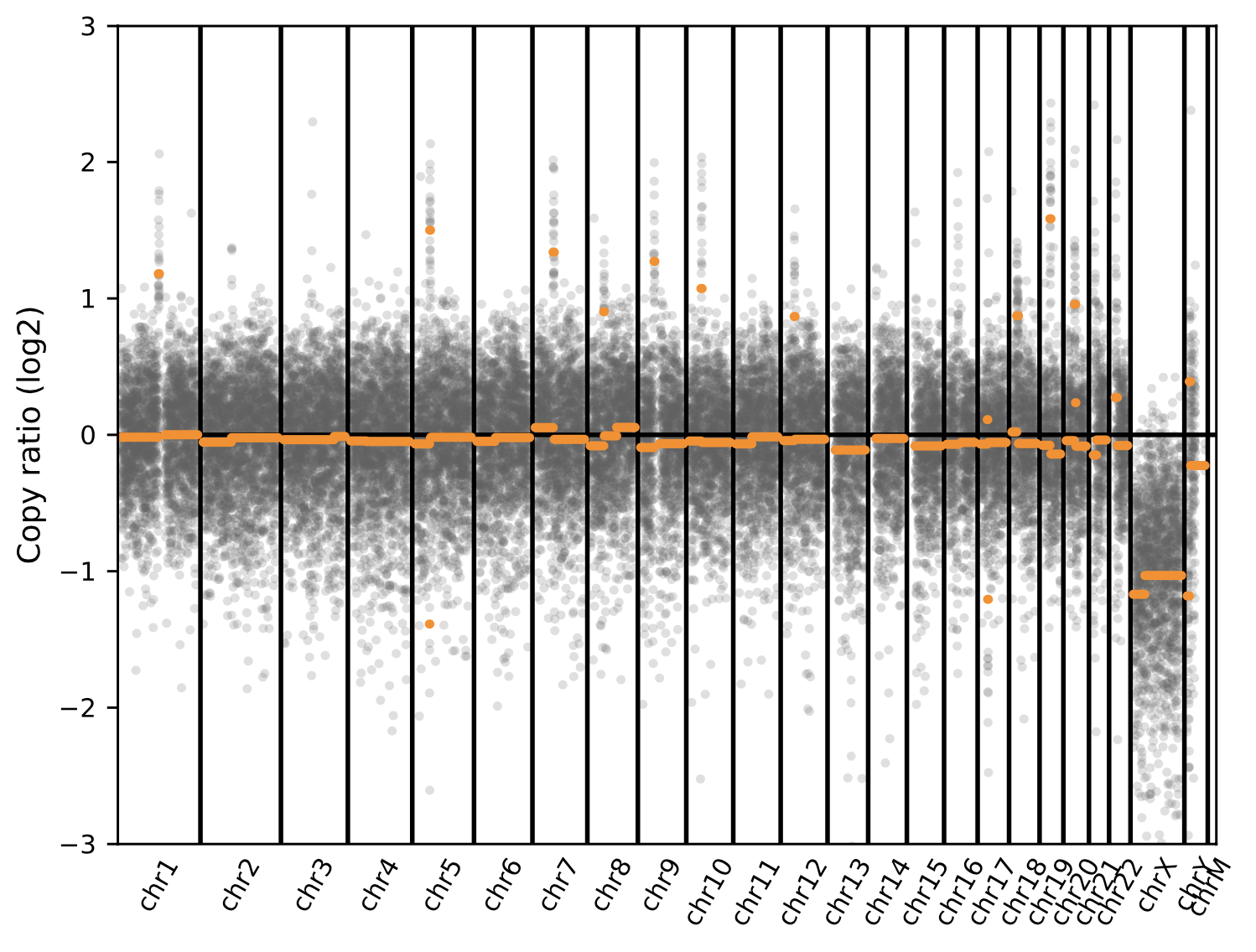 | 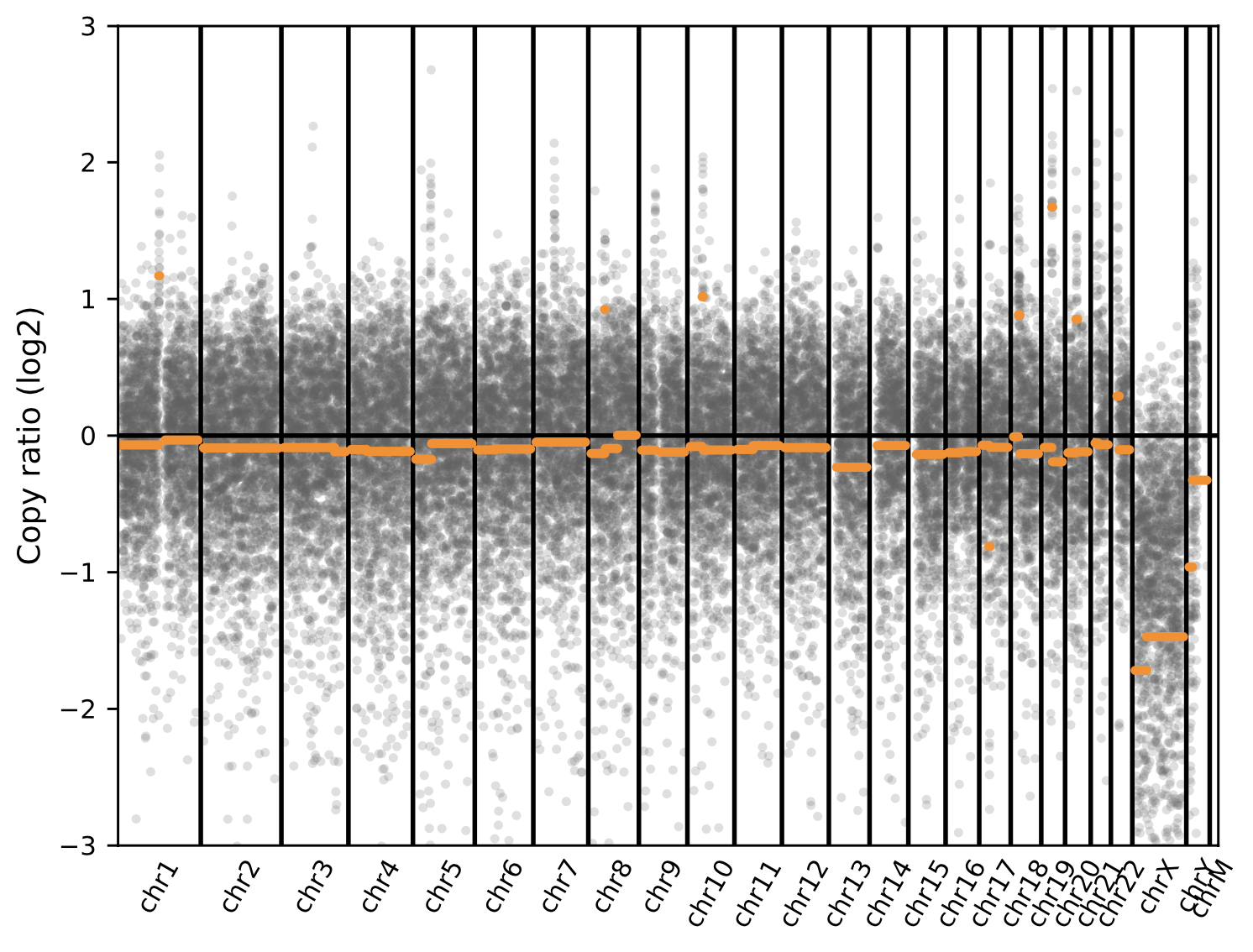 |

From left to right, top down: 1, 2, 4, 8, 16, 32-fold computational downsampling of the original sample reads at 7.9 M paired end reads. The results are interpretable down to 248 thousand paired reads. A blinded positive call was made when there was no downsampling and at a 4-fold dilution.

Read downsampling of PC63

| 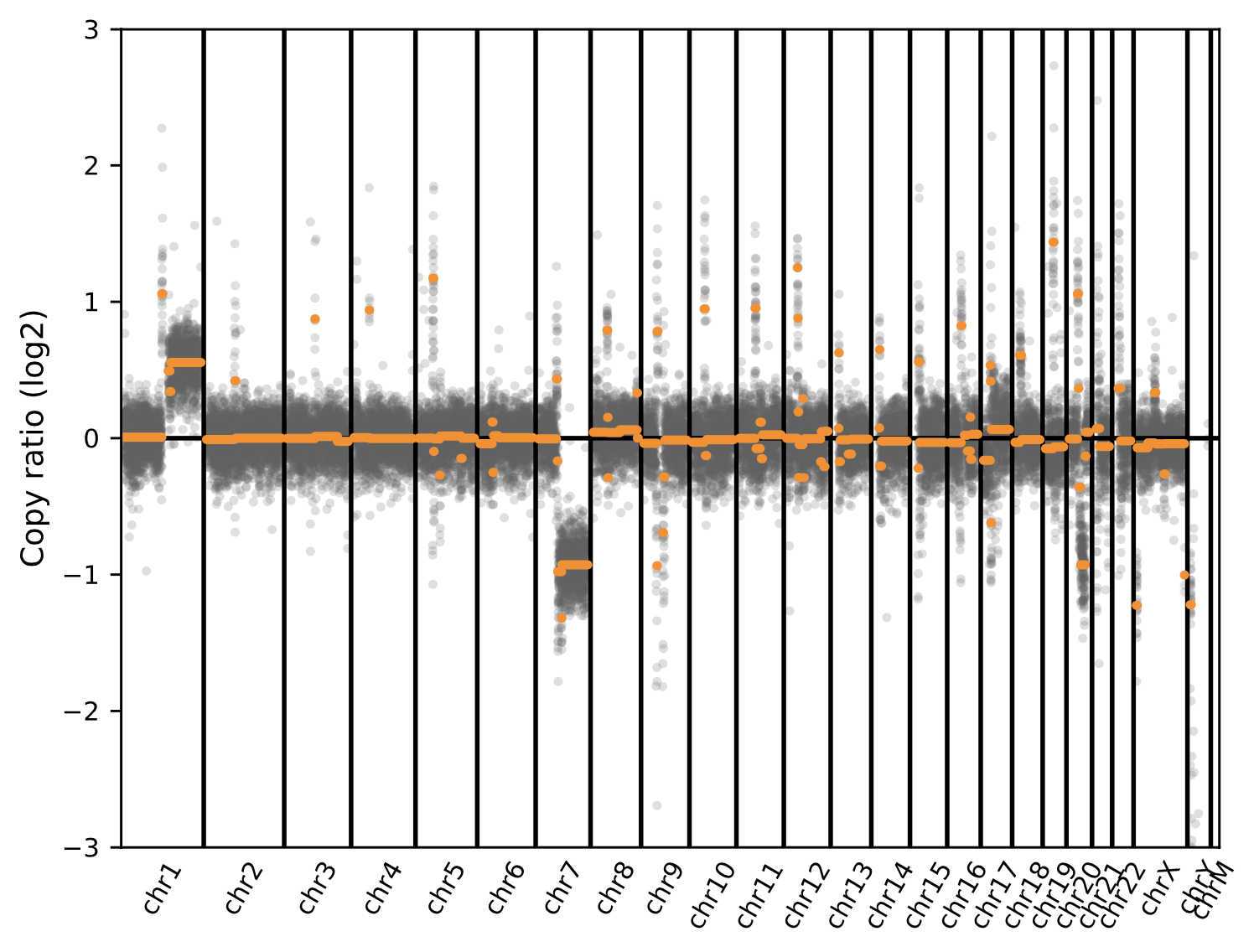 | 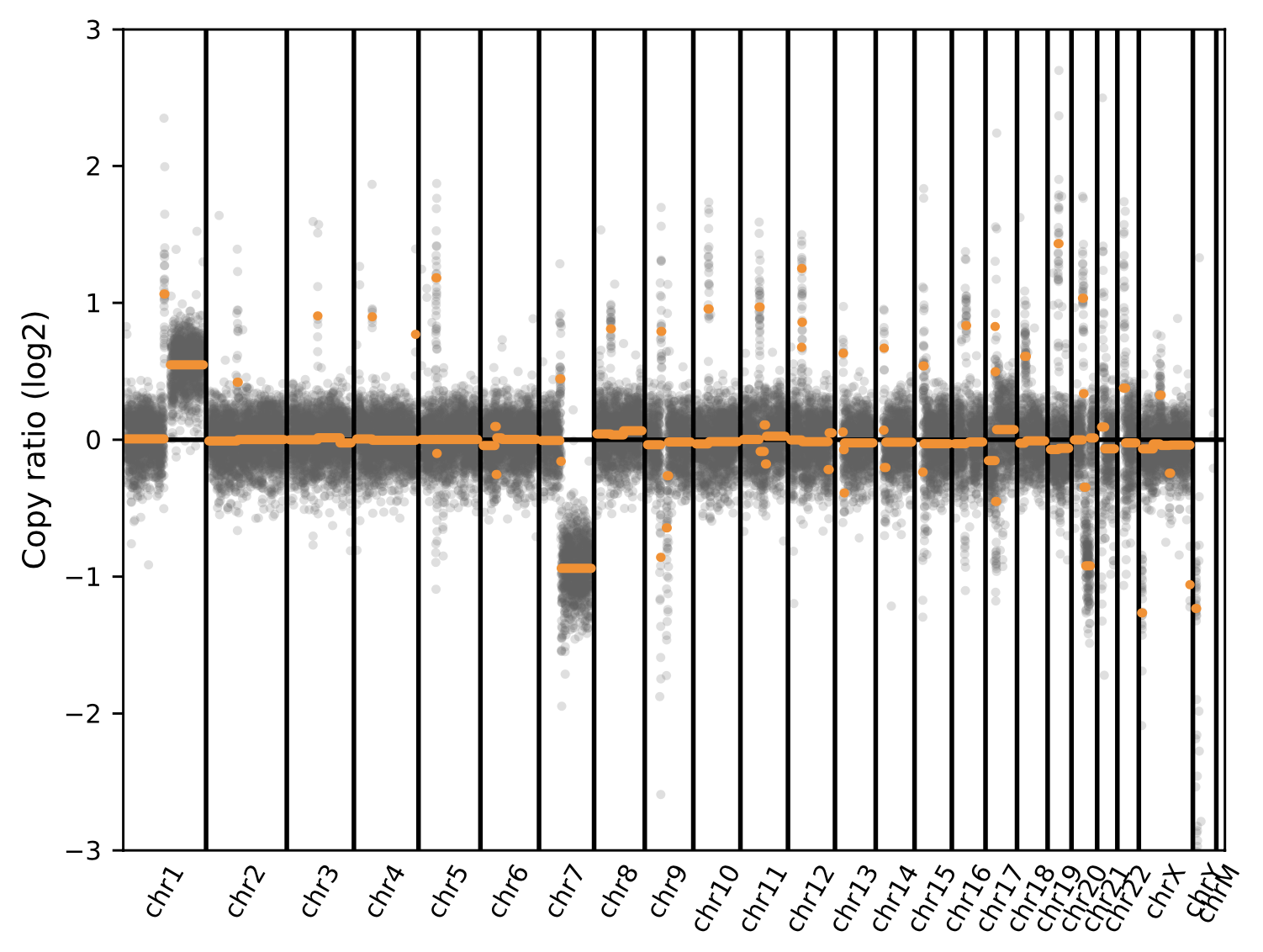 | 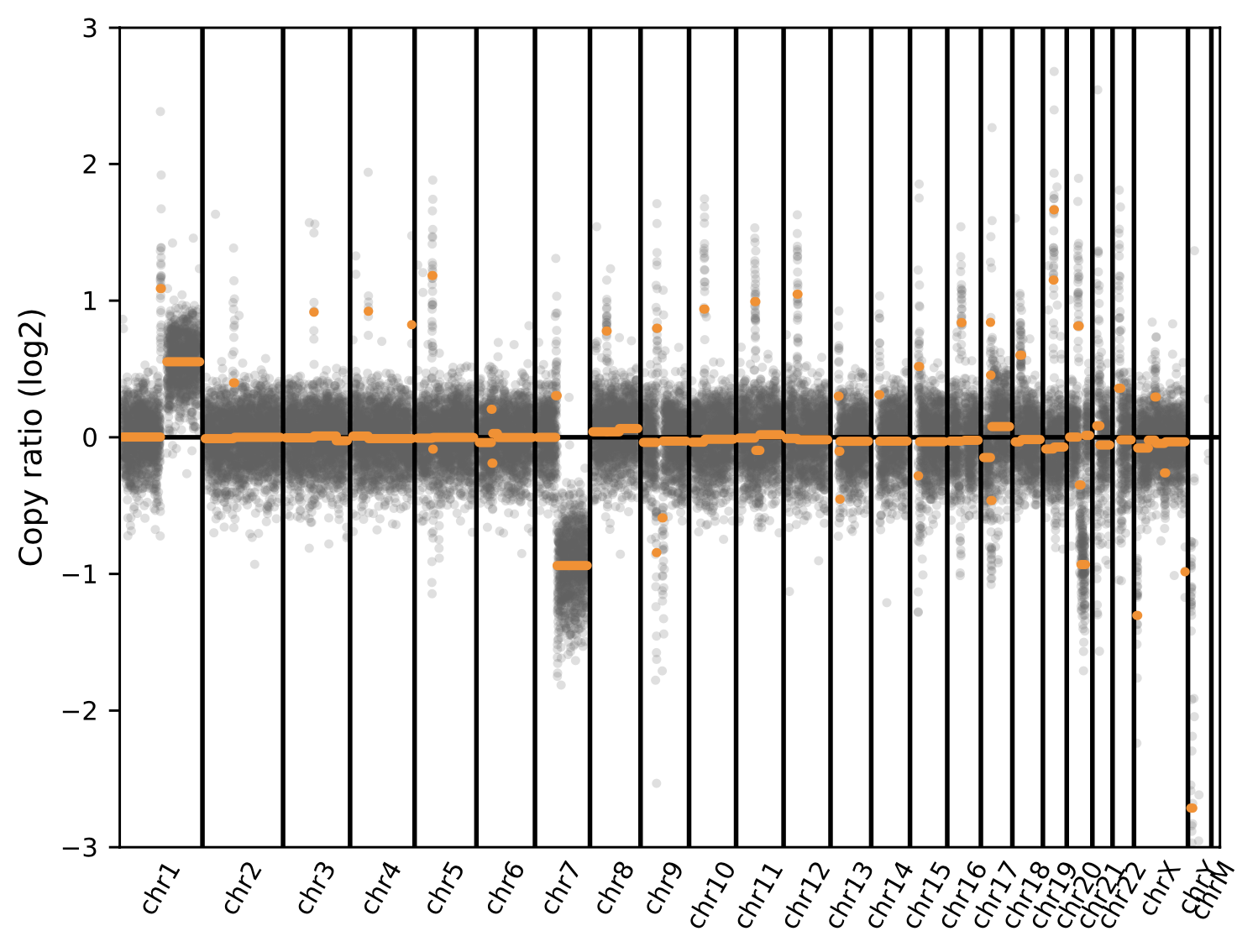 | 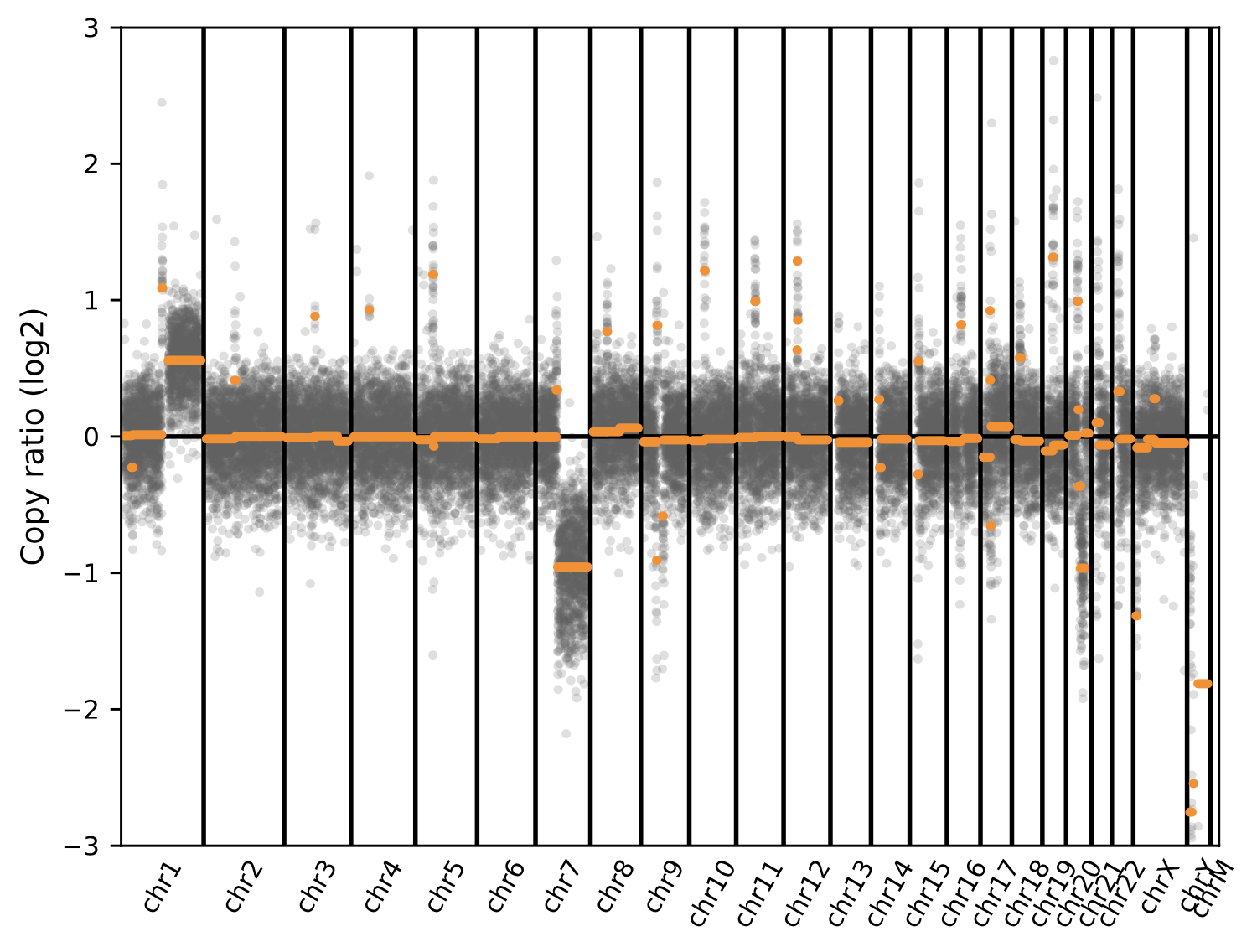 |
| --- | --- | --- | --- |
| 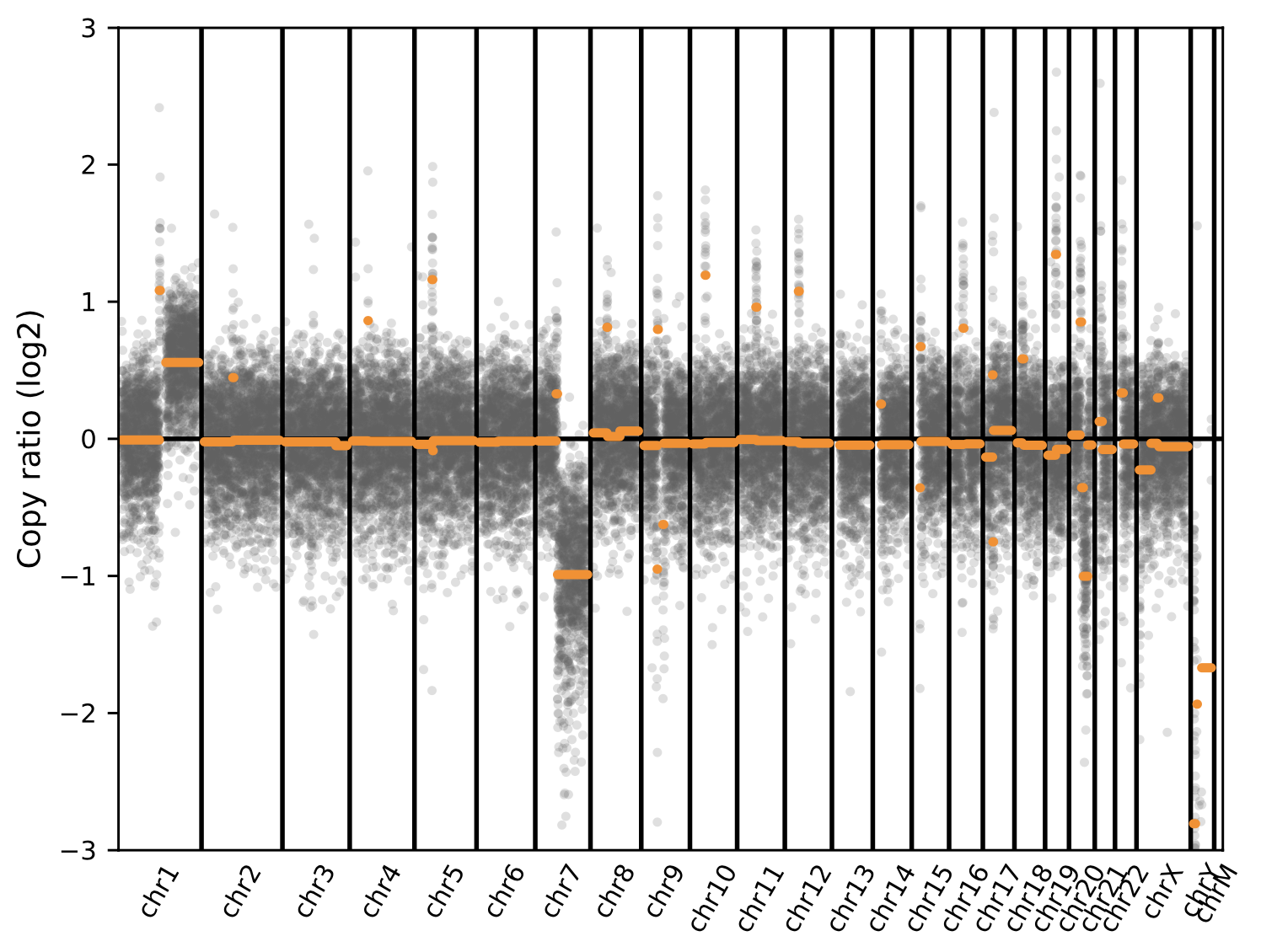 | 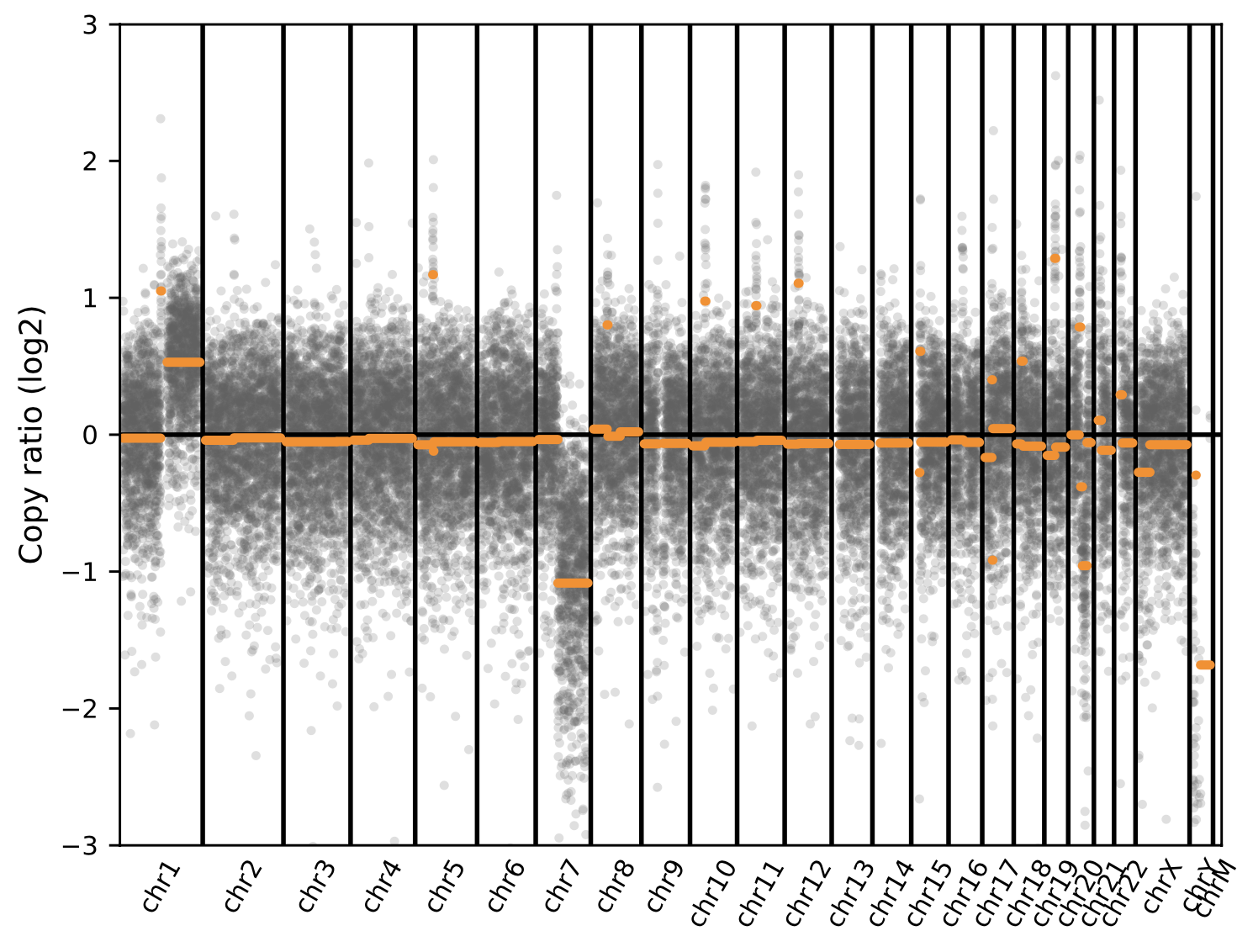 | 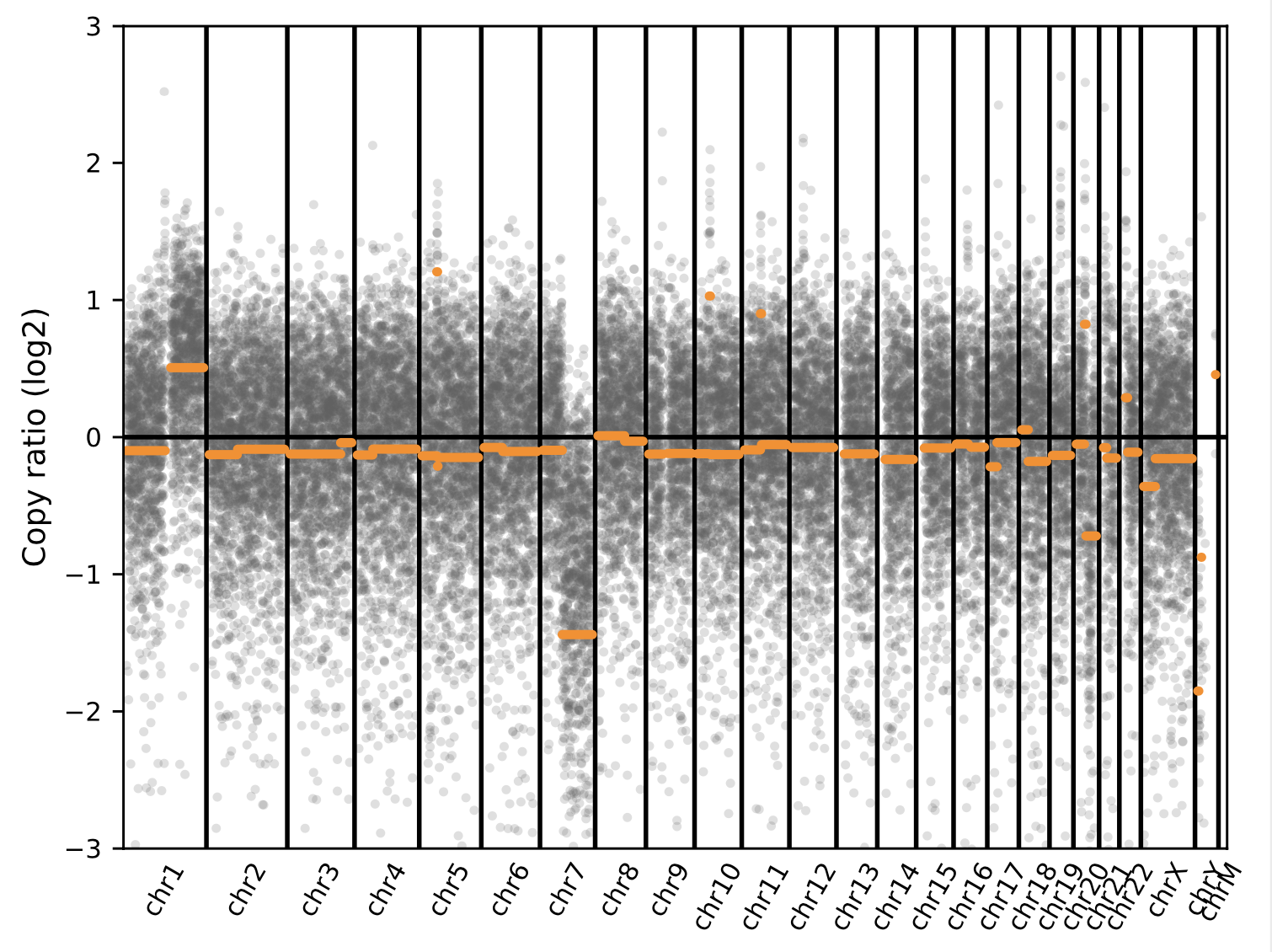 | 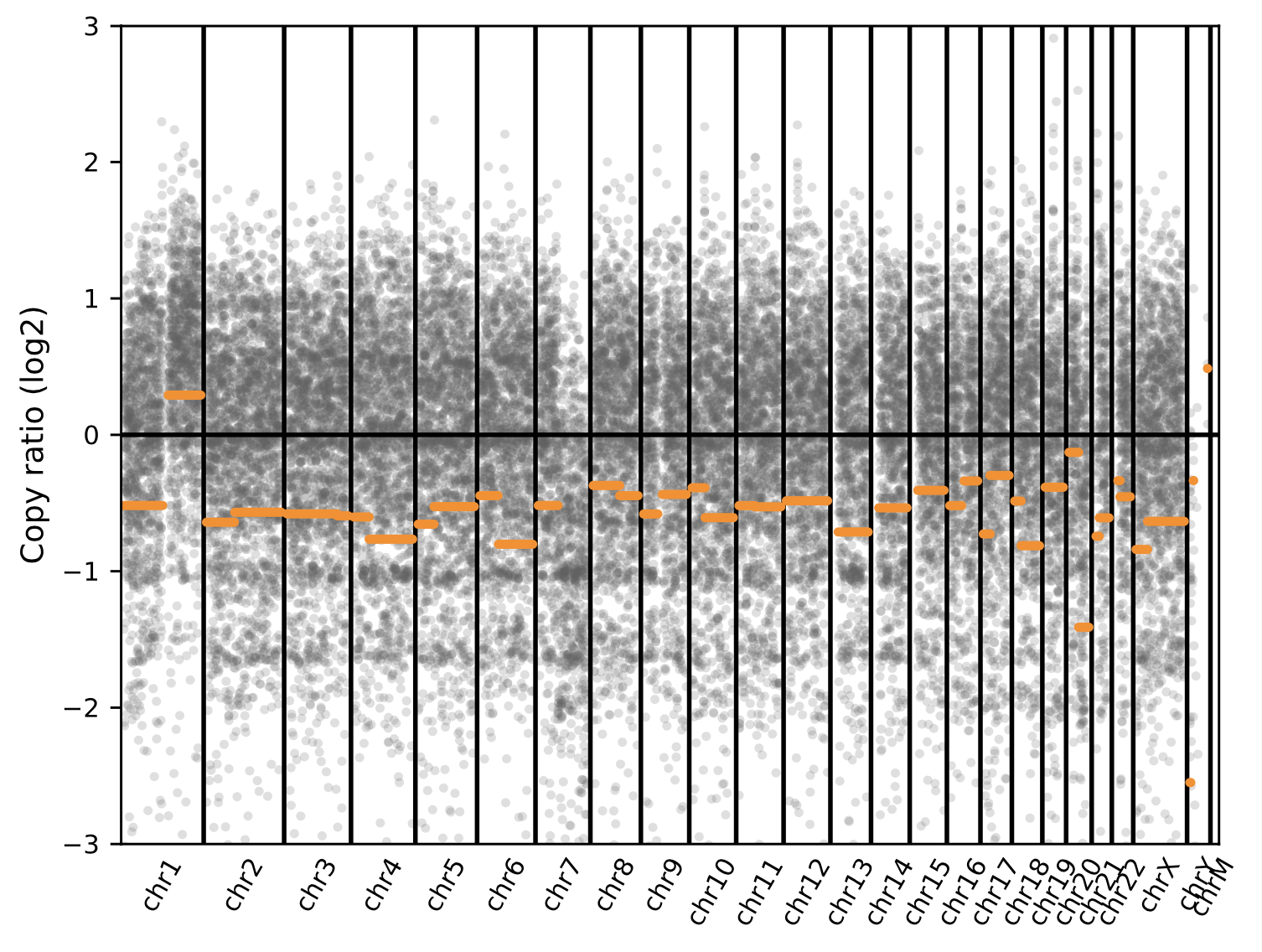 |

From left to right, top down: 1, 2, 4, 8, 16, 32, 64, 128-fold computational downsampling of the original sample reads at 13.3 M paired end reads. The results are interpretable down to 209 thousand paired reads. A blinded positive call was made when there was no downsampling and at a 64-fold dilution or 209 thousand paired reads.

**EBV (Epstein Barr Virus / Human Herpesvirus 4) Length Distributions**

PC13: Angioimmunoblastic T-cell lymphoma. EBV at 136 RPM (orange line). Human genome hg38 version 23 (blue line). Negative by CNV.


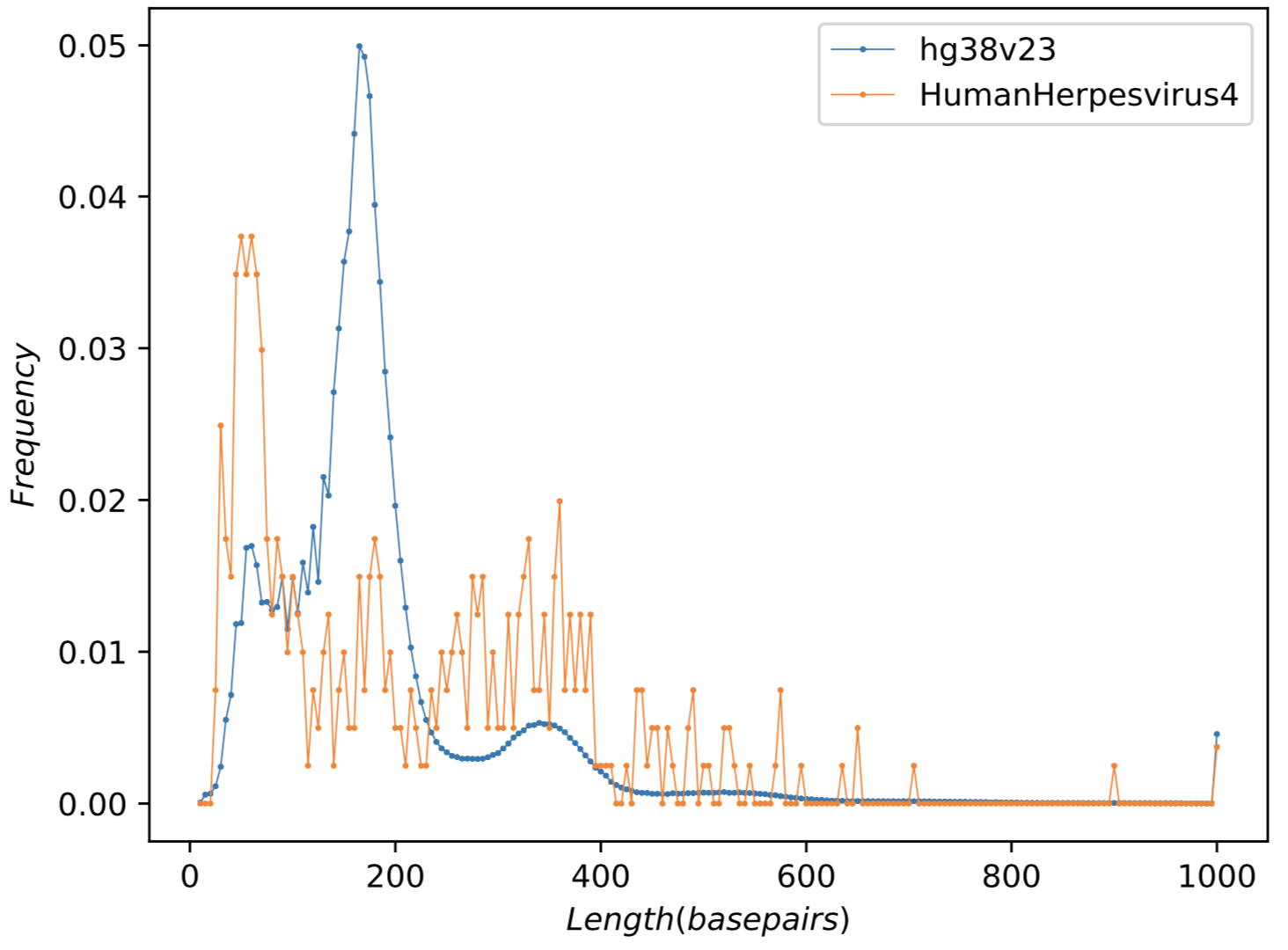


EBV DNA Size Ratio: 0.97

Interpretation: EBV DNA Size Ratio less than 9.1 associated with malignancy. Partial overlap of human and EBV/HHV4 length distribution.

PC42: Lymphoproliferative disorder (working diagnosis). EBV 134 RPM (orange line). Human genome hg38 version 23 (blue line).

Not biopsy proven, but had working diagnosis of a malignancy with high clinical suspicion of lymphoproliferative disorder +/- tuberculosis.


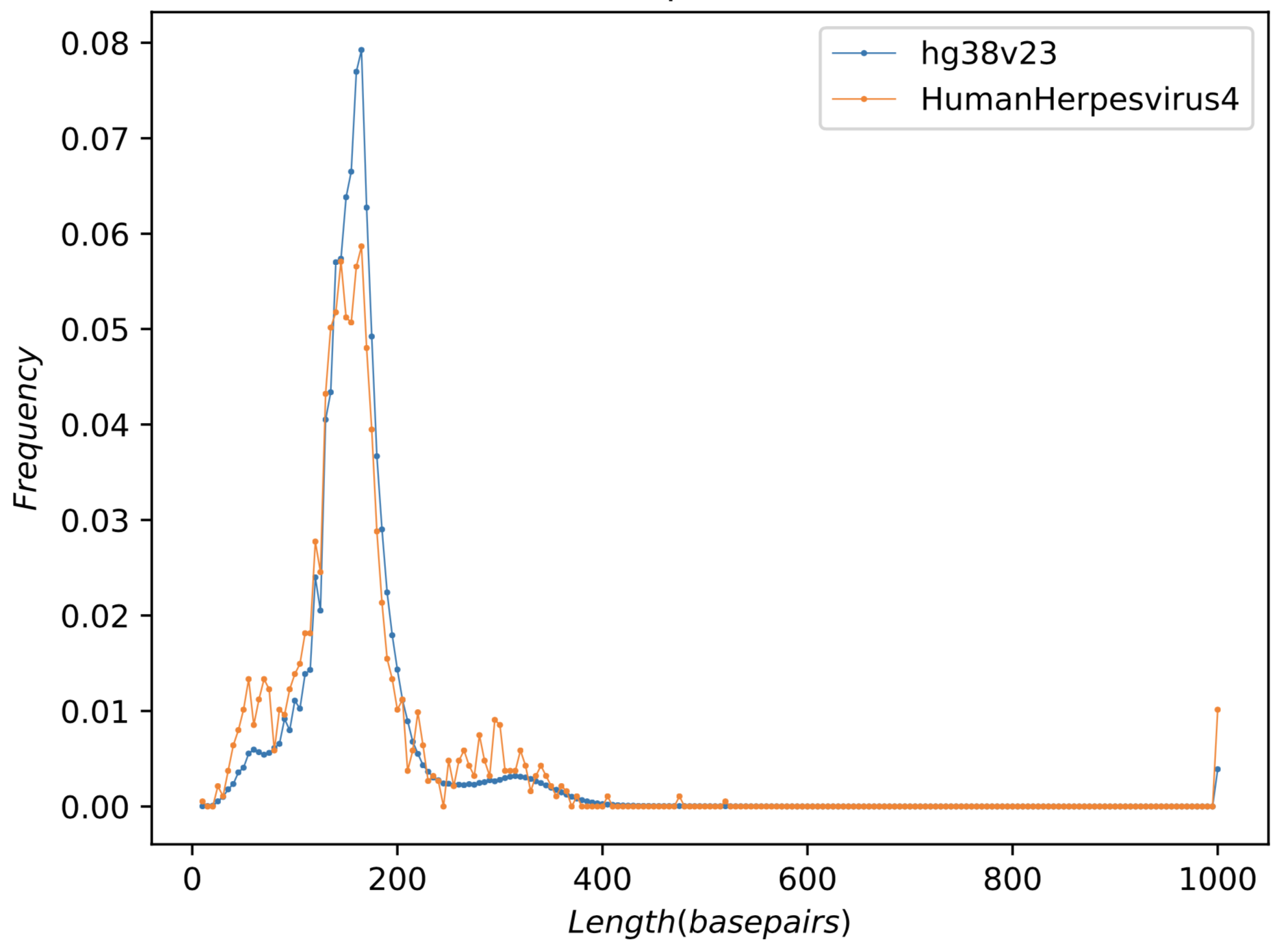


EBV DNA Size Ratio: 1.3

Interpretation: EBV DNA Size Ratio less than 9.1 associated with malignancy. Near complete overlap of human and EBV/HHV4 length distribution.

3095: Unclear cause

This is a transplant patient with a one year of low level of EBV viremia.


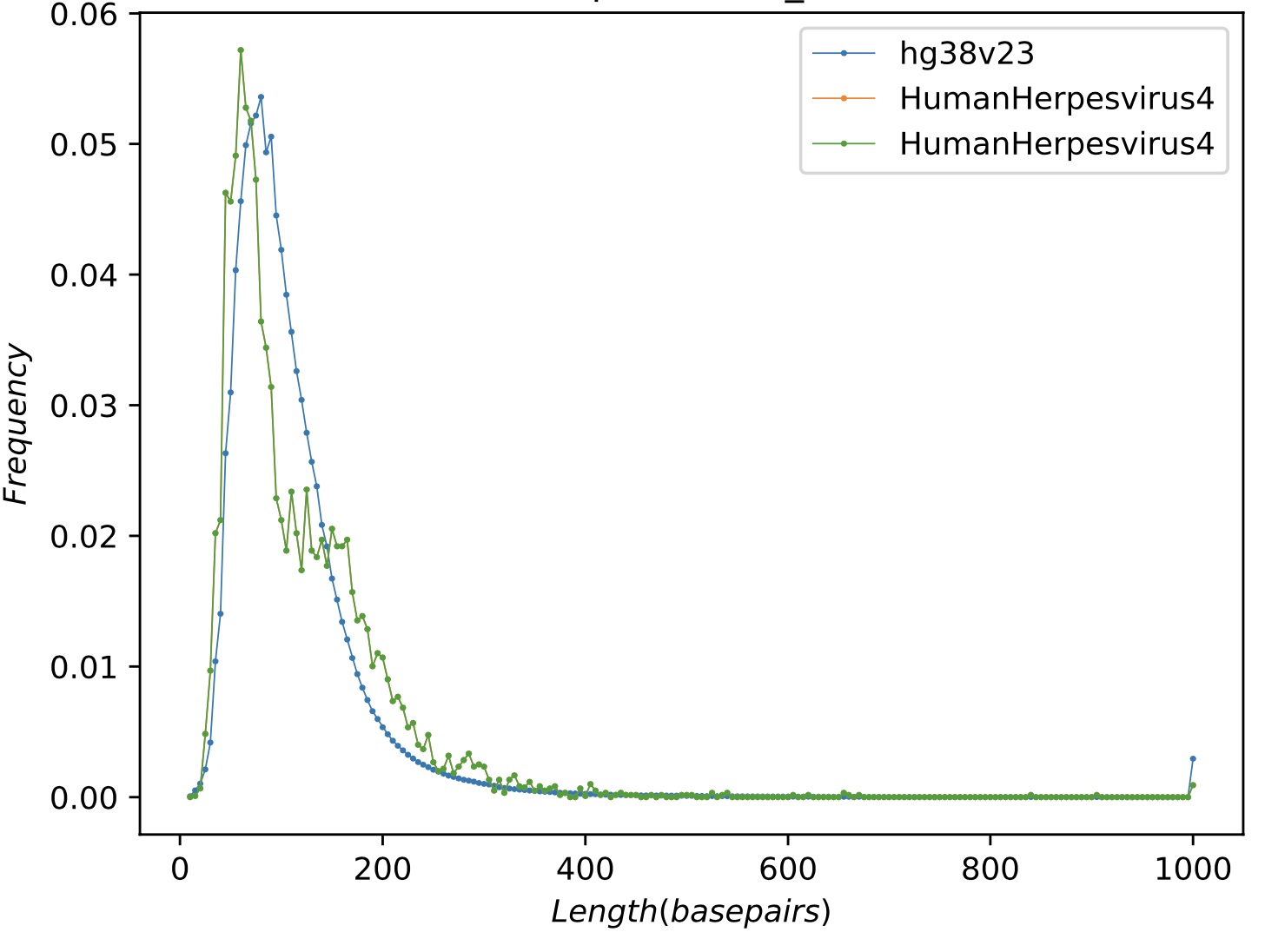


EBV DNA Size Ratio: 0.6

Interpretation: EBV DNA Size Ratio less than 9.1 associated with malignancy. However, we note that the human length distribution is degraded here, and this analysis may not be valid.
