# Supplementary figures and images for: Detection of cryptogenic malignancies from metagenomic whole genome sequencing of body fluids
Source: Genome Med. 2021 Jun 1;13:98. doi: 10.1186/s13073-021-00912-z (PMC8167833; doi:10.1186/s13073-021-00912-z)

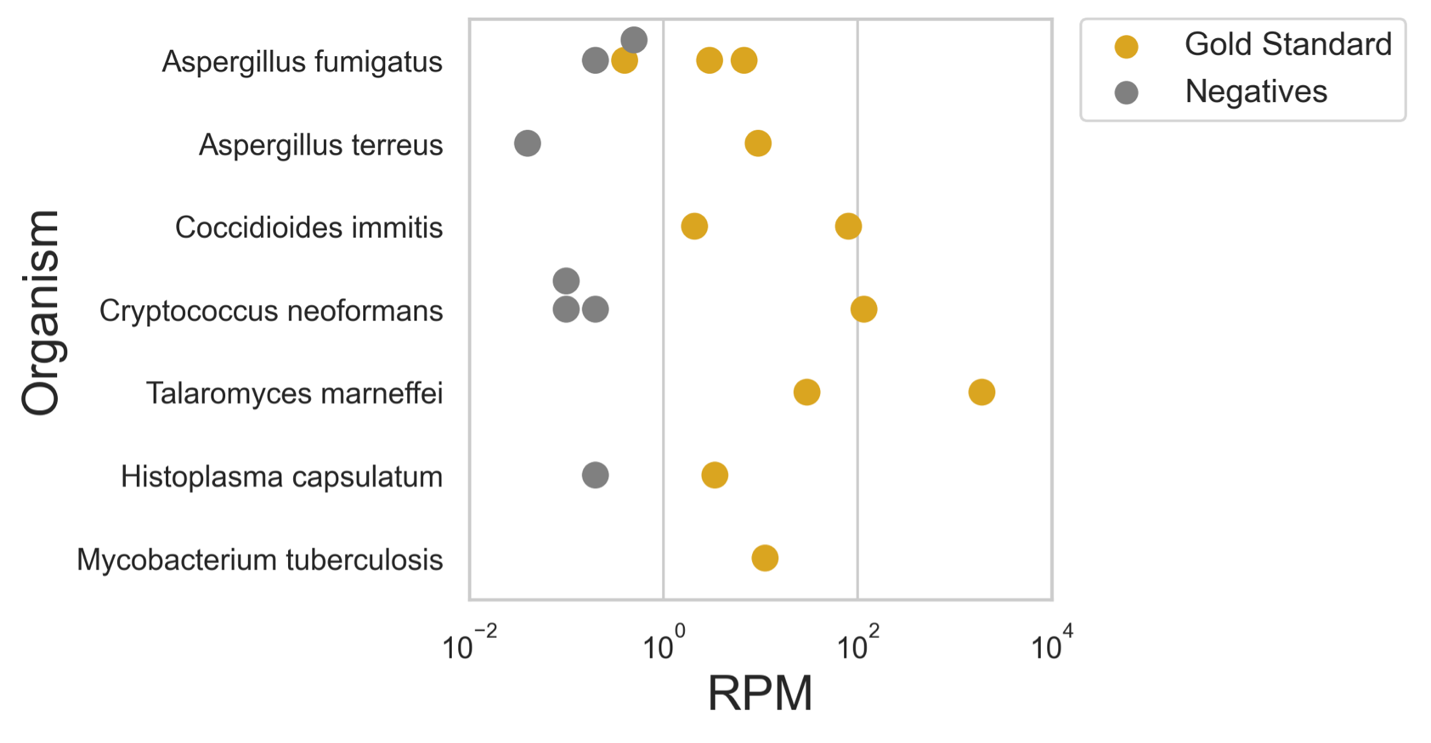


**Figure S1:** Microbiological cases with overlapping features with cancer presentations

Supplement: Supplementary file 6 — Additional file 6: Figure S1. Microbiological cases with overlapping features with cancer presentations. [file 13073_2021_912_MOESM6_ESM.docx]
